# Harziachalasins A–G, polycyclic-fused cytochalasins from the endophytic fungus *Trichoderma harzianum* MLJ-4 with HIV latency reversal activity

**DOI:** 10.1007/s13659-025-00572-1

**Published:** 2026-01-11

**Authors:** Yan-Jiang Zhang, Xian-Yuan Yang, Yi-Fan Fu, Qiong Liao, Jia-Qian Chen, Xian-An Chen, Dong Huang, Tao Yuan, Xin Chen, Sheng Yin, Gui-Hua Tang

**Affiliations:** 1 School of Pharmaceutical Sciences, Sun Yat-Sen University, Guangzhou, 510006 People’s Republic of China; 2 Laboratory Animal Center, Sun Yat-Sen University, Guangzhou, 510006 People’s Republic of China; 3 School of Health, Jiangxi Normal University, Nanchang, 330022 People’s Republic of China; 4 School of Life Science and Technology, Wuhan Polytechnic University, Wuhan, 430023 People’s Republic of China

**Keywords:** Endophytic fungus, *Trichoderma harzianum*, Polycyclic-fused cytochalasins, HIV latency reversal activity

## Abstract

Seven new polycyclic-fused cytochalasins (CYTs), harziachalasins A–G (**1**–**7**), together with three known analogues (**8**–**10**) were isolated from the solid culture of the endophytic fungus *Trichoderma harzianum* MLJ-4, which was originally isolated from the leaves of *Asclepias curassavica*. The planar and absolute structures of all new compounds were determined on the basis of extensive spectroscopic data (1D, 2D NMR and HR-ESI–MS), NMR calculations with DP4 + probability analysis, and theoretical simulations of ECD spectra. Compound **1** represents the first example of 5/6/6 tricyclic CYT featuring a 2-methyl-4-oxopentyl side chain at the C-14 position. This novel architecture originates from a 5/6/6/7 tetracyclic CYT precursor through sequential oxidative cleavage of the C-19–C-20 bond followed by decarboxylative elimination of C-19. Compound **2** features an unprecedented 5/6/6/6/7 pentacyclic scaffold incorporating a 1,3-dioxane moiety, may be constructed by the acetalization of the 7-OH and 13-OH on a 5/6/6/7 tetracyclic CYT with acetaldehyde. Compounds **1**–**10** were screened for HIV latency reversal activity using J-Lat A72 and J-Lat 10.6 cell models. Compound **4** showed strong activity, with half-maximal effective concentrations (EC_50_) values of 2.68 μM (J-Lat A72 cells) and 2.99 μM (J-Lat 10.6 cells), demonstrating consistent potency. Mechanistic studies revealed **4** activated the NF-*κ*B pathway to reverse HIV latency, offering insights for new therapeutic strategies targeting this pathway.

**Graphic Abstract:**

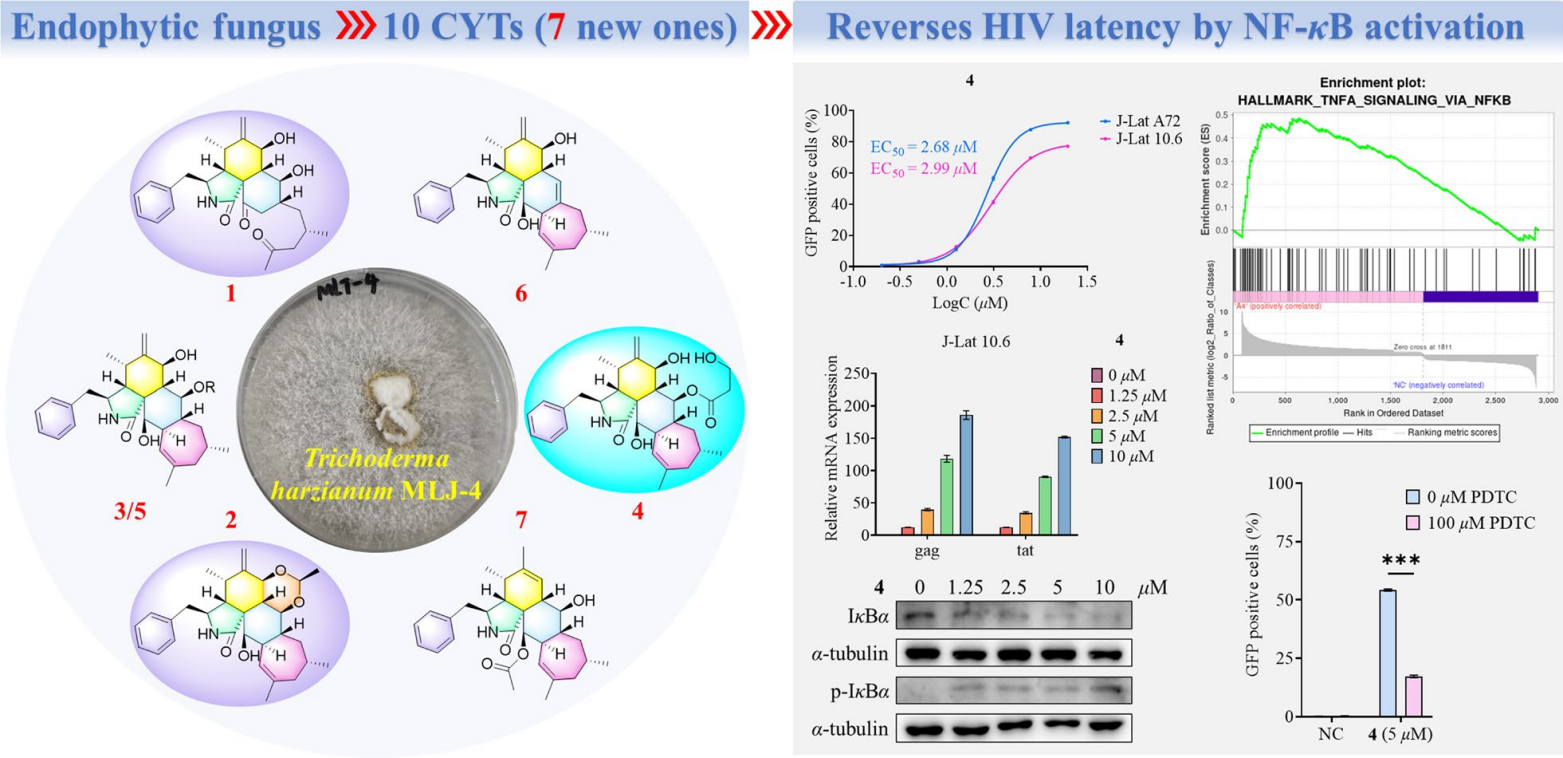

**Supplementary Information:**

The online version contains supplementary material available at 10.1007/s13659-025-00572-1.

## Introduction

Fungi represent an underexploited reservoir of structurally diverse natural products with significant bioactivities, serving as invaluable sources for both therapeutic development and molecular probes for target discovery [1]. Among these metabolites, cytochalasins (CYTs) constitute a distinctive class of fungal-derived compounds biosynthesized via polyketide synthase-nonribosomal peptide synthetase (PKS-NRPS) pathways, integrating amino acids and polyketide precursors [2, 3]. Characterized by an isoindole moiety fused with a macrocyclic ring system, their structural complexity arises from: (1) Varied amino acid incorporation, (2) PKS-NRPS-mediated chains modifications, and (3) intramolecular cyclization forming polycyclic systems [4, 5]. Recent advances include the discovery of novel scaffolds [6–17], mechanistic studies [18, 19], and synthetic breakthroughs [5, 20–22], highlighting their pharmaceutical potential.

**Fig. 1:**
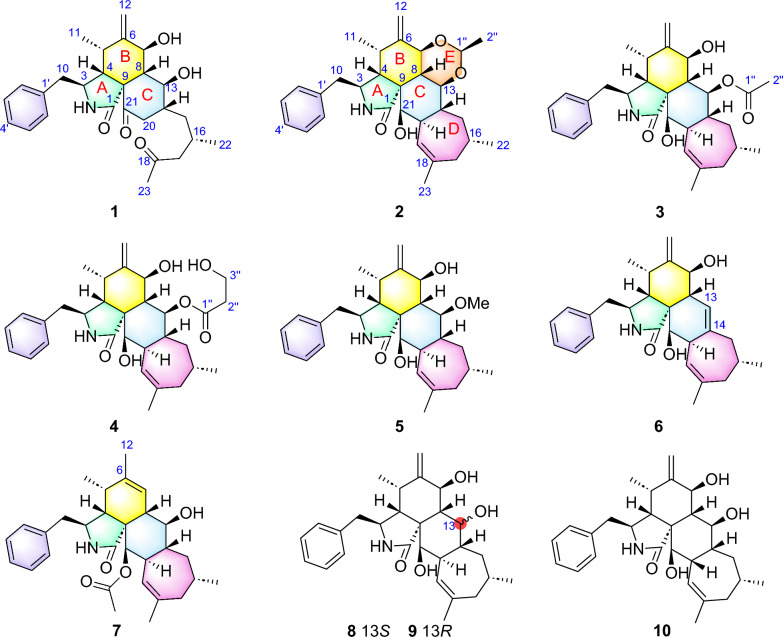
The structures of polycyclic-fused cytochalasins (CYTs)

Despite antiretroviral therapy (ART) effectively suppressing HIV replication, persistent viral reservoirs remain the primary barrier to a cure [23, 24]. Latency-reversing agents (LRAs) have emerged as a strategic therapeutic approach to activate latent HIV-1, enabling immune clearance [25–27]. Notably, naturally occurring CYTs−highly oxygenated polycyclic metabolites−demonstrate compelling anti-HIV activities, positioning them as promising LRA candidates [28, 29]. Building upon our ongoing exploration of fungal-derived bioactive natural products [30–37], the remarkable structural diversity and therapeutic potential of CYTs emerged as a key research focus in our group. In particular, the strain *Trichoderma harzianum* MLJ-4 was selected for detailed investigation based on its promising metabolic profile. This study led to the isolation of seven new CYTs, harziachalasins A–F (**1**–**7**) (Fig. 1), along with three known analogs (**8**–**10**) (Fig. 1), phomopsischalin B (**8**) [6], phomopsischalin A (**9**) [6], and ueckerchalasin A (**10**) [38]. Herein, we describe their isolation, structural elucidation, and evaluation of HIV latency reversal activity.

## Results and discussion

### Structure elucidation

Harziachalasin A (**1**) was obtained as white, amorphous powder. Its molecular formula C_27_H_35_NO_5_ was determined by the HR-ESI-MS ion at *m/z* 476.2384 [M + Na]^+^ (calcd. 476.2407), requiring 11 degrees of unsaturation. The IR absorption bands revealed the presence of hydroxyl (3330 cm^−1^), carbonyl (1676 cm^−1^), and phenyl (1600 and 1456 cm^−1^) functionals. The ^1^H NMR spectrum recorded in CDCl_3_ (Table 1) displayed one mono-substituted benzene ring in the aromatic region [*δ*_H_ 7.19 (2H, d, *J* = 7.3 Hz), 7.24 (1H, t, *J* = 7.3 Hz), and 7.31 (2H, t, *J* = 7.3 Hz)], an terminal methylene [*δ*_H_ 5.01 (1H, s) and 5.20 (1H, s)], two sp^3^ oxygenated methines [*δ*_H_ 4.46 (1H, d, *J* = 9.2 Hz) and 4.98 (1H, td, *J* = 9.9, 1.8 Hz)], three sp^3^ methyl groups [*δ*_H_ 0.89 (3H, d, *J* = 6.6 Hz), 0.93 (3H, d, *J* = 6.6 Hz), and 2.15 (3H, s)], and other aliphatic protons. The ^13^C and DEPT NMR experiments indicated 27 carbons including two ketocarbonyls [*δ*_C_ 209.7 (C) and 203.9 (C)], an amide carbonyl [*δ*_C_ 172.1 (C)], a mono-substituted benzene ring [*δ*_C_ 126.9 (CH), 128.8 (CH × 2), 129.3 (CH × 2), and 137.6 (C)], one exocyclic double bond [*δ*_C_ 113.6 (CH_2_) and 148.5 (C)], eight sp^3^ methines [including two oxygenated ones at *δ*_C_ 73.9 (CH) and 72.5 (CH)], three sp^3^ methyls [*δ*_C_ 12.6 (CH_3_), 20.3 (CH_3_), and 30.5 (CH_3_)], a sp^3^quaternary carbon [*δ*_C_ 62.3 (C)], and four sp^3^ methylenes. As eight of 11 degrees of unsaturation were occupied by two carbonyls, an amide carbonyl, a benzene ring, and a double bond, the remaining three degrees of unsaturation indicated that **1** was a tricyclic skeleton. The above-mentioned information together with the comparison of its NMR data with the co-isolated known compounds **8**–**10** [6, 38] suggested that **1** should be a tricyclic ring system derived from the type of 5/6/6/7-tetracyclic cytochalasin.

**Table 1:** ^1^H (400 MHz) and ^13^C (100 MHz) NMR Data of Compounds **1** and **2** in CDCl_3_ (*δ* in ppm)

No	1		2	
	*δ*_H_, multi. (*J* in Hz)	*δ*_C_, type	*δ*_H_, multi. (*J* in Hz)	*δ*_C_, type
1		172.1, C		176.3, C
2-NH	5.37, s		5.43, br s	
3	3.29, t (7.5)	53.6, CH	3.30, overlapped	53.3, CH
4	3.21, dd (5.8, 1.8)	43.2, CH	2.61, m	47.1, CH
5	2.66, t (6.6)	31.1, CH	2.88, m	34.0, CH
6		148.5, C		146.8, C
7	4.46, d (9.2)	73.9, CH	3.93, br d (11.4)	75.4, CH
8	2.07, t (9.6)	48.8, CH	2.26, t (10.9)	36.9, CH
9		62.3, C		50.0, C
10	a 2.77, dd (13.2, 6.1); b 2.70, dd (13.2, 8.9)	43.0, CH_2_	a 2.87, overlapped; b 2.55, m	45.7, CH_2_
11	0.89, d (6.6)	12.6, CH_3_	1.08, d (6.6)	14.1, CH_3_
12	a 5.20, s; b 5.01, s	113.6, CH_2_	a 5.39, s; b 5.18, s	115.6, CH_2_
13	4.98, td (9.9, 1.8)	72.5, CH	3.98, t (10.0)	78.1, CH
14	1.85, m	42.3, CH	1.67, m	38.6, CH
15	*α* 1.48, ddd (14.0, 8.1, 3.6); *β* 1.57, ddd (14.0, 9.8, 3.5)	38.5, CH_2_	*α* 1.06, m; *β* 2.34, d (13.8)	40.4, CH_2_
16	2.21, m	25.9, CH	1.61, m	32.1, CH
17	2.43, d (6.4)	52.0, CH_2_	a 2.08, d (14.6); b 1.92, dd (15.0, 9.1)	42.0, CH_2_
18		209.7, C		140.8, C
19			5.15, m	125.0, CH
20	2.55, dd (13.8, 4.2); 2.98, t, (13.8)	42.0, CH_2_	3.08, d (12.0)	43.7, CH
21		203.9, C	3.31, overlapped	75.0, CH
22	0.93, d (6.6)	20.3, CH_3_	0.94, d (6.7)	24.5, CH_3_
23	2.15, s	30.5, CH_3_	1.77, s	28.4, CH_3_
1′		137.6, C		137.7, C
2′/6′	7.19, d (7.3)	129.3, CH	7.15, d (7.0)	129.5, CH
3′/5′	7.31, t (7.3)	128.8, CH	7.32, t (7.2)	128.9, CH
4′	7.24, t (7.3)	126.9, CH	7.26, t (7.1)	127.1, CH
1′′			4.82, q (5.1)	99.4, CH
2′′			1.36, d (5.1)	21.3, CH_3_
7-OH	3.95, br s			
13-OH	4.35, br s			
21-OH			1.78, br s	

**Fig. 2:**
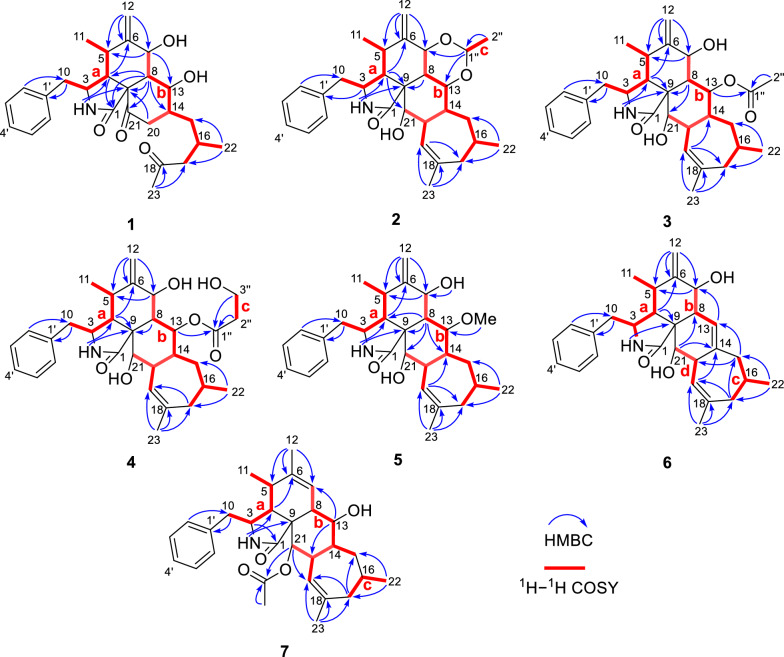
^1^H−^1^H COSY and key HMBC correlations of **1**−**7**

The planar structure of **1** was established by extensive analysis of 2D NMR spectra. As shown in Fig. 2, the observed two ^1^H–^1^H COSY spin systems: H-10/H-3/H-4/H-5/H-11 and H-7/H-8/H-13/H-14(/H-20)/H-15/H-16/H-17, suggested the present of two key fragments, **a** (C-10–C-3–C-4–C-5–C-11) and **b** (C-7–C-8–C-13–C-14(–C-20)–C-15–C-16–C-17). By analysis of the HMBC correlations (Fig. 2), from 2-NH to C-4 and C-9, H-4 to C-6 and C-1, H-7 to C-5 and C-13, H-8 to C-1, C-4, C-9, C-14, and C-21, H_2_-12 to C-5, C-6, and C-7, proved that fragment **a**, the exocyclic double bond, part of moiety **b**, one ketocarbonyl, the only sp^3^ quaternary carbon, and the amide carbonyl group construct a 5/6/6 tricyclic core. The HMBC correlations from H_3_-23 to another ketocarbonyl (C-18) and C-17 indicated that fragment **b** ended with an acetyl group. Thus, a 2-methyl-4-oxopentyl side chain was determined to be connected to ring C, rather than being fused with a 7-membered ring as in the skeleton of 5/6/6/7-tetracyclic cytochalasin. Furthermore, key HMBC correlations from H-2′/H-6′ to C-10 and form H-10 to C-1′ confirmed the connection of the mono-substituted benzene ring to ring A. Here, the planar structure of **1** was elucidated.

**Fig. 3:**
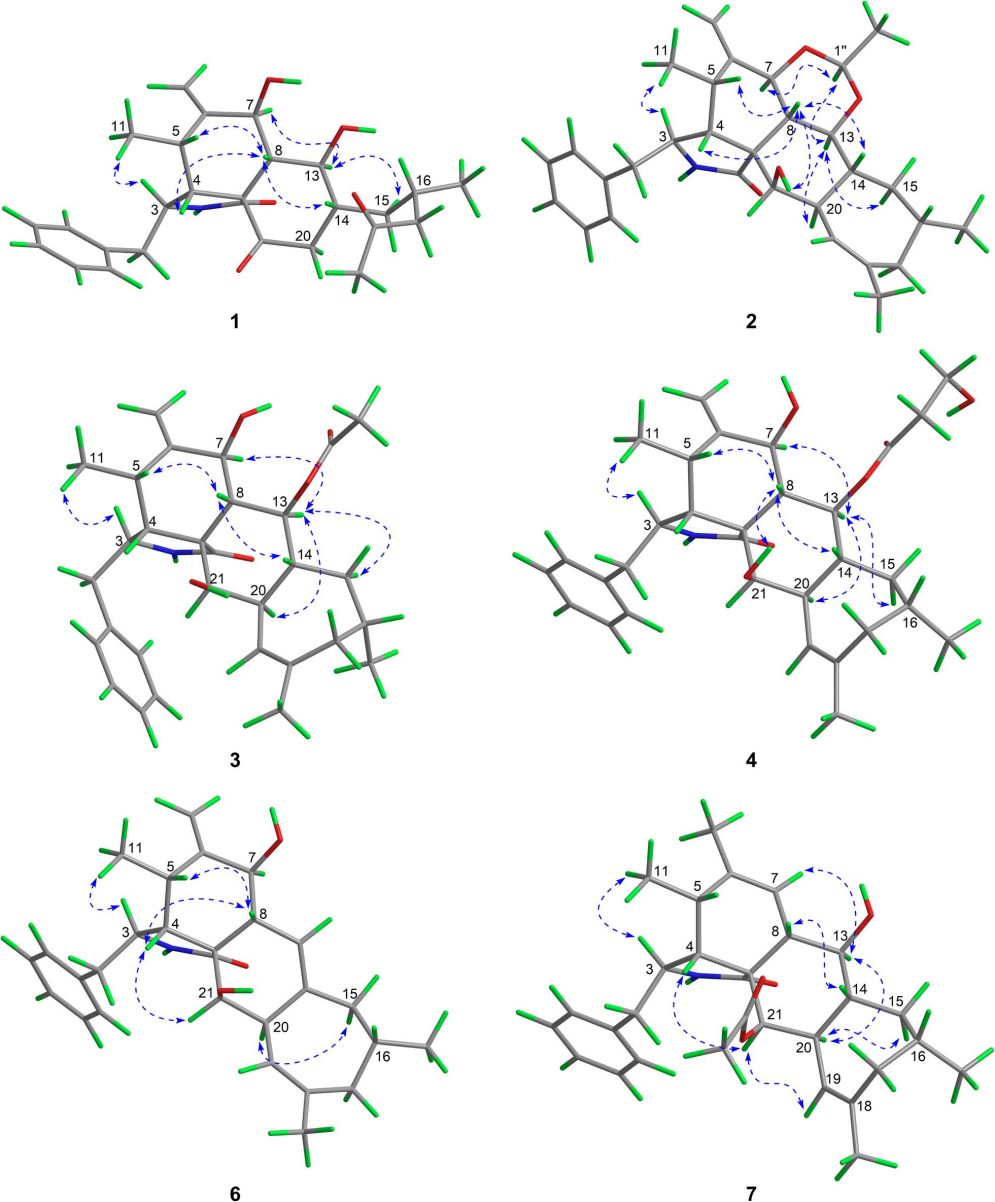
Key NOE correlations of **1**−**4** and **6**−**7**

**Fig. 4:**
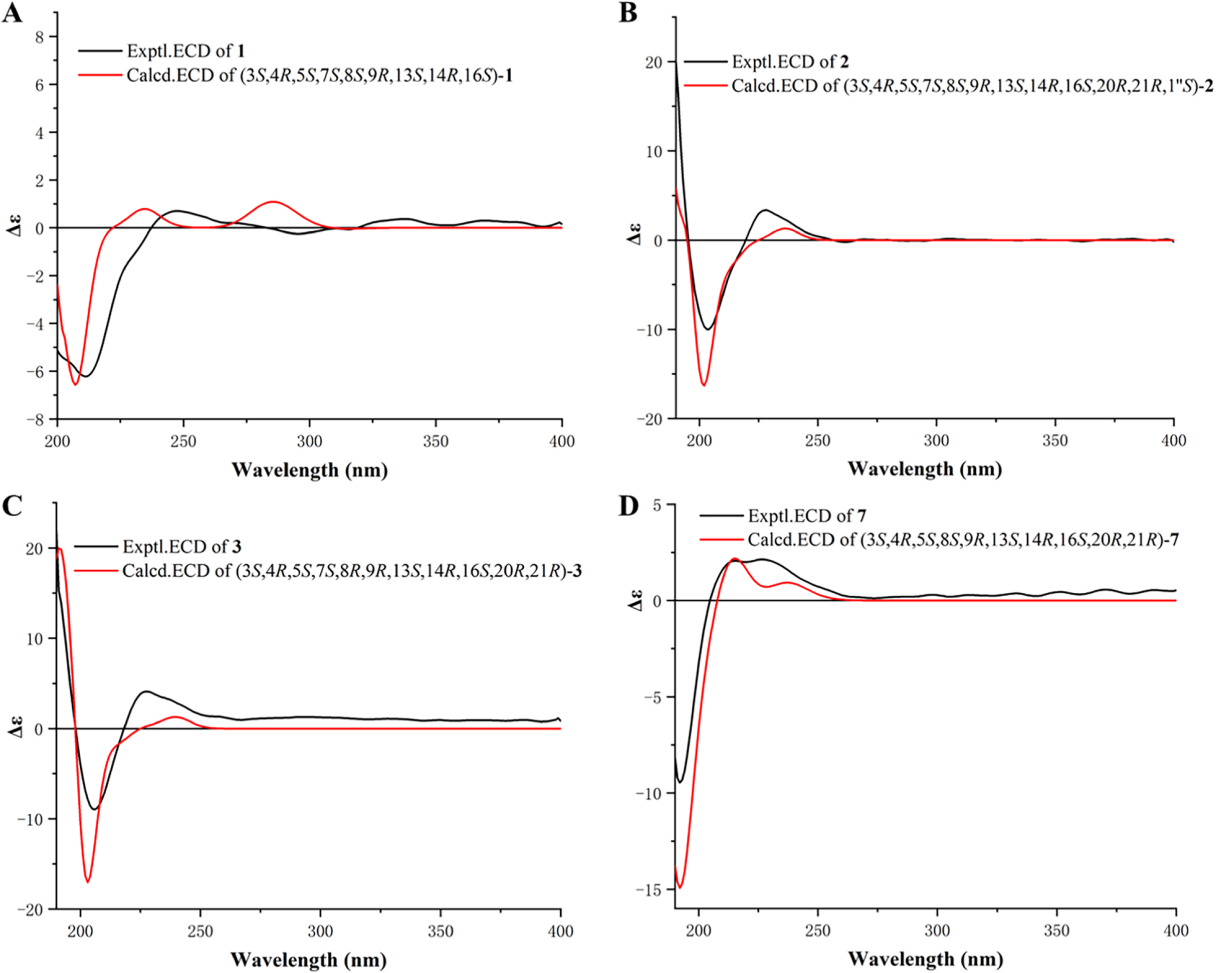
Experimental and calculated ECD curves (**A**−**C**) of **1**−**3** and **7** (**D**)

The relative configurations of C-3, C-4, C-5, C-7, C-8, and C-9 in **1** were established to be the same as those of phomopsischalin B (**8**) [6] by comparison of their 1D NMR and NOESY data. In particular, H-8 exhibited a typical triple peak with a coupling constant of 9.6 Hz, indicating that H-7 and H-8 together with H-13 and H-8 were both in *trans* configuration. Therefore, it was arbitrarily designated that H-8 was *β*-oriented, while H-7 and H-13 were *α*-oriented. The NOE interactions of H-5/H-8, H-4/H-8, and H-14/H-8 indicated that all these protons were *β*-orientation (Fig. 3), and the observed NOE correlation of H-3/H_3_-11 suggested the *α*-orientation of H-3. According to the biosynthetic pathway, the relative configuration of H-16 was determined to be the same as the *β*-orientation of **8**. The absolute configuration of **1** was assigned by comparison of its experimental ECD spectrum with those calculated for two presumable isomers based on time-dependent density functional theory (TDDFT) (Fig. 4A).

**Scheme 1.**
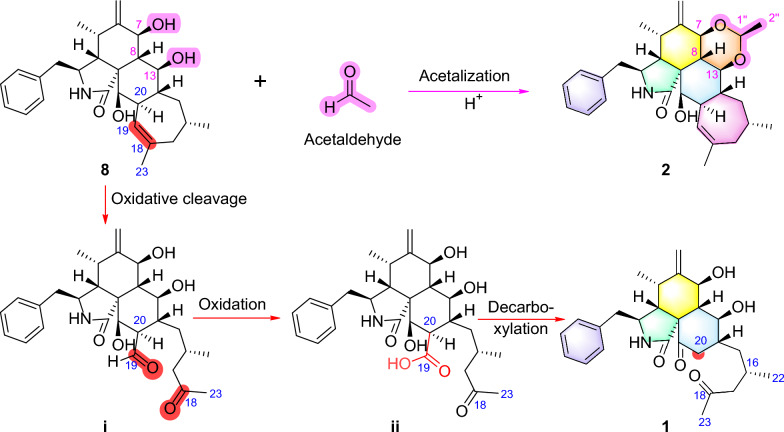
Proposed Biosynthetic Pathways for **1** and **2**

Therefore, the structure of compound **1** was identified as an unprecedented 19,20-*seco*-19-*nor*-5/6/6/7-fused tetracyclic cytochalasin scaffold. It represents the first example of 5/6/6-fused tricyclic cytochalasin featuring a 2-methyl-4-oxopentyl side chain, which is likely derived from phomopsischalin B (**8**) [6] by oxidative ring-opening and decarboxylation (Scheme 1).

Harziachalasin B (**2**) exhibited a molecular formular of C_30_H_39_NO_4_ with 12 degrees of unsaturation based on the HR-ESI-MS data (*m/z* 500.2776 [M + Na]^+^, calcd. 500.2771). Its spectroscopic data (Table 1) bore a resemblance to those of **8**, except for the presence of an additional ethane-1,1-dioxy group [*δ*_H_ 1.36 (3H, d, *J* = 5.1 Hz) and 4.82 (1H, q, *J* = 5.1 Hz); *δ*_C_ 21.3 (CH_3_) and 99.4 (CH)], along with the changes in the chemical shifts of the oxygenated methines C-7 (*δ*_C_ 75.4) and C-13 (*δ*_C_ 78.1) and their adjacent carbons in **2**. This information suggested that the newly emerged C2 unit may be associated with C-7 or/and C-13. Combined with an additional degree of unsaturation (12 degrees of unsaturation for **2**; 11 degrees of unsaturation for **8**) and the HMBC correlations from H-7 and H-13 to C-1′′ (*δ*_C_ 99.4) and from H-1′′ to C-7 and C-13, it can be determined that the newly introduced ethane-1,1-dioxy group formed a new 1,3-dioxane ring with the B/C rings. Therefore, a 5/6/6/6/7-fused pentacyclic ring system skeleton for **2** was constructed. The relative configuration of the newly formed chiral center (C-1′′) was determined as S* by the observed NOE correlations of H-7/H-1′′ and H-13/H-1′′. In addition, no correlations between H-16 and other hydrogen atoms were observed in the NOESY spectrum. This absence of signals may be attributed to significant overlap in the high-field region, which could obscure the relevant cross-peaks. Ultimately, the configuration of H-16 was unambiguously assigned as 16*S* through NMR chemical shift calculations combined with DP4 + probability analysis. Between the two possible stereoisomers, 16*S*-**2a** and 16*R*-**2b**, only the 16*S* configuration yielded a DP4 + probability greater than 100.00% (Figure S2), thereby confirming its absolute configuration. The relative configurations of other chiral centers were confirmed to be the same as **8** by 1D NMR and NOE correlations (Fig. 3) analysis. Considering the strong resemblance between the experimental and calculated ECD curves (Fig. 4B), the absolute configuration of **2** was thereby assigned to be 3*S*,4*R*,5*S*,7*S*,8*S*,9*R*,13*S*,14*R*,16*S*,20*R*,21*R*,1′′*S*. Thus, the structure of **2** was determined as shown, which represents the first example of 5/6/6/6/7-fused pentacyclic ring system skeleton for cytochalasins. The plausible biosynthetic pathway of **2** may be the condensation of the 7-OH and 13-OH on compound **8** with acetaldehyde (Scheme 1).

Harziachalasin C (**3**) possessed the molecular formular C_30_H_39_NO_5_ with 12 degrees of unsaturation, by HR-ESI–MS ([M + Na]^+^ *m/z* 516.2712, calcd. 516.2720). Interpretation of NMR data (Table 2) established the carbocyclic skeleton of **3** being identical with that of **8**, except for the presence of an additional acetoxy group [*δ*_H_ 2.07 (3H, s); *δ*_C_ 21.9 (CH_3_) and 171.8 (C)] in **3**. By analysis of the HMBC correlations (Fig. 2) from H-13 [*δ*_H_ 5.80 (1H, t, *J* = 10.2 Hz)] to C-1′′ (*δ*_C_ 171.8), it confirmed the attachment of the acetoxy group at C-13. The relative configuration of **3** was consistent with that of **8** according to comparable observations in the 1D NMR and NOESY experiments (Fig. 3). The identical experimental and calculated ECD curves (Fig. 4C) ascertained its absolute configuration as shown.

**Table 2:** ^1^H (400 MHz) and ^13^C (100 MHz) NMR Data of Compounds **3** and **4** in CDCl_3_ (*δ* in ppm)

Position	3		4	
	*δ*_H_, multi. (*J* in Hz)	*δ*_C_, type	*δ*_H_, multi. (*J* in Hz)	*δ*_C_, type
1		176.3, C		176.3, C
2-NH	5.41, br s		5.48, s	
3	3.31, overlapped	53.2, CH	3.30, m	53.3, CH
4	2.71, overlapped	47.3, CH	2.70, overlapped	47.2, CH
5	2.76, overlapped	31.7, CH	2.75, m	31.7, CH
6		151.0, C		151.2, C
7	4.23, d (9.0)	73.8, CH	4.20, d (9.3)	73.7, CH
8	2.36, m	40.8, CH	2.36, m	40.5, CH
9		52.2, C		52.7, C
10	a 2.80, overlapped; b 2.68, overlapped	44.9, CH_2_	a 2.80, m; b 2.66, m	44.8, CH_2_
11	0.96, d (7.0)	12.7, CH_3_	0.95, d (6.7)	12.7, CH_3_
12	a 5.06, s; b 4.88, s	112.2, CH_2_	a 5.05, s; b 4.88, s	112.3, CH_2_
13	5.80, t (10.2)	76.0, CH	5.82, t (10.1)	77.3, CH
14	1.74, overlapped	38.8, CH	1.75, overlapped	38.6, CH
15	*α* 1.16, dt (13.5, 10.9); *β* 1.86, m	41.4, CH_2_	*α* 1.15, m; *β* 1.89, m	41.5, CH_2_
16	1.55, overlapped	32.4, CH	1.54, m	32.4, CH
17	a 2.03, m; b 1.93, m	41.7, CH_2_	2.03, m	41.9, CH_2_
18		141.1, C		141.3, C
19	5.16, m	124.9, CH	5.15, m	124.8, CH
20	2.89, d (9.9)	42.9, CH	2.87, m	42.8, CH
21	3.30, m	75.4, CH	3.28, m	75.3, CH
22	0.91, d (6.7)	24.7, CH_3_	0.93, d (6.8)	24.7, CH_3_
23	1.78, s	28.1. CH_3_	1.77, s	28.1, CH_3_
1′		137.6, C		137.5, C
2′/6′	7.15, d (6.8)	129.5, CH	7.14, d (6.9)	129.5, CH
3′/5′	7.32, t (7.2)	128.9, CH	7.31, t (7.2)	128.9, CH
4′	7.26, t (7.1)	127.2, CH	7.26, t (7.2)	127.1, CH
1′′		171.8, C		170.0, C
2′′	2.07, s	21.9, CH_3_	2.98, m	32.0, CH_2_
3′′			4.66, t (6.3)	70.2, CH_2_
21-OH	1.84, br s		1.90, br s	

**Fig. 5:**
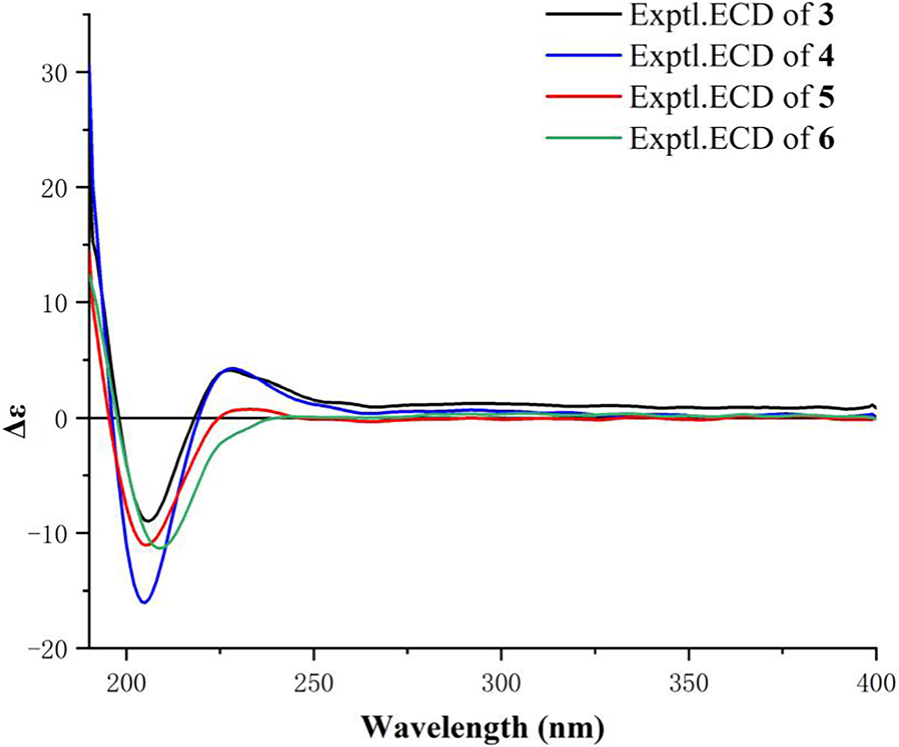
Experimental ECD curves of **3**−**6**

Harziachalasin D (**4**) possessed the molecular formula of C_31_H_41_NO_6_ as established from the ion peak at *m/z* 524.2988 [M + H]^+^ (calcd. 524.3007) in its HR-ESI-MS spectrum. Comparison of the spectroscopic data (Table 2) implied that **4** possessed a 3-hydroxypropionyloxy group [*δ*_H_ 2.98 (2H, m) and 4.66 (2H, t, *J* = 6.3 Hz); *δ*_C_ 32.0 (CH_2_), 70.2 (CH_2_), and 170.0 (C)] instead of the acetoxy in **3**, which could be further confirmed by the ^1^H–^1^H COSY correlations of H-2′′/H-3′′ and the HMBC correlations from H-2′′ and H-3′′ to C-1′′ (Fig. 2). The location of the 3-hydroxypropionyloxy group at C-13 was supported by the HMBC correlation from H-13 to C-1′′. The analysis of the NOESY correlations (Figure S1) and the experimental ECD spectrum (Fig. 5) confirmed that the stereochemistry of **4** was identical to that of **3**.

Harziachalasin E (**5**) displayed a sodium adduct HR-ESI-MS ion at [M + Na]^+^ *m/z* 488.2764 (calcd. 488.2771), indicating the molecular formula C_29_H_39_NO_4_ with 11 degrees of unsaturation. Its spectroscopic data (Table 3) bore a close resemblance to those of **3**, except for the appearance of a methoxy (*δ*_H_ 3.55; *δ*_C_ 60.1) rather than the acetoxy in **3**. The location of the methoxy at C-13 was verified by the important HMBC correlation from the protons of the methoxy to the oxygenated methine [*δ*_C_ 85.6 (CH, C-13)] (Fig. 2). By analyzing its 1D NMR data and NOE correlations, it can be determined that the relative configurations of compounds **5** and **3** were the same (Figure S1). The similar ECD curves of compounds **3**−**5** (Fig. 5) suggested these compounds shared the same absolute configurations.

**Table 3:** ^1^H (400 MHz) and ^13^C (100 MHz) NMR Data of Compounds **5**−**7** in CDCl_3_ (*δ* in ppm)

Position	5		6		7	
	*δ*_H_, multi. (*J* in Hz)	*δ*_C_, type	*δ*_H_, multi. (*J* in Hz)	*δ*_C_, type	*δ*_H_, multi. (*J* in Hz)	*δ*_C_, type
1		176.8, C		176.2, C		176.1, C
2-NH	5.39, br s		5.38, br s		5.39, br s	
3	3.31, m	53.1, CH	3.28, m	52.7, CH	3.24, m	55.1, CH
4	2.71, dd (5.7, 2.5)	47.3, CH	2.75, dd (6.2, 1.9)	47.2, CH	2.15, m	51.2, CH
5	2.80, overlapped	32.0, CH	2.90, t (6.4)	32.1, CH	2.32, m	35.5, CH
6		148.3, C		151.4, C		139.2, C
7	4.22, d (9.0)	73.7, CH	4.09, d (11.2)	72.0, CH	5.73, br s	125.2, CH
8	2.29, t (9.6)	41.9, CH	3.02, br d (10.6)	37.2, CH	2.38, overlapped	42.0, CH
9		51.8, C		50.4, C		55.8, C
10	a 2.81, dd (13.4, 4.9); b 2.63, dd (13.4, 8.8)	45.0, CH_2_	a 2.77, overlapped; b 2.69, m	45.3, CH_2_	a 2.81, dd (13.5, 5.0); b 2.65, dd (13.4, 9.5)	45.8, CH_2_
11	0.99, d (6.8)	12.9, CH_3_	0.97, d (7.1)	12.8, CH_3_	1.02, d (7.3)	13.6, CH_3_
12	a 5.16, s b 4.98, s	113.2, CH_2_	a 5.16, s; b 5.00, s	113.7, CH_2_	1.71, s	20.1, CH_3_
13	4.19, t (10.2)	85.6, CH	5.64, br s	118.7, CH	4.19, t (9.9)	72.8, CH
14	1.78, overlapped	40.6, CH		136.8, C	1.53, m	43.0, CH
15	*α* 1.13, dt (13.2, 10.7); *β* 2.23, m	40.0, CH_2_	*α* 1.76, dd (12.5, 10.2); *β* 2.43, dd (12.5, 7.3)	40.6, CH_2_	*α* 1.26, m; *β* 2.38, overlapped	43.3, CH_2_
16	1.64, m	32.6, CH	2.03, m	34.4, CH	1.58, m	32.4, CH
17	a 2.13, dd (15.1, 2.8); b 1.89, dd (15.1, 8.8)	41.4, CH_2_	1.51, dd (13.8, 2.1)	36.2, CH_2_	1.97, m	41.9, CH_2_
18		140.7, C		140.0, C		139.4, C
19	5.09, m	124.9, CH	5.23, d (5.9)	121.1, CH	5.00, m	124.9, CH
20	2.84, overlapped	44.1, CH	3.42, m	46.8, CH	3.16, m	41.8, CH
21	3.23, t (2.3)	75.4, CH	3.54, d (3.1)	72.0, CH	5.33, d (2.8)	76.5, CH
22	0.97, d (6.8)	24.7, CH_3_	0.95, d (7.2)	21.6, CH_3_	0.96, d (6.7)	24.9, CH_3_
23	1.77, s	28.3, CH_3_	1.85, s	28.4, CH_3_	1.71, s	27.7, CH_3_
1′		137.7, C		137.8, C		137.9, C
2′/6′	7.15, d (6.9)	129.5, CH	7.16, d (6.9)	129.4, CH	7.15, d (6.9)	129.2, CH
3′/5′	7.32, t (7.2)	128.9, CH	7.31, t (7.3)	128.9, CH	7.30, t (7.3)	129.0, CH
4′	7.26, t (7.2)	127.1, CH	7.23, t (7.3)	127.1, CH	7.23, t (7.4)	127.0, CH
13-OCH_3_	3.55, s	60.1, CH_3_				
7-OH	4.14, s					
21-OH	1.84, d (3.3)					
21-OAc					2.18, s	171.4, C 21.3, CH_3_

Harziachalasin F (**6**) had the molecular formula C_28_H_35_NO_3_ as derived from HR-ESI-MS analysis (*m/z* 434.2681 [M + H]^+^, calcd. 434.2690). Its NMR data (Table 3) for **6** were also very similar to those of **8**, with the apparent differences being due to the presence of an additional trisubstituted double bond [*δ*_H_ 5.64; *δ*_C_ 118.7 (CH) and 136.8 (C)] instead of two methines including an oxygenated one, which suggested that **6** was a dehydration product of **8**. Detailed analysis of their 2D NMR data confirmed the above deduction, especially by the observation of HMBC correlations from H-7 to C-13 and from H_2_-15 to C-13 (Fig. 2). The relative configurations of C-3, C-4, C-5, C-7, C-8, C-9, C-16, and C-20 in **6** were determined to be the same as **8** by comparing their 1D NMR data and the observed NOE correlations (Fig. 3). The experimental ECD spectrum (Fig. 5) showed the absolute configuration of **6** was identical to that of **3**−**5**.

Harziachalasin G (**7**) possessed a molecular formula of C_30_H_39_NO_4_, as determined by the HR-ESI-MS ion at *m/z* 478.2943 [M + H]^+^ (calcd. for 478.2952). The 1D NMR data (Table 3) of **7** exhibited similarities to those of **3**, except for the disappearance of a terminal methylene and an oxygenated methine in **3** instead of the presence of a singlet methyl and an olefinic methine, which indicated that the Δ^6(12)^ double bond in **3** was migrated to Δ^6^ in **7**. This hypothesis was further confirmed by the ^1^H−^1^H COSY of H-8/H-7 and the HMBC correlations from H_3_-12 to C-5, C-6, and C-7. However, the acetoxy was located at C-21 instead of at C-13, which was supported by the HMBC correlation from H-21 to the ester carbonyl (*δ*_C_ 171.4) (Fig. 2). The relative configuration of **7** was assigned as shown by analysis of the NOESY spectrum (Fig. 3). Finally, the (3*S*,4*R*,5*S*,8*S*,9*R*,13*S*,14*R*,16*S*,20*R*,21*R*) absolute configuration of **7** was elucidated by the ECD calculation (Fig. 4D).

Three known analogues, phomopsischalin B (**8**) [6], phomopsischalin A (**9**) [6], and ueckerchalasin A (**10**) [38] were identified by comparison of their experimental spectroscopic data with those reported.

### Screening for HIV latency reversing agents (LRAs)

**Fig. 6:**
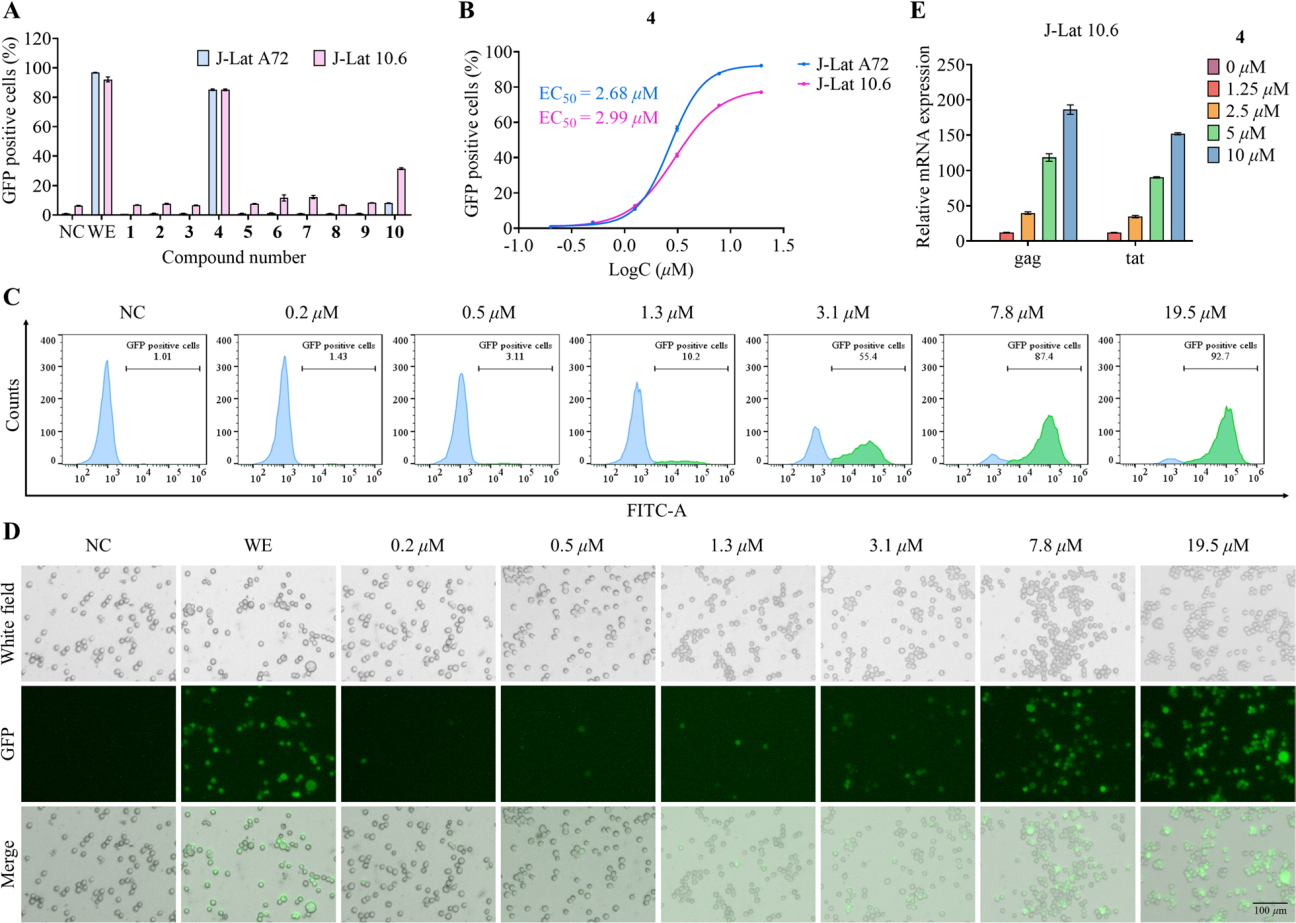
Evaluation of HIV latency reversing agent activity of CYTs **1**−**10**. **A** Flow cytometry analysis for screening HIV latency reversing agents. **B** EC_50_ curves of **4** in J-Lat A72 and J-Lat 10.6 cell lines. **C** Representative flow cytometry scatter plots demonstrating the HIV latency reversal effect of **4** at various concentrations. **D** Representative fluorescence microscopy images illustrating the HIV latency reversal effect of **4** at various concentrations. **E** Relative expression levels of HIV *gag* and *tat* mRNAs in J-Lat 10.6 cells treated with **4** at different doses for 24 h. Negative control (NC): Cells were treated with vehicle (DMSO). Wikstroelide E (WE): Positive control, cells were treated with 50 nM WE

The ten isolated natural CYTs (**1**−**10**) were evaluated for their ability to reverse HIV latency using two well-established cell models: J-Lat A72 and J-Lat 10.6 [39], and wikstroelide E served as the reference compound in the experimental group [40]. Following a 24 h incubation period with each compound at a concentration of 10 μM, flow cytometry analysis demonstrated that **4** exhibited the most potent latency-reversing activity in both cell lines (Fig. 6A). Dose–response studies revealed that **4** displayed significant potency, with half-maximal effective concentration (EC_50_) of 2.68 μM in J-Lat A72 cells and 2.99 μM in J-Lat 10.6 cells (Fig. 6B). Notably, the compound exhibited no cytotoxic effects on normal cells, as confirmed by cell viability assays (Fig. S3), with an IC_50_ value exceeding 40 μM in HEK-293 T cells. Representative flow cytometry scatter plots (Fig. 6C) and corresponding fluorescence microscopy images (Fig. 6D) confirmed the dose-dependent nature of HIV reactivation by **4**. Beyond GFP expression, quantitative RT-qPCR analysis demonstrated that **4** also induced dose-dependent upregulation of key HIV transcripts, including *gag* and *tat* mRNA (Fig. 6E). This multi-parameter validation strengthens the conclusion that compound **4** represents a promising lead for HIV latency reversal.

### Compound 4 reverses HIV latency via activating the NF-*κ*B pathway, and preliminary RNA-seq analysis suggests potential dual antiviral-antitumor effects

**Fig. 7:**
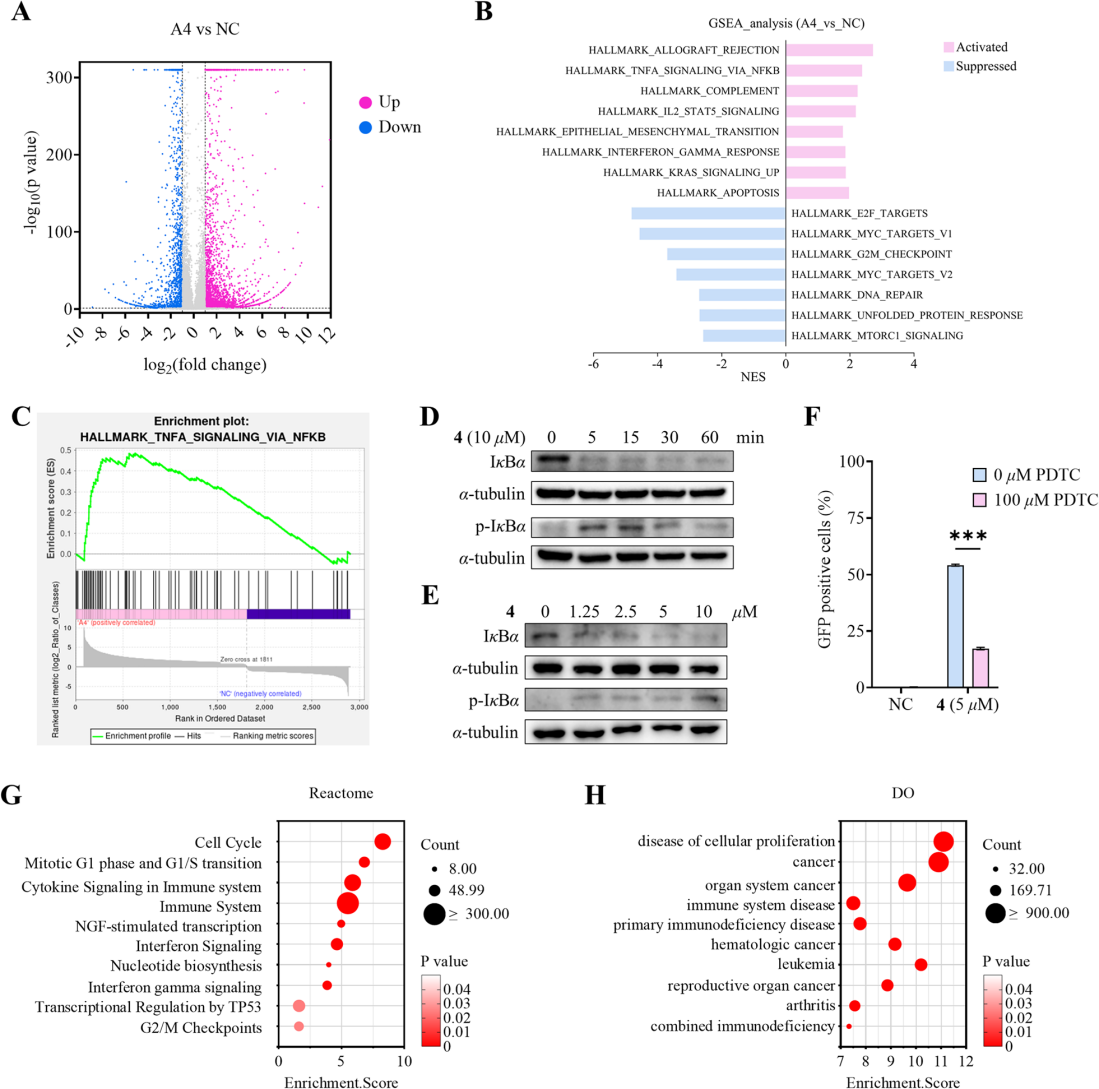
Compound **4** reprograms transcriptomes in the HIV latent cell model with experimental validation of NF-*κ*B pathway activation. **A** Volcano plot shows significantly different expressed genes in J-Lat 10.6 cells treated with or without 10 μM of **4** for 24 h. **B** GSEA enrichment result of the Hallmark gene set. **C** Positive enrichment of the Hallmark TNF-*α* signaling via NF-*κ*B gene set in compound **4**-treated cells. **D** I*κ*B*α* degradation and phosphorylation induced by 10 μM compound **4**. **E** Dose-dependent I*κ*B*α* degradation and phosphorylation induced by **4** after 15-min treatment. **F** PDTC attenuates the HIV latency reversal effect of **4**. **G** Bubble plot depicting enriched Reactome pathways results. **H** Results from Disease Ontology (DO) enrichment analysis were demonstrated by bubble plot

To elucidate the mechanism of compound **4** as an HIV LRA, we conducted RNA sequencing in the J-Lat 10.6 cell line following 24 h treatment with either DMSO (negative control, NC) or 10 μM compound **4** (sample name: A4). Comparative analysis revealed 3464 significantly upregulated genes‌ and ‌1750 markedly downregulated genes (Fig. 6A). Gene set enrichment analysis (GSEA) further demonstrated that compound **4** strongly activates the NF-*κ*B signaling pathway (Fig. 7B and C).

Previous studies have established that latent HIV-1 reactivation is mediated by the NF-*κ*B signaling pathway [41–43]. To systematically evaluate whether the latency-reversing activity of compound **4** operates through this canonical pathway, we conducted a series of mechanistic investigations focusing on key regulatory components of NF-*κ*B activation. Western blot analysis was employed to quantify the protein levels of I*κ*B*α* and its phosphorylated form (p-I*κ*B*α*), both are critical regulators in the canonical NF-*κ*B activation cascade. Time-course (0−60 min) and dose–response (0−10 μM) experiments were performed to characterize the dynamic effects of **4** on these molecular targets. As shown in Fig. 7D and E, compound **4** promoted the phosphorylation of I*κ*B*α* in a concentration-dependent manner, as reflected by the initial increase in p-I*κ*B*α* levels. This was followed by a subsequent degradation of p-I*κ*B*α*, resulting in an overall reduction in both p-I*κ*B*α* and I*κ*B*α* protein levels over time. To confirm the pathway specificity, we employed ammonium pyrrolidine dithiocarbamate (PDTC), a well-characterized inhibitor of NF-*κ*B signaling pathway. Pretreatment with 100 μM PDTC significantly attenuated the latency-reversing capacity of compound **4** by approximately 69% (Fig. 7F), providing strong pharmacological evidence for NF-*κ*B pathway involvement. Collectively, these data establish that compound **4** mediates HIV-1 latency reversal primarily through activating canonical NF-*κ*B pathway. This mechanistic understanding positions **4** as a valuable chemical tool for further investigation of NF-*κ*B-dependent latency reversal strategies.

Our integrated RNA-seq and experimental validation demonstrate that compound **4** exerts its HIV latency-reversing activity by NF-*κ*B pathway activation, a mechanism consistent with established HIV-1 reactivation models. Transcriptomic profiling further revealed its modulation of cancer-associated pathways, including cytokine signaling, cell cycle, immune responses, and DNA repair (Fig. 7B and G), consistent with the anticancer effects of related CYTs (e.g., cell cycle arrest) [44–46]. These multifaceted effects collectively explain its association with immune-related diseases and multiple cancers (Fig. 7H). This dual functionality addresses a critical unmet need: HIV patients exhibit higher cancer incidence, with cancer accounting for 20% of deaths despite viral suppression [47–49]. We thus propose a novel therapeutic strategy − NF-*κ*B-involved HIV reservoir clearance combined with oncogenic pathway modulation − which is especially relevant given HIV patients’ elevated cancer risk. To assess compound **4**’s broader therapeutic potential, in vitro and in vivo anti-tumor efficacy studies will be conducted to validate these findings.

## Conclusions

Chemical investigation of the endophytic fungus *T. harzianum* MLJ-4 isolated from the medicinal plant *A. curassavica* led to the discovery of ten CYTs (**1**−**10**) from its rice fermentation extract, including seven novel metabolites designated harziachalasins A−G (**1**−**7**) characterized through comprehensive spectroscopic analysis. This study unveiled two unprecedented structural frameworks in CYTs chemistry: compound **1** represents the first 19,20-*seco*-19-*nor*-5/6/6-fused tricyclic scaffold with a distinctive 2-methyl-4-oxopentyl side chain at C-14, establishing a novel subclass, while compound **2** features an exceptional 1,3-dioxane-incorporated E-ring forming the inaugural 5/6/6/6/7-fused pentacyclic system in this class. These findings significantly expand the structural diversity of the rare 5/6/6/7-tetracyclic CYTs, with only seven prior analogues reported including ueckerchalasin C [38], diaporchalasin D [50], diaporchalasin E [50], and curtachalasin P [51] together with compounds **8**−**10** isolated by this work. Biological evaluation demonstrated that compound **4** effectively reverses HIV latency (EC_50_ = 2.68 μM in J-Lat A72; 2.99 μM in J-Lat 10.6 cells) by activating the NF-*κ*B pathway. This mechanism was confirmed through three lines of evidence: (1) RNA-seq analysis showing NF-*κ*B pathway upregulation, (2) I*κ*B*α* degradation/phosphorylation, and (3) significant attenuation of activity by the NF-*κ*B inhibitor PDTC. Notably, RNA-seq also revealed an additional therapeutic benefit: compound **4** modulated cancer-related pathways, potentially reducing the elevated cancer risk observed in HIV patients. The structural innovations coupled with mechanistically elucidated bioactivity highlight both the metabolic versatility of *Asclepias*-associated endophytes and the potential of these derivatives as valuable leads for combined HIV/cancer therapy, positioning them as promising candidates for further structure–activity optimization and in vivo efficacy studies.

## Experimental section

### General experimental procedures

MCP 200 modular circular polarimeter from Anton Paar was used to determine the optical rotations. An Applied Photophysics Chirascan spectrometer was used to measure ECD and UV data. A PerkinElmer Spectrum Two FTIR spectrometer with a UATR accessory was applied to collect IR spectra. 1D and 2D NMR spectra were recorded using Bruker Avance III 400 and Bruker Ascend TM 500 spectrometers. A Micromass Q-TOF spectrometer from Waters was used to receive HR-ESI–MS data. Column chromatography (CC) fractionations mainly relied silica gel (100–200, 200–300, and 300–400 mesh, Qingdao Haiyang Chemical Co., Ltd.), reversed-phase C_18_ (Rp-C_18_) silica gel (12 nm, S-50 μm, YMC Co., Ltd.), and Sephadex LH-20 gel (Amersham Biosciences). High-performance liquid chromatography (HPLC) separations were conducted on a Shimadzu LC-20 AT with an SPD-M20A PDA detector (Kyoto, Japan) using a YMC-pack ODS-A column (10 × 250 mm, S-5 μm) and a NanoChrom ChromCoreTM 5–120 C_18_ column (250 × 10 mm, 5 µm). Chemical solvents were of analytical grade (Guangzhou Chemical Reagents Company, Ltd.) while acetonitrile (MeCN) was of HPLC grade (Grace Chemical Technology Co., Ltd.).

### Fungal materials

The strain *T. harzianum* MLJ-4 was isolated from the plant *A. curassavica*, which was collected from South China Botanical Garden of Guangzhou city. The strain was identified by sequence analysis of *r*DNA ITS (internal transcribed spacer) region. The sequence of ITS region of the fungus MLJ-4 has been submitted to GenBank (Accession No. MZ930375.1). The strain has been deposited at the School of Pharmaceutical Sciences, Sun Yat-sen University.

### Fermentation, extraction, and isolation

The fungus *T. harzianum* MLJ-4 was maintained on potato dextrose agar (PDA) medium at 28 °C for 5 d, and then three pieces (0.5 × 0.5 cm^2^) of mycelial agar plugs were inoculated into Erlenmeyer flasks, each containing 250 mL potato dextrose broth (PDB). After 4 days of incubation at 28 °C on a rotary shaker at 120 r/m, seed cultures were aseptically transferred into the rice solid medium (each flask contained 100.0 g of rice, 100 mL of natural filtered water). The 200 flasks were then kept at 28 °C for 30 days.

All fermentation products of the fungus MLJ-4 were exhaustively extracted with EtOAc (EA) for three times (each 20 L) at room temperature to afford 338.0 g of crude extract. Subsequently, the crude extract was subjected to silica gel (100−200 mesh) CC and eluted with mixed solvent of petroleum ether (PE) and EA to obtain 11 fractions (Frs. A−K).

Fr. E (4.6 g) was separated to CC on Rp-C_18_ silica gel and eluted with MeOH/H_2_O (40% → 100%) to led to seven sub-fractions (Frs. E1−E7). Fr. E5 (200.0 mg) was further purified by silica gel (300−400 mesh) CC (CH_2_Cl_2_/MeOH, 100:1 → 30:1) to obtain two sub-fractions (Frs. E5A and E5B). Fr. E5A (62.0 mg) was further purified by HPLC (MeCN/H_2_O, 50:50, 3 mL/min) to yield compounds **2** (3.2 mg, *t*_R_ 18.5 min) and **7** (3.5 mg, *t*_R_ 20.5 min). Fr. F (7.1 g) was subjected to CC on Rp-C_18_ silica gel and eluted with MeOH/H_2_O (40% → 100%) to give nine sub-fractions (Frs. F1−F9). Fr. F4 (419.0 mg) was further chromatographed by silica gel (300−400 mesh) CC (CH_2_Cl_2_/MeOH, 100:1 → 20:1) to obtain four sub-fractions (Fr. F4A−F4D). Fr. F4C (23.0 mg) was further purified by HPLC (MeCN/H_2_O, 65:35, 3 mL/min) to obtain compound **10** (5.6 mg, *t*_R_ 11.0 min). Fr. G (29.6 g) was separated to CC on Rp-C_18_ silica gel and eluted with MeOH/H_2_O (30% → 100%) to give 11 sub-fractions (Frs. G1−G11). Fr. G11 (973.0 mg) was further separated by silica gel (300−400 mesh) CC (CH_2_Cl_2_/MeOH, 100:1 → 20:1) to obtain two sub-fractions (Frs. G11A and G11B). Fr. G11A (307.0 mg) was further purified by silica gel (300−400 mesh) CC (PE/EtOAc, 5:1 → 1:2) to obtain five sub-fractions (Frs. G11A1−G11A5). Fr. G11A1 (70.0 mg) was further purified by silica gel (300−400 mesh) CC (CH_2_Cl_2_/MeOH, 200:1 → 20:1) to obtain three sub-fractions (Frs. G11A1A−G11A1C), and compound **5** (3.6 mg, *t*_R_ 14.0 min) was obtained from Fr. G11A1A (30.0 mg) by HPLC (MeCN/H_2_O, 50:50, 3 mL/min). Fr. G11A2 (40.0 mg) was further purified by HPLC (MeCN/H_2_O, 55:45, 3 mL/min) to afford compounds **3** (2.6 mg, *t*_R_ 14.0 min), **6** (2.4 mg, *t*_R_ 16.5 min), and **4** (2.9 mg, *t*_R_ 17.5 min). Fr. H (41.0 g) was chromatographed over silica gel (300−400 mesh) (CH_2_Cl_2_/MeOH, 100:1 → 20:1) to obtain two sub-fractions (Frs. H1 and H2). Fr. H1 (21.4 g) was subjected to CC on Rp-C_18_ silica gel and eluted with MeOH/H_2_O (30% → 100%) to give 11 sub-fractions (Frs. H1A−H1K). Fr. H1G (690.0 mg) was further purified by silica gel (300−400 mesh) CC (CH_2_Cl_2_/MeOH, 100:1 → 20:1) to obtain four sub-fractions (Frs. H1G1A−H1G4D). Fr. H1G2B (20.0 mg) was further purified by HPLC (MeCN/H_2_O, 52:48, 3 mL/min) to obtain compound **1** (4.5 mg, *t*_R_ 12.5 min). Fr. I (9.0 g) was subjected to CC on Rp-C_18_ silica gel and eluted with MeOH/H_2_O (30% → 100%) to give nine sub-fractions (Frs. I1−I9). Fr. I6 (1.3 g) was further purified by silica gel (300−400 mesh) CC (CH_2_Cl_2_/MeOH, 100:1 → 20:1) to obtain eight sub-fractions (Frs. I6A−I6H). Fr. I6F (179.0 mg) was further purified by HPLC (MeCN/H_2_O, 50:50, 3 mL/min) to give compounds **8** (4.9 mg, *t*_R_ 12.5 min) and **9** (3.2 mg, *t*_R_ 17.5 min).

### Spectroscopic data of compounds

#### Harziachalasin A (1)

White amorphous powder; [*α*]_D_^20^ + 20 (*c* 0.1, MeOH); UV (MeCN) *λ*_max_ (log *ε*) 190 (3.89) nm; ECD (*c* 0.22 × 10^−3^ M, MeCN) *λ*_max_ (Δ*ε*) 190 (+ 12.87) nm; IR (UATR) *ν*_max_ 3339, 2926, 1677, 1455, 1276, 1039, 750 cm^−1^; ^1^H and ^13^C NMR data see Table 1; HR-ESI–MS *m*/*z* 476.2384 [M + Na]^+^ (calcd. for C_27_H_35_NO_5_Na^+^, 476.2407).

#### Harziachalasin B (2)

White powder; [*α*]_D_^20^ + 38 (*c* 0.1, MeOH); UV (MeCN) *λ*_max_ (log *ε*) 190 (4.01) nm; ECD (*c* 0.21 × 10^−3^ M, MeCN) *λ*_max_ (Δ*ε*) 190 (+ 19.93), 228 (+ 3.35) nm; IR (UATR) *ν*_max_ 2925, 1680, 1275, 1100, 906, 749 cm^−1^; ^1^H and ^13^C NMR data see Table 1; HR-ESI–MS *m*/*z* 500.2776 [M + Na]^+^ (calcd. for C_30_H_39_NO_4_Na^+^, 500.2771).

#### Harziachalasin C (3)

White powder; [*α*]^20^_D_ + 24 (*c* 0.1, MeOH); UV (MeCN) *λ*_max_ (log *ε*) 190 (3.82) nm; ECD (*c* 0.20 × 10^−3^ M, MeCN) *λ*_max_ (Δ*ε*) 190 (+ 21.87), 228 (+ 4.10) nm; IR (UATR) *ν*_max_ 3355, 2924, 2854, 1679, 1455, 1373,1261, 1030, 908, 750 cm^−1^; ^1^H and ^13^C NMR data see Table 2; HR-ESI–MS *m*/*z* 516.2712 [M + Na]^+^ (calcd. for C_30_H_39_NO_5_Na^+^, 516.2720).

#### Harziachalasin D (4)

White powder; [*α*]_D_^20^ + 17 (*c* 0.1, MeOH); UV (MeCN) *λ*_max_ (log *ε*) 190 (3.88) nm; ECD (*c* 0.19 × 10^−3^ M, MeCN) *λ*_max_ (Δ*ε*) 190 (+ 16.23), 233 (+ 0.85) nm; IR (UATR) *ν*_max_ 3348, 2926, 1679, 1556, 1496, 1454, 1376, 1262, 1027, 911, 737 cm^−1^; ^1^H and ^13^C NMR data see Table 2; HR-ESI–MS *m*/*z* 524.2988 [M + H]^+^ (calcd. for C_31_H_42_NO_6_^+^, 524.3007).

#### Harziachalasin E (5)

White powder; [*α*]_D_^20^ + 67 (*c* 0.1, MeOH); UV (MeCN) *λ*_max_ (log *ε*) 190 (3.83) nm; ECD (*c* 0.21 × 10^−3^ M, MeCN) *λ*_max_ (Δ*ε*) 190 (+ 14.43), 233 (+ 0.76) nm; IR (UATR) *ν*_max_ 3337, 1639, 1275, 749 cm^−1^; ^1^H and ^13^C NMR data see Table 3; HR-ESI–MS *m*/*z* 488.2764 [M + Na]^+^ (calcd. for C_29_H_39_NO_4_Na^+^, 488.2771).

#### Harziachalasin F (6)

White powder; [*α*]_D_^20^ − 12 (*c* 0.1, MeOH); UV (MeCN) *λ*_max_ (log *ε*) 190 (3.76) nm; ECD (*c* 0.23 × 10^−3^ M, MeCN) *λ*_max_ (Δ*ε*) 190 (+ 12.35) nm; IR (UATR) *ν*_max_ 3347, 2925, 2854, 1663, 1458, 1275, 1023, 748 cm^−1^; ^1^H and ^13^C NMR data see Table 3; HR-ESI–MS *m*/*z* 434.2681 [M + H]^+^ (calcd. for C_28_H_36_NO_3_^+^, 434.2690).

#### Harziachalasin G (7)

White powder; [*α*]_D_^20^ + 4 (*c* 0.1, MeOH); UV (MeCN) *λ*_max_ (log *ε*) 190 (3.80) nm; ECD (*c* 0.21 × 10^−3^ M, MeCN) *λ*_max_ (Δ*ε*) 192 (− 9.46), 227 (+ 2.14) nm; IR (UATR) *ν*_max_ 3340, 2925, 1736, 1686, 1455, 1372, 1232, 1023, 750 cm^−1^; ^1^H and ^13^C NMR data see Table 3; HR-ESI–MS *m*/*z* 478.2943 [M + H]^+^ (calcd. for C_30_H_40_NO_4_^+^, 478.2952).

### ECD calculations

The details of the quantum chemical ECD calculation for compounds **1**−**3** and **7** are provided in Supplementary Information (see S2).

### Cell culture

The J-Lat A72 and J-Lat 10.6 cell lines (obtained from Professor Sun’ group at the School of Public Health, Shenzhen, Sun Yat-sen University), which serve as models for HIV latent infection, were maintained in RPMI 1640 medium (Gibco) containing 10% fetal bovine serum (Newzerum) and 1% Penicillin–Streptomycin (Gibco). HEK-293T cells were grown in DMEM medium supplemented with 10% fetal bovine serum and 1% Penicillin–Streptomycin. Cells were cultured at 37 °C in a humidified atmosphere with 5% CO_2_. Green fluorescent protein (GFP) expression was monitored as an indicator of HIV latency reversal following treatment.

### HIV latency reversing assay

J-Lat A72 and J-Lat 10.6 cells were seeded at a density of 4 × 10^5^ cells per well in 12-well plates and treated with test compounds for 24 h. Experimental controls included DMSO (negative control, NC) and 50 nM wikstroelide E (positive control, PC). Following treatment, cells were harvested by centrifugation (800 rpm, 5 min), washed with PBS, and analyzed for GFP expression. GFP-positive cells were visualized using an integrated cell imaging system (Thermo Fisher) and quantified by flow cytometry (Beckman).

### Cell viability assay

The viability of HEK-293T cells was assessed using a Cell Counting Kit-8 (CCK-8) assay. Briefly, cells were plated in 96-well plates at a density of 10,000 cells per well in six replicates. Following a 24 h incubation, the cells were treated with a range of compound concentrations for another 24 h. After compound removal, CCK-8 reagent (GlpBio) was added according to the manufacturer's protocol, and the absorbance at 450 nm was measured after 3 h of incubation at 37 °C.

### RT-qPCR analysis

J-Lat A72 and J-Lat 10.6 cells were treated with DMSO (negative control, NC) or compound **4** for 24 h. Total RNA was extracted using TRIeasy™ Total RNA Extraction Reagent (Yeasen) and reverse transcribed into cDNA using Hifair^®^ II 1st Strand cDNA Synthesis SuperMix for qPCR (Yeasen). Quantitative RT-PCR was performed using Hieff^®^ qPCR SYBR Green Master Mix (Yeasen) with the following primers sets:

GAPDH:

Forward: 5′-GTCTCCTCTGACTTCAACAGCG-3′.

Reverse: 5′-ACCACCCTGTTGCTGTAGCCAA-3′.

HIV *gag*:

Forward: 5′-AGCGTCAGTATTAAGCGGGG-3′.

Reverse: 5′-CAGGCCAGGATTAACTGCGA-3′.

HIV *tat*:

Forward: 5′-TAGACTAGAGCCCTGGAAGCA-3′.

Reverse: 5′-TCCGCTTCTTCCTGCCATAG-3′.

### RNA-seq analysis

J-Lat 10.6 cells were treated with either DMSO (negative control, NC) or 10 *μ*M compound 4 for 24 h. Total RNA was extracted and assessed for quality and concentration using a spectrophotometer or fluorometer. mRNA was purified from 1 *μ*g of total RNA using oligo(dT) selection and used to synthesize double-stranded cDNA by SuperScript Double-Stranded cDNA Synthesis Kit (Invitrogen) with random hexamer primers. The cDNA underwent end-repair, phosphorylation, and adapter ligation. After purification and PCR amplification, the library was sequenced on the NovaSeq X Plus platform (PE150). Differential gene expression analysis was performed using DEGseq. Significantly differentially expressed genes were defined as those with |log_2_FC|≥ 1 and p-value < 0.05. Raw and processed RNA-seq data were deposited in NCBI GEO database with an accession number GSE303365.

### Western blotting analysis

Cells treated with compound **4** under indicated conditions were collected by centrifugation (800 rpm, 25 ℃, 5 min) and lysed using IP buffer (Beyotime) containing phosphatase inhibitor (Selleck). Following centrifugation (12,000 rpm, 4 ℃, 20 min), the supernatant was mixed with 5 × dual color protein loading buffer and boiled at 100 ℃ for 5 min. Protein samples were separated by 10% sodium dodecyl sulfate–polyacrylamide gel electrophoresis (SDS-PAGE) and transferred to polyvinylidene fluoride (PVDF) membranes. The membranes were blocked with 5% nonfat milk and incubated with overnight at 4 ℃ with primary antibodies against *α*-Tubulin (Zenbio, 250009, dilution ratio 1:4000), I*κ*B*α* (Cell Signaling Technology, 44D4, dilution ratio 1:1000), and phospho-I*κ*B*α* (Cell Signaling Technology, 14D4, dilution ratio 1:1000). After overnight incubation, the membranes were treated with appropriate secondary antibodies for visualization.

## Supplementary Information

Supplementary Material 1. Figures related to the article; ECD calculation for **1**−**3**, and **7**; 1D and 2D NMR (in CDCl_3_), HR-ESI–MS, and IR spectra of **1**–**7**; ^1^H and ^13^C NMR (in CDCl_3_) spectra of **8**–**10** (PDF).

## Data Availability

All data generated and analyzed during this study are included in this published article and its Additional file 1.
