# Supplementary material for: Harziachalasins A–G, polycyclic-fused cytochalasins from the endophytic fungus Trichoderma harzianum MLJ-4 with HIV latency reversal activity
Source: Nat Prod Bioprospect. 2026 Jan 11;16(1):18. doi: 10.1007/s13659-025-00572-1 (PMC12790558; doi:10.1007/s13659-025-00572-1)

Supplementary Information

Harziachalasins A–G, polycyclic-fused cytochalasins from the endophytic fungus *Trichoderma harzianum* MLJ-4 with HIV latency reversal activity

Yan-Jiang Zhang[1†], Xian-Yuan Yang[1†], Yi-Fan Fu[1], Qiong Liao[2], Jia-Qian Chen[1], Xian-An Chen[1], Dong Huang[1], Tao Yuan[3], Xin Chen[4], Sheng Yin[1] and Gui-Hua Tang[1*]

[1]School of Pharmaceutical Sciences, Sun Yat-sen University, Guangzhou 510006, People's Republic of China

[2]Laboratory Animal Center, Sun Yat-sen University, Guangzhou 510006, People's Republic of China

[3]School of Health, Jiangxi Normal University, Nanchang 330022, People's Republic of China

[4]School of Life Science and Technology, Wuhan Polytechnic University, Wuhan 430023, People's Republic of China

*Correspondence:

Gui-Hua Tang

tanggh5@mail.sysu.edu.cn

[†]Yan-Jiang Zhang and Xian-Yuan Yang have contributed equally to this work.

Contents

S1. Figures··p5

Figure S1. Key NOE correlations of compound **5**

Figure S2. DP4+ analyses between experimental ^{13}C NMR chemical shifts of **2** and calculated ^{13}C NMR chemical shifts of 16*S*-**2a** (A) and 16*R*-**2b** (B).

Figure S3. The effect of compound **4** on HEK-293T cell viability.

S2. Experimental section··p6–22

S2.1. ECD calculations of compounds **1–3**, and **7**

Figure S4. B3LYP/6-31G(d) optimized lowest energy conformers for **1**

Figure S5. B3LYP/6-31G(d) optimized lowest energy conformers for **2**

Figure S6. B3LYP/6-31G(d) optimized lowest energy conformers for **3**

Figure S7. B3LYP/6-31G(d) optimized lowest energy conformers for **7**

Table S1. Energy (298.15 K) analysis for **1–3**, and **7**

Table S2. Calculated ECD data of **1** (**1C1** and **1C2**) in acetonitrile

Table S3. Calculated ECD data of **2** (**2C1–2C3**) in acetonitrile

Table S4. Calculated ECD data of **3** (**3C1 and 3C2**) in acetonitrile

Table S5. Calculated ECD data of **3** (**3C3 and 3C4**) in acetonitrile

Table S6. Calculated ECD data of **7** (**7C1–7C3**) in acetonitrile

Table S7. Calculated ECD data of **7** (**7C4–7C6**) in acetonitrile

S2.2. NMR calculation of **2**

Figure S8 Two candidate structures of **2**.

Figure S9 B3LYP/6-31G(d) optimized lowest energy conformers for 16*S*-**2a**.

Figure S10 B3LYP/6-31G(d) optimized lowest energy conformers for 16*R*-2**b**.

Table S8 Energy (298.15 K) analysis for **2**.

Table S9 Calculated ^{13}C-NMR chemical shifts for conformers of 16*S*-**2a**.

Table S10. Calculated ^{13}C-NMR chemical shifts for conformers of 16*R*-**2b**.

Figure S11 DP4+ evaluation of theoretical and experimental data of **2**.

S3. 1D and 2D NMR spectra of compounds **1–10**··································p23–46

**Spectrum S1.** ^{1}H NMR spectrum of **1** in $CDCl_3$	**Spectrum S2.** ^{13}C NMR and DEPT spectra of **1** in $CDCl_3$
**Spectrum S3.** HSQC spectrum of **1** in $CDCl_3$	**Spectrum S4.** $^{1}H–^{1}H$ COSY spectrum of **1** in $CDCl_3$
**Spectrum S5.** HMBC spectrum of **1** in $CDCl_3$	**Spectrum S6.** NOESY spectrum of **1** in $CDCl_3$
**Spectrum S7.** ^{1}H NMR spectrum of **2** in in $CDCl_3$	**Spectrum S8.** ^{13}C NMR and DEPT spectra of **2** in $CDCl_3$
**Spectrum S9.** HSQC spectrum of **2** in $CDCl_3$	**Spectrum S10.** $^{1}H–^{1}H$ COSY spectrum of **2** in $CDCl_3$
**Spectrum S11.** HMBC spectrum of **2** in $CDCl_3$	**Spectrum S12.** NOESY spectrum of **2** in $CDCl_3$
**Spectrum S13.** ^{1}H NMR spectrum of **3** in $CDCl_3$	**Spectrum S14.** ^{13}C NMR and DEPT spectra of **3** in $CDCl_3$
**Spectrum S15.** HSQC spectrum of **3** in $CDCl_3$	**Spectrum S16.** $^{1}H–^{1}H$ COSY spectrum of **3** in $CDCl_3$
**Spectrum S17.** HMBC spectrum of **3** in $CDCl_3$	**Spectrum S18.** NOESY spectrum of **3** in $CDCl_3$
**Spectrum S19.** ^{1}H NMR spectrum of **4** in $CDCl_3$	**Spectrum S20.** ^{13}C NMR and DEPT spectra of **4** in $CDCl_3$
**Spectrum S21.** HSQC spectrum of **4** in $CDCl_3$	**Spectrum S22.** $^{1}H–^{1}H$ COSY spectrum of **4** in $CDCl_3$
**Spectrum S23.** HMBC spectrum of **4** in $CDCl_3$	**Spectrum S24.** NOESY spectrum of **4** in $CDCl_3$
**Spectrum S25.** ^{1}H NMR spectrum of **5** in $CDCl_3$	**Spectrum S26.** ^{13}C NMR and DEPT spectra of **5** in $CDCl_3$
**Spectrum S27.** HSQC spectrum of **5** in $CDCl_3$	**Spectrum S28.** $^{1}H–^{1}H$ COSY spectrum of **5** in $CDCl_3$
**Spectrum S29.** HMBC spectrum of **5** in $CDCl_3$	**Spectrum S30.** NOESY spectrum of **5** in $CDCl_3$
**Spectrum S31.** ^{1}H NMR spectrum of **6** in $CDCl_3$	**Spectrum S32.** ^{13}C NMR and DEPT spectra of **6** in $CDCl_3$
**Spectrum S33.** HSQC spectrum of **6** in $CDCl_3$	**Spectrum S34.** $^{1}H–^{1}H$ COSY spectrum of **6** in $CDCl_3$
**Spectrum S35.** HMBC spectrum of **6** in $CDCl_3$	**Spectrum S36.** NOESY spectrum of **6** in $CDCl_3$
**Spectrum S37.** ^{1}H NMR spectrum of **7** in $CDCl_3$	**Spectrum S38.** ^{13}C NMR and DEPT spectra of **7** in $CDCl_3$
**Spectrum S39.** HSQC spectrum of **7** in $CDCl_3$	**Spectrum S40.** $^{1}H–^{1}H$ COSY spectrum of **7** in $CDCl_3$
**Spectrum S41.** HMBC spectrum of **7** in $CDCl_3$	**Spectrum S42.** NOESY spectrum of **7** in $CDCl_3$
**Spectrum S43.** ^{1}H NMR spectrum of **8** in $CDCl_3$	**Spectrum S44.** ^{13}C NMR and DEPT spectra of **8** in $CDCl_3$
**Spectrum S45.** ^{1}H NMR spectrum of **9** in $CDCl_3$	**Spectrum S46.** ^{13}C NMR and DEPT spectra of **9** in $CDCl_3$
**Spectrum S47.** ^{1}H NMR spectrum of **10** in $CDCl_3$	**Spectrum S48.** ^{13}C NMR and DEPT spectra of **10** in $CDCl_3$

S4. HRESIMS spectra of **1**–**7**·····p47–50

**Spectrum S49.** HRESIMS spectrum of **1**	**Spectrum S50.** HRESIMS spectrum of **2**
**Spectrum S51.** HRESIMS spectrum of **3**	**Spectrum S52.** HRESIMS spectrum of **4**
**Spectrum S53.** HRESIMS spectrum of **5**	**Spectrum S54.** HRESIMS spectrum of **6**
**Spectrum S55.** HRESIMS spectrum of **7**	

S5. IR spectra of **1**–**7**·····p51–57

**Spectrum S56.** The IR spectrum of **1**	**Spectrum S57.** The IR spectrum of **2**
**Spectrum S58.** The IR spectrum of **3**	**Spectrum S59.** The IR spectrum of **4**
**Spectrum S60.** The IR spectrum of **5**	**Spectrum S61.** The IR spectrum of **6**
**Spectrum S62.** The IR spectrum of **7**	

S1. Figures

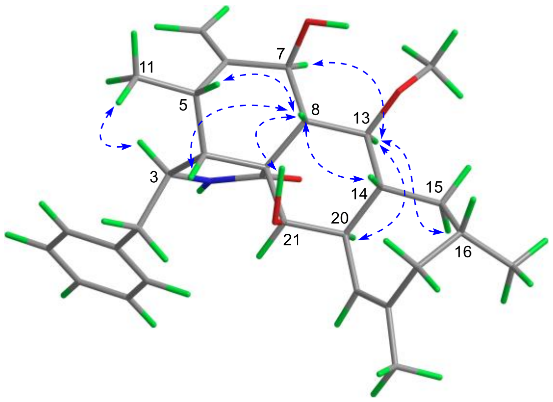

5

Figure S1. Key NOE correlations of compound **5**

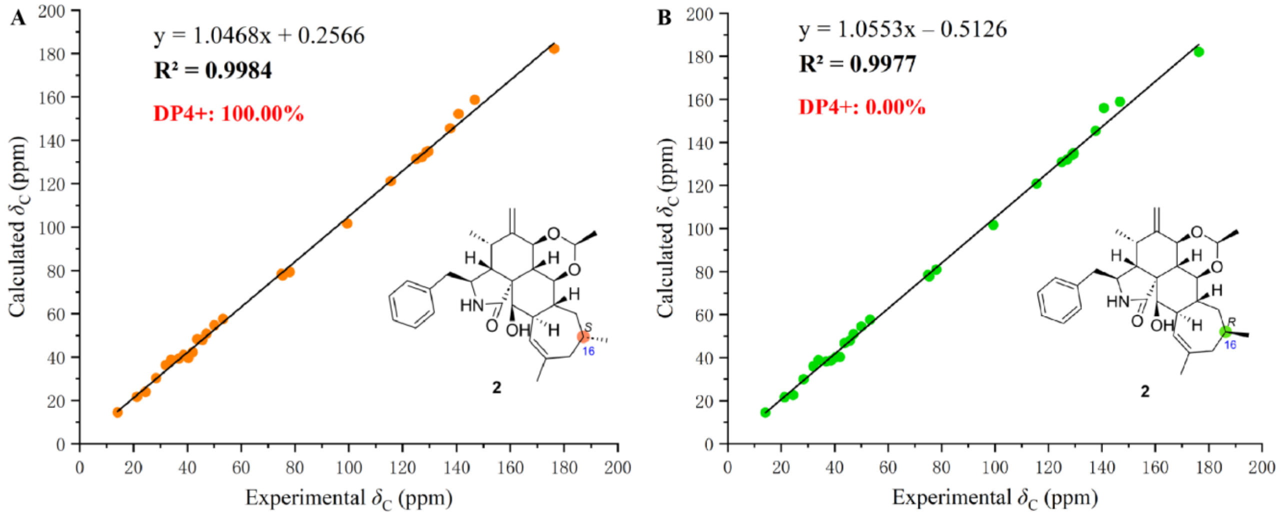

Figure S2. DP4+ analyses between experimental ^{13}C NMR chemical shifts of **2** and calculated ^{13}C NMR chemical shifts of 16*S*-**2a** (A) and 16*R*-**2b** (B).

4

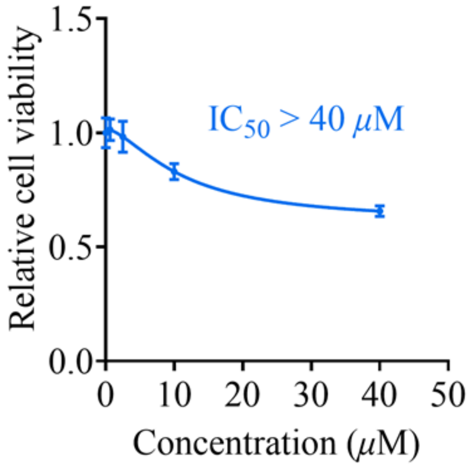

Figure S3. The effect of compound **4** on HEK-293T cell viability.

S2. Experimental section

S2.1. ECD calculations of compounds **1–3**, and **7**

The absolute configurations of **1–3**, and **7** were determined by quantum chemical calculations of their theoretical ECD spectra. One of the two enantiomers for each compound (3S*,4R*,5S*,7S*,8S*,9R*,13S*,14R*,16S*)-**1**, (3S*,4R*,5S*,7S*,8S*,9R*,13S*,14R*,16S*,20R*,21R*,1''S*)-**2**, (3S*,4R*,5S*,7S*,8R*,9R*,13S*,14R*,16S*,20R*,21R*)-**3**, and (3S*,4R*,5S*,8S*,9R*,13S*,14R*,16S*,20R*,21R*)-**7**, were arbitrary chosen for theoretical studies. Conformational analyses were first carried out via Monte Carlo searching using molecular mechanism with MMFF force field in the Spartan'14 software (Wavefunction Inc., Irvine, CA, USA). The results showed two lowest energy conformers for **1**, three for **2**, seven for **3**, and ten for **7** within an energy window of 10.0 Kcal/mol. These conformers were reoptimized using DFT at the B3LYP/6-31G(d) level[1] in gas phase using the Gaussian09[2]. Two conformers of **1** (Figure S4), three conformers of **2** (Figure S5), four conformers of **3** (Figure S6), and six conformers of **7** (Figure S7), were obtained, whose relative Gibbs free energies in the range of 0−1.5 Kcal/mol were refined and considered for next step (Table S1). The energies, oscillator strengths, and rotational strengths of the first 30 electronic excitations were calculated using the TD-DFT methodology at the M062X[3]/TZVP[4] level in PCM (acetonitrile) (Tables S2-7). The results were visualized and exported using the SpecDis program[5](sigma = 0.30 for all). The calculated ECD spectra was generated as a sum of Gaussian curve using rotatory strengths computed in the dipole-velocity gauge from ECD data of the individual conformers. Boltzmann distributions of the conformers in equilibrium population were estimated from the relative Gibbs free energies (ΔG) at 298.15K.

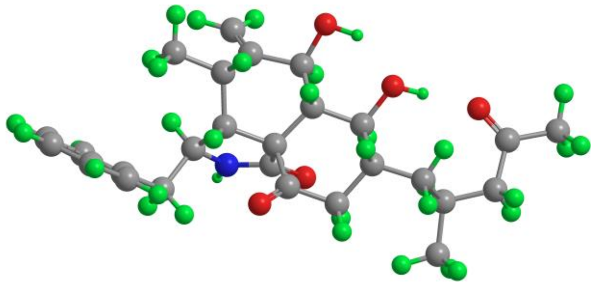

1C1

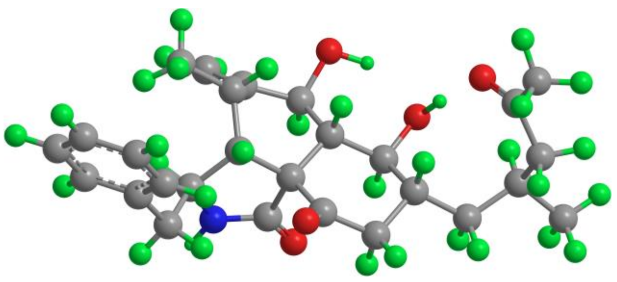

1C2

Figure S4. B3LYP/6-31G(d) optimized lowest energy conformers for **1**

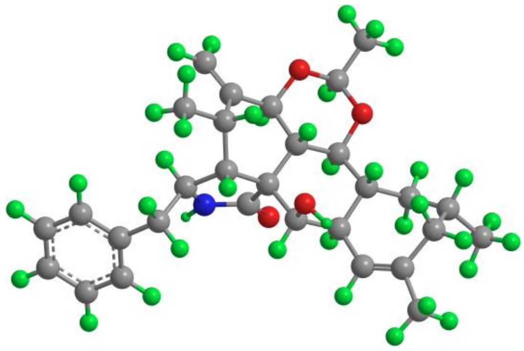

2C1

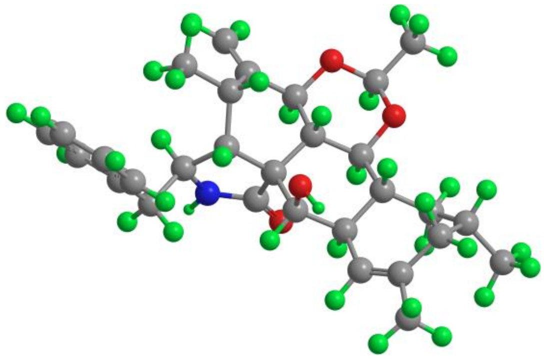

2C2

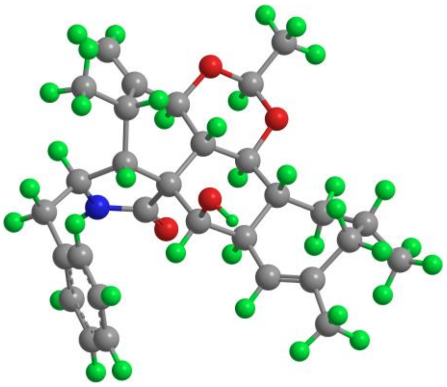

2C3

Figure S5. B3LYP/6-31G(d) optimized lowest energy conformers for **2**

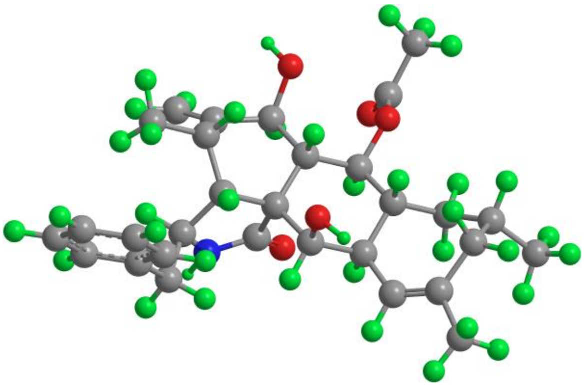

3C1

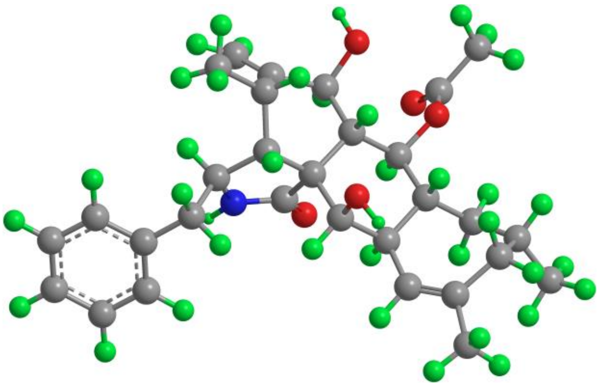

3C2

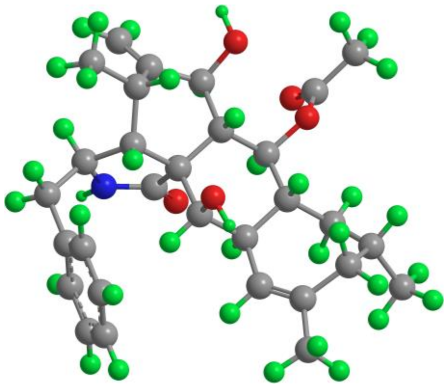

3C3

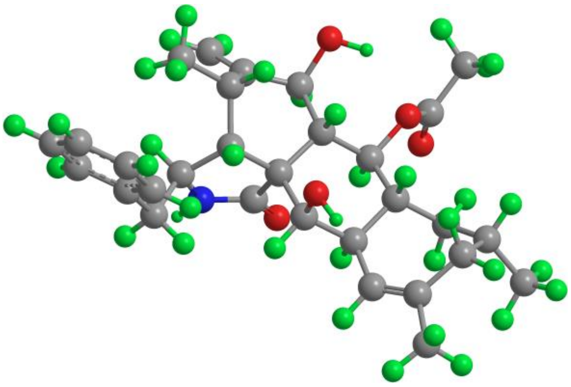

3C4

Figure S6. B3LYP/6-31G(d) optimized lowest energy conformers for **3**

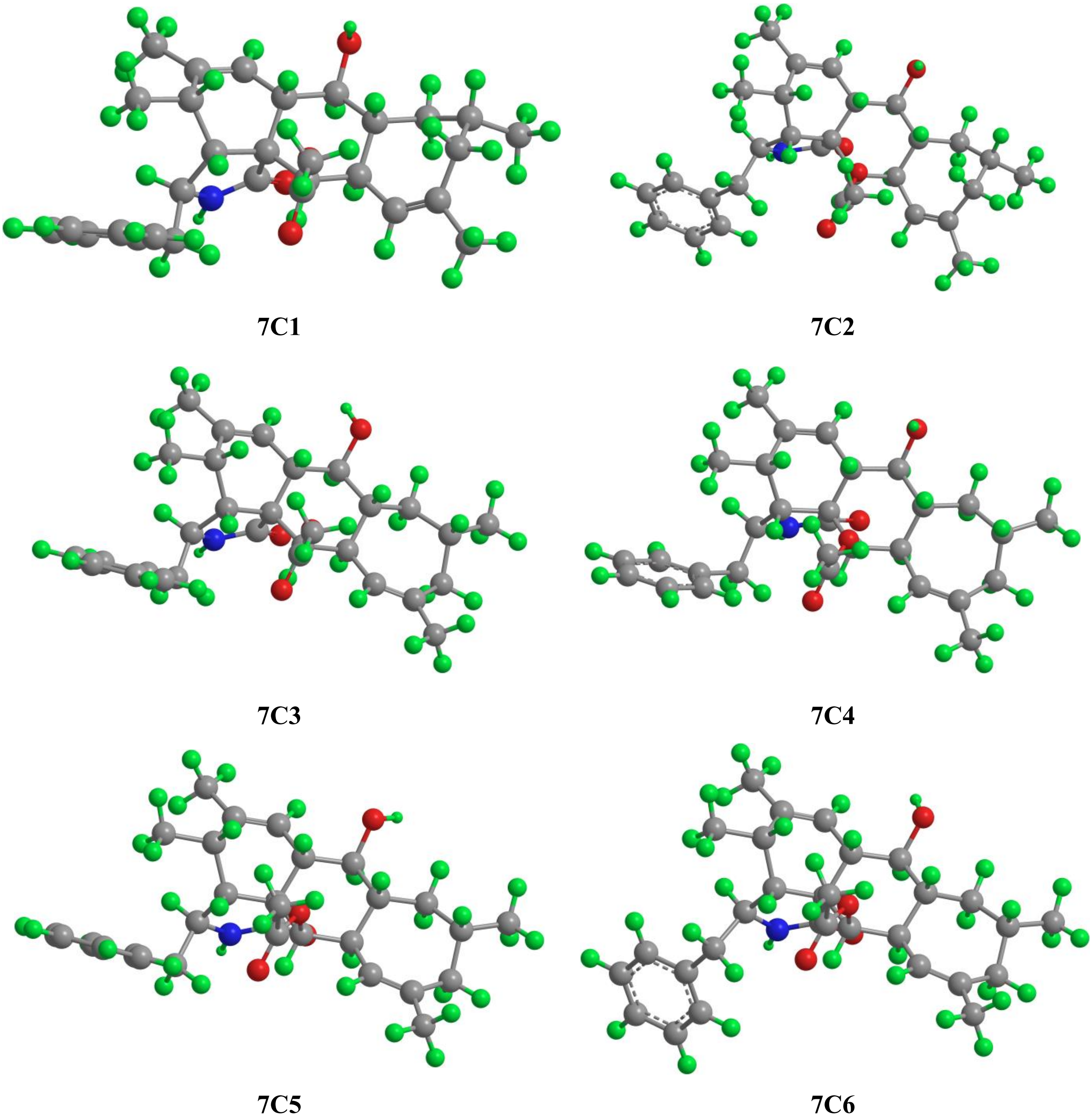

Figure S7. B3LYP/6-31G(d) optimized lowest energy conformers for **7**

Table S1. Energy (298.15 K) analysis for **1–3**, and **7**

Conf.	G (Hartree)	ΔG (Kcal/mol)	Boltzmann Distribution
**1C1**	−1480.8457971	0.7023	0.2341
**1C2**	−1480.8469163	0.0000	0.7659
**2C1**	−1522.3250736	0.0000	0.7500
**2C2**	−1522.3234632	1.0105	0.1362
**2C3**	−1522.3232926	1.1176	0.1137

Table S1 (continued). Energy (298.15 K) analysis for **1–3**, and **7**

Conf.	G (Hartree)	ΔG (Kcal/mol)	Boltzmann Distribution
**3C1**	−1597.5621236	0.6191	0.2129
**3C2**	−1597.5631102	0.0000	0.6052
**3C3**	−1597.5609027	1.3852	0.0584
**3C4**	−1597.5616094	0.9418	0.1235
**7C1**	−1522.3533859	1.3527	0.0516
**7C2**	−1522.3537554	1.1208	0.0763
**7C3**	−1522.3539469	1.0006	0.0934
**7C4**	−1522.3555415	0.0000	0.5059
**7C5**	−1522.3539776	0.9814	0.0965
**7C6**	−1522.3545459	0.6247	0.1762

ECD data

ECD spectrum of each conformation is simulated according to the overlapping Gaussian functions expressed as:

$$\Delta\varepsilon(E)=\frac{1}{2.296\times10^{-39}\sqrt{\pi}\sigma}\sum_{i}^{A}\Delta E_i R_i e^{[-(E-\Delta Ei)^2/\sigma^2]}$$

Where σ is half the bandwidth at 1/e peak height and expressed in energy units. The parameters ΔE_i and R_i are the excitation energies and rotational strengths for the transition *i*, respectively.

The above function is converted to Δε, λ (wavelength) correlations as:

$$\Delta\varepsilon(\lambda)=\frac{1}{2.296\times10^{-39}\sqrt{\pi}\sigma}\sum_{i}^{A}\Delta E_i R_i e^{[-(1240/\lambda-\Delta Ei)^2/\sigma^2]}$$

and then simulation was accomplished by using the Excel 2003 and the Origin 9.1 software.

To get the final spectra, all the simulated spectra of conformations of each compound were averaged according to their energy and the Boltzmann distribution theory expressed as:

$$\frac{N_i^*}{N}=\frac{g_i e^{-\varepsilon_i/k_B T}}{\sum g_i e^{-\varepsilon_i/k_B T}}$$

Table S2. Calculated ECD data of **1** (**1C1** and **1C2**) in acetonitrile

State	1C1		1C2	
	Excitation energies(eV)	Rotatory Strengths*	Excitation energies(eV)	Rotatory Strengths*
1	4.0922	0.5013	4.0871	−1.7867
2	4.4154	7.2645	4.4380	12.7062
3	5.3920	5.3292	5.3887	5.7626
4	5.4558	0.4245	5.4557	0.5437
5	6.1316	−33.7561	6.1271	−27.6297
6	6.1905	−0.7277	6.1888	−0.1993
7	6.6540	−34.5457	6.5959	−0.7013
8	6.7111	21.7056	6.6684	−19.9711
9	6.7541	−343.8635	6.7451	−358.7458
10	6.7915	20.8097	6.7858	−64.4122
11	6.8272	261.2681	6.8242	233.5742
12	6.8662	−77.0156	6.8387	211.0783
13	6.8891	52.6107	6.8871	−72.2646
14	7.0122	−18.5515	7.0019	−5.9444
15	7.0335	209.0640	7.0328	205.2529
16	7.1657	33.7649	7.1228	40.2705
17	7.2353	57.0835	7.1806	26.3391
18	7.3352	−59.4292	7.2603	0.1861
19	7.4378	51.3299	7.3312	−54.8466
20	7.4671	61.7387	7.4359	63.8822
21	7.4886	4.3397	7.4658	53.4348
22	7.5744	18.9602	7.4929	6.0575
23	7.5948	1.7003	7.5899	9.7055
24	7.6114	−4.7581	7.5947	1.1099
25	7.6426	−3.8041	7.6325	−3.6321
26	7.6587	−3.9232	7.6604	−1.2058
27	7.6630	1.5299	7.6752	−0.0082
28	7.6858	8.4832	7.6859	27.7748
29	7.6874	10.5885	7.6937	1.3529
30	7.7689	16.1982	7.6966	−12.2389

* R(velocity) 10**-40 erg-esu-cm

Table S3. Calculated ECD data of **2** (**2C1–2C3**) in acetonitrile

State	2C1		2C2		2C3	
	Excitation energies(eV)	Rotatory Strengths*	Excitation energies(eV)	Rotatory Strengths*	Excitation energies(eV)	Rotatory Strengths*
1	5.4626	0.6714	5.4526	0.4316	5.4630	–0.7900
2	5.5600	10.1434	5.5557	5.0935	5.5530	11.0823
3	6.1854	–30.4446	6.1682	–0.8653	6.1842	–17.3523
4	6.5033	–66.4503	6.5033	–47.4973	6.4814	–40.1309
5	6.6950	112.5096	6.6894	–547.7005	6.6911	–300.1471
6	6.7602	–315.3954	6.7661	680.5169	6.7275	337.0997
7	6.8163	72.6708	6.7974	–95.0046	6.7958	–90.3872
8	6.9873	–23.2195	6.9819	–2.8264	6.9408	–39.7653
9	7.0192	–25.2902	7.0192	–14.1105	6.9761	8.0129
10	7.0880	–34.9717	7.0736	–92.4883	7.0140	136.1605
11	7.1713	214.0510	7.1537	149.2690	7.0366	–9.7238
12	7.3274	1.3809	7.2897	1.8016	7.0783	–143.2755
13	7.3532	31.3137	7.3499	0.3216	7.1269	–10.9635
14	7.3795	–2.0837	7.3588	63.2596	7.1811	187.4934
15	7.4018	–7.6856	7.4313	–46.8097	7.2432	–24.3692
16	7.4538	23.5635	7.4422	10.6481	7.3158	0.0729
17	7.4772	–18.1519	7.4587	0.0253	7.4476	2.9344
18	7.5017	–12.1440	7.4672	–6.7640	7.4639	30.7684
19	7.5507	–9.1052	7.5577	–2.3244	7.5414	–2.5054
20	7.5796	3.6437	7.5885	–0.0754	7.5630	–29.8760
21	7.5979	–5.5381	7.6160	14.3801	7.5818	–12.3143
22	7.6187	13.2220	7.6327	10.9797	7.5978	6.0222
23	7.6440	–1.3436	7.6394	–1.3747	7.6267	4.7402
24	7.6497	8.8338	7.6570	–12.9675	7.6383	–2.1622
25	7.6832	5.2573	7.7172	–2.4692	7.6660	25.3378
26	7.7204	–0.8144	7.7492	1.4278	7.6905	1.0620
27	7.7711	–18.3273	7.7837	4.6419	7.7329	4.0316
28	7.8029	–4.6828	7.8073	–13.8082	7.7440	–19.7932
29	7.8434	–1.9951	7.8296	1.1107	7.7506	–9.8119
30	7.8720	–9.4178	7.8827	–16.8532	7.7612	3.3482

* R(velocity) 10**-40 erg-esu-cm

Table S4. Calculated ECD data of **3** (**3C1** and **3C2**) in acetonitrile

State	**3C1**		**3C2**	
	Excitation energies(eV)	Rotatory Strengths*	Excitation energies(eV)	Rotatory Strengths*
1	5.4572	0.3461	5.4638	0.7212
2	5.5294	12.9763	5.5210	12.1177
3	5.8049	3.0479	5.8038	3.7998
4	6.1760	−6.9858	6.1872	−33.2259
5	6.5146	−39.6828	6.5175	−81.2593
6	6.6712	−556.0993	6.6867	−124.0679
7	6.7643	−14.6244	6.7761	60.0889
8	6.8006	84.0116	6.8151	−101.7365
9	6.8360	528.3620	6.8538	−335.1936
10	6.9560	−71.0031	6.9494	−74.3927
11	7.0318	163.3417	7.0231	624.0411
12	7.2750	−1.4323	7.2802	−0.7901
13	7.3223	6.1533	7.3128	26.1109
14	7.3629	−0.8554	7.3438	−19.2246
15	7.3916	−30.6873	7.3902	1.6109
16	7.4208	−0.1586	7.4516	15.2022
17	7.4618	13.8231	7.4707	4.5973
18	7.4883	15.7091	7.4993	20.6115
19	7.5589	33.6670	7.5500	5.6771
20	7.5854	−26.4580	7.5944	5.4948
21	7.6129	0.7001	7.6053	−6.8980
22	7.6343	15.5574	7.6339	24.0290
23	7.6742	−7.9642	7.6463	−6.4602
24	7.6957	−4.5362	7.6753	−9.8376
25	7.7038	18.0089	7.6785	4.6977
26	7.7405	69.7823	7.6848	−0.5022
27	7.7546	10.7144	7.7266	34.9476
28	7.7664	−144.4829	7.7542	57.9665
29	7.8130	0.6352	7.7737	−93.8010
30	7.8259	−0.8941	7.7911	−63.6849

* R(velocity) 10**-40 erg-esu-cm

Table S5. Calculated ECD data of **3** (**3C3** and **3C4**) in acetonitrile

State	3C3		3C4	
	Excitation energies(eV)	Rotatory Strengths*	Excitation energies(eV)	Rotatory Strengths*
1	5.4563	−1.1197	5.4565	0.3351
2	5.5064	16.9574	5.5393	10.4258
3	5.8054	3.2391	5.7072	−3.2687
4	6.1725	−16.1340	6.1753	−5.1884
5	6.4718	−28.7512	6.5263	−38.4897
6	6.6666	−131.7898	6.6943	−552.8751
7	6.7657	225.7289	6.7974	25.7018
8	6.7850	−41.7580	6.8527	510.9545
9	6.8470	−22.2247	6.9696	−58.2758
10	6.8926	−144.1860	6.9998	−83.5469
11	6.9157	−3.5667	7.0593	311.2267
12	6.9676	−74.3400	7.2938	−0.0718
13	6.9936	215.0385	7.3284	16.4586
14	7.1361	19.9304	7.3858	−1.3169
15	7.2203	1.0227	7.4074	−32.2630
16	7.2618	−0.0340	7.4443	4.9531
17	7.3612	15.7875	7.4668	7.3783
18	7.4486	55.6600	7.5049	−3.9250
19	7.4805	11.8819	7.5085	−1.0340
20	7.5471	−37.0421	7.5790	4.2367
21	7.5784	−4.1016	7.5870	12.7629
22	7.6075	−6.1873	7.6129	2.4393
23	7.6197	30.2729	7.6530	7.5617
24	7.6292	−9.5055	7.6761	−9.0202
25	7.6623	−0.1319	7.7077	−23.3208
26	7.6815	5.6008	7.7209	−14.5487
27	7.6882	7.7969	7.7465	−2.1708
28	7.7162	−9.3118	7.7964	22.3037
29	7.7247	1.3462	7.8163	10.0906
30	7.7583	−71.7587	7.8191	−23.7793

* R(velocity) 10**-40 erg-esu-cm

Table S6. Calculated ECD data of **7** (**7C1–7C3**) in acetonitrile

State	**7C1**		**7C2**		**7C3**	
	Excitation energies(eV)	Rotatory Strengths*	Excitation energies(eV)	Rotatory Strengths*	Excitation energies(eV)	Rotatory Strengths*
1	5.4480	0.7176	5.4628	0.7901	5.4485	0.5641
2	5.5644	4.8645	5.5578	5.9239	5.5563	7.2986
3	5.7585	−1.9365	5.7677	−3.9777	5.7483	7.2986
4	6.1638	15.5595	6.1850	−37.9191	6.1636	21.1113
5	6.5787	−192.9276	6.5779	−234.5301	6.5749	−56.9154
6	6.5970	−67.3010	6.6123	133.7915	6.6199	208.2907
7	6.6450	16.8107	6.6493	1.4265	6.7110	−66.1037
8	6.7468	−22.1527	6.7504	−447.4340	6.7668	21.3697
9	6.7537	211.1046	6.8069	18.6820	6.7785	−63.2656
10	6.7820	−33.4037	6.8152	−44.3572	6.9170	0.0935
11	7.0075	−42.5935	6.9906	−29.7341	7.0510	−71.5704
12	7.0413	7.7083	7.0358	262.3256	7.1026	−37.9057
13	7.0741	−105.1027	7.1390	72.4376	7.1755	−13.2356
14	7.1343	59.8961	7.2192	14.0856	7.1885	47.4426
15	7.2339	13.4370	7.2469	9.1422	7.2957	19.4197
16	7.3010	4.5972	7.3259	1.6461	7.3289	1.0125
17	7.3660	19.4627	7.3682	−13.1653	7.3625	14.3173
18	7.3737	6.8209	7.3822	10.5959	7.3930	21.8513
19	7.3777	−25.5488	7.3986	10.6782	7.4072	6.0316
20	7.4039	3.9793	7.4224	4.1437	7.4256	−22.2741
21	7.4428	−6.7564	7.4813	15.6926	7.4722	−22.1840
22	7.4686	0.2634	7.4906	17.1808	7.4907	−4.2165
23	7.4927	40.7880	7.5132	−12.1797	7.5134	23.4707
24	7.5260	−8.8339	7.5354	−9.6621	7.5289	−4.7479
25	7.5662	27.0114	7.5650	−24.1378	7.5753	−19.5223
26	7.5828	−10.7260	7.5701	−0.1119	7.5889	−6.7753
27	7.5978	−7.0684	7.6122	0.4786	7.6018	7.8100
28	7.6133	−7.5006	7.6372	1.4579	7.6183	−2.7648
29	7.6375	−10.6435	7.6523	−13.9303	7.6365	−28.7303
30	7.6683	−14.8626	7.6817	1.1002	7.6829	0.6991

* R(velocity) 10**-40 erg-esu-cm

Table S7. Calculated ECD data of **7** (**7C4–7C6**) in acetonitrile

State	**7C4**		**7C5**		**7C6**	
	Excitation energies(eV)	Rotatory Strengths*	Excitation energies(eV)	Rotatory Strengths*	Excitation energies(eV)	Rotatory Strengths*
1	5.4483	0.5819	5.4482	0.5750	5.4628	0.6789
2	5.5530	6.1222	5.5568	6.0073	5.5453	10.5087
3	5.7471	−1.9590	5.7482	−1.8327	5.7500	−1.8383
4	6.1626	22.3623	6.1628	21.8256	6.1862	−31.4961
5	6.5884	−96.3849	6.5974	−171.4968	6.5986	−93.6042
6	6.6098	241.9309	6.6121	231.4675	6.6164	279.1897
7	6.6337	−61.9974	6.6712	45.9840	6.7254	−10.7107
8	6.7525	5.1638	6.7548	−16.5303	6.7567	−526.9973
9	6.7776	−50.6300	6.7775	−56.5342	6.8161	−31.0297
10	6.9082	−0.4926	6.9125	−0.8800	6.9550	22.7045
11	7.0330	−24.9998	7.0219	1.0276	7.0211	238.7159
12	7.0539	−95.5796	7.0543	−116.1298	7.1638	−2.4119
13	7.1378	52.9906	7.1190	57.0748	7.1686	21.8524
14	7.1542	−20.8771	7.1725	−15.5011	7.2494	6.9251
15	7.2933	24.8732	7.2922	24.0217	7.2968	−3.4434
16	7.3566	−13.3883	7.3510	−5.6012	7.3275	16.0982
17	7.3675	2.4719	7.3625	−33.0936	7.3435	35.6083
18	7.3729	−9.2531	7.3667	18.4317	7.4126	−34.3034
19	7.3945	−13.5938	7.3997	−18.5752	7.4227	−3.6614
20	7.4084	17.7567	7.4132	30.3739	7.4445	14.1672
21	7.4644	37.3460	7.4922	18.9470	7.4690	−6.3736
22	7.4957	3.0103	7.4961	−13.7945	7.5376	−24.3854
23	7.5385	−11.2161	7.5466	−27.2416	7.5531	−6.4703
24	7.5521	3.5770	7.5671	12.3088	7.5723	−10.6642
25	7.5792	−13.0818	7.5824	11.3668	7.6103	−26.8318
26	7.5924	−3.5718	7.5939	19.0164	7.6133	7.2485
27	7.6011	−2.9760	7.6069	−10.7481	7.6206	−0.5873
28	7.6191	−27.4598	7.6453	−8.3522	7.6355	5.7339
29	7.6394	31.7045	7.6643	−3.3878	7.6714	4.0272
30	7.6594	0.1424	7.6829	−0.5891	7.6880	−12.1515

* R(velocity)   10**-40 erg-esu-cm

S2.3. NMR calculation of 2

The candidate structures of 16*S*-**2a**, 16*R*-**2b** (Figure S8) were studied by quantum chemical Density functional theory (DFT) and time-dependent density functional theory (TDDFT) calculations of their theoretical 1D NMR chemical shifts. Conformational analyses were first carried out via Monte Carlo searching using molecular mechanism with MMFF force field in the Spartan'14 software (Wavefunction Inc., Irvine, CA, USA). The results showed eight lowest energy conformers for 16*S*-**2a** and six for 16*R*-**2b** within an energy window of 20.0 Kcal/mol. These conformers were reoptimized using the Gaussian09 (DFT at the B3LYP/6-31G(d) level in gas phase).

Six conformers of 16*S*-**2a** (Figure S9) and four conformers of 16*R*-**2b** (Figure S10) were obtained, whose relative Gibbs free energies in the range of 0−1.5 Kcal/mol were refined and considered for next step (Tables S8). Gauge-Independent Atomic Orbital (GIAO)[6] calculations of their ^{13}C NMR chemical shifts were accomplished by density functional theory (DFT) at the mPW1PW91/6-311+G(d,p) level[7] of theory with the solvent model PCM. The unscaled chemical shifts (δ_u) were computed using TMS as reference standard according to $\delta_u = \sigma_0 - \sigma_x$ (where σ_x is the Boltzmann averaged shielding tensor and σ_0 is the shielding tensor of TMS computed at the same level employed for σ_x). The Boltzmann averaging was done using the relative energies obtained from the single-point NMR calculations[8,9]. The experimental and calculated data [Table S9 for 16*S*-**2a** and Table S10 for 16*R*-**2b**] were analyzed by the improved probability DP4+ method [8,9] (Figure S11).

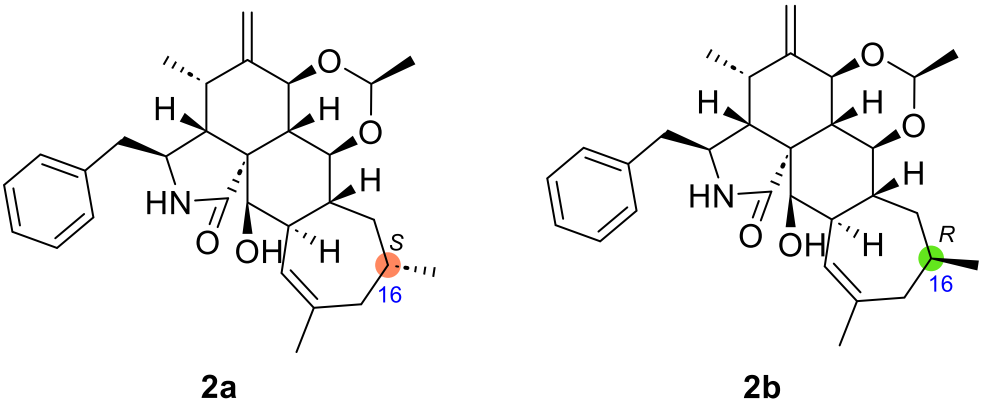

Figure S8. Two candidate structures of **2**.

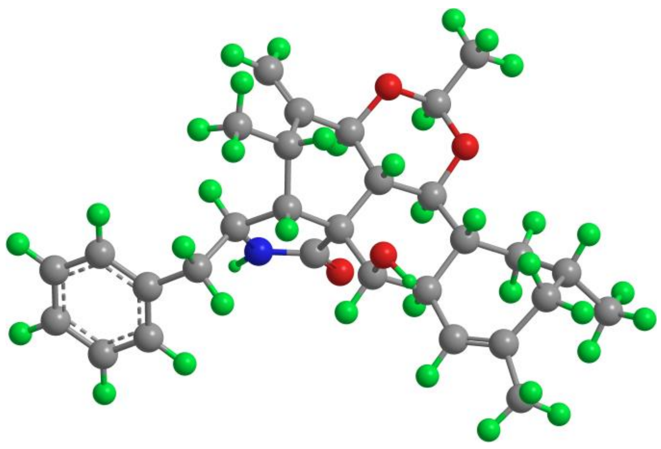

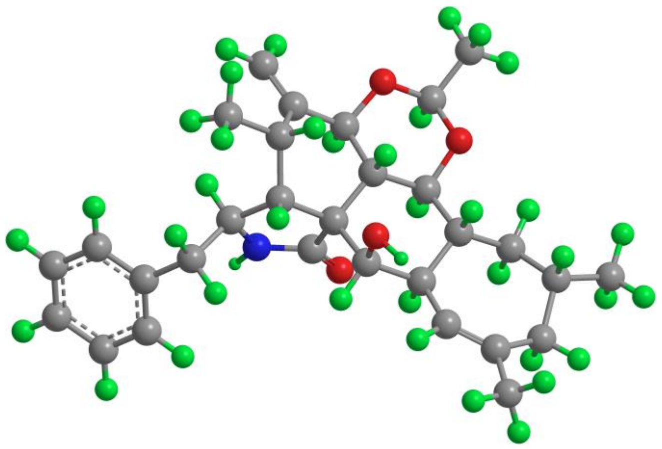

2aC1 **2aC2**

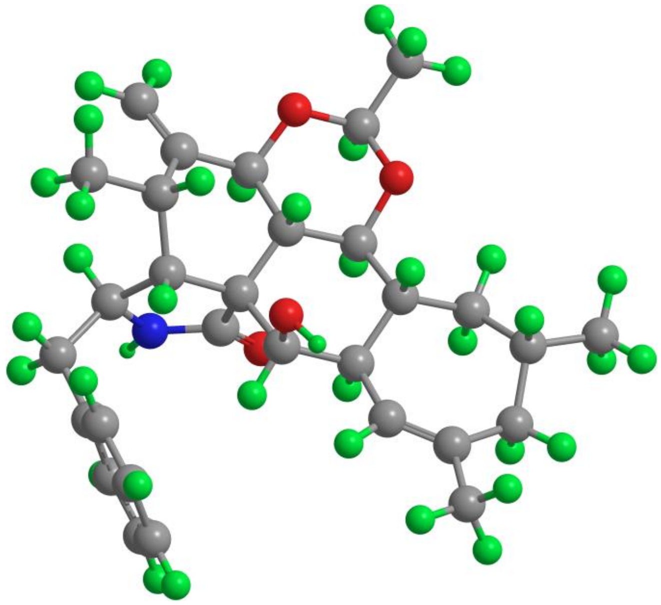

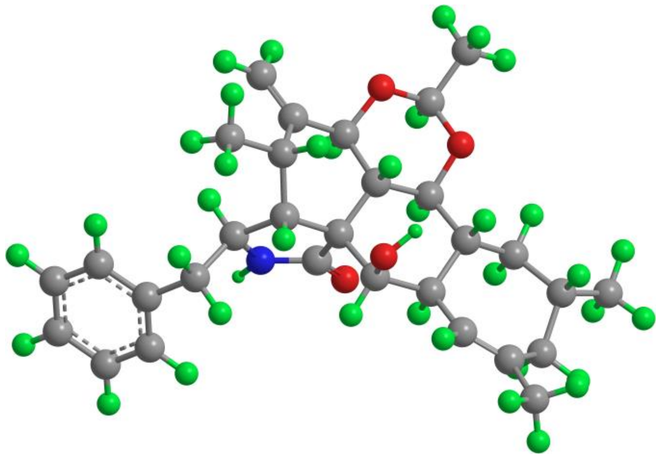

2aC3 **2aC4**

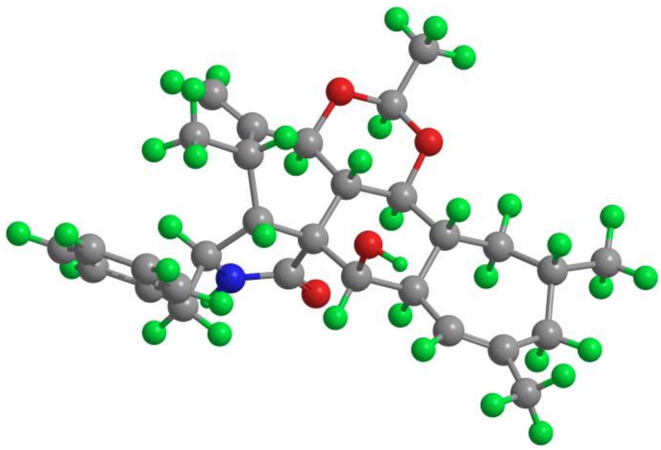

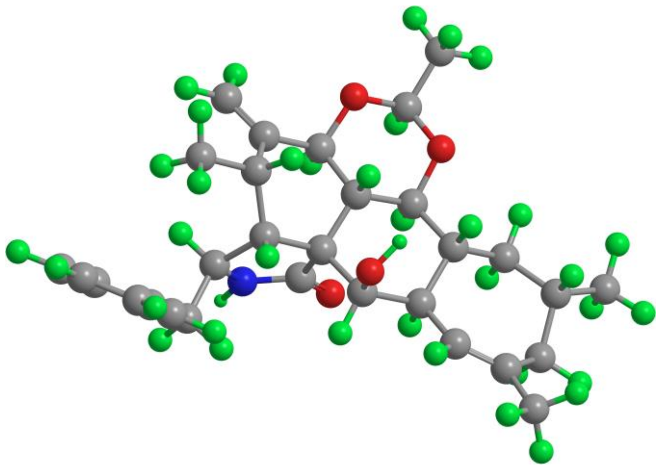

2aC5 **2aC6**

Figure S9. B3LYP/6-31G(d) optimized lowest energy conformers for 16*S*-**2a**.

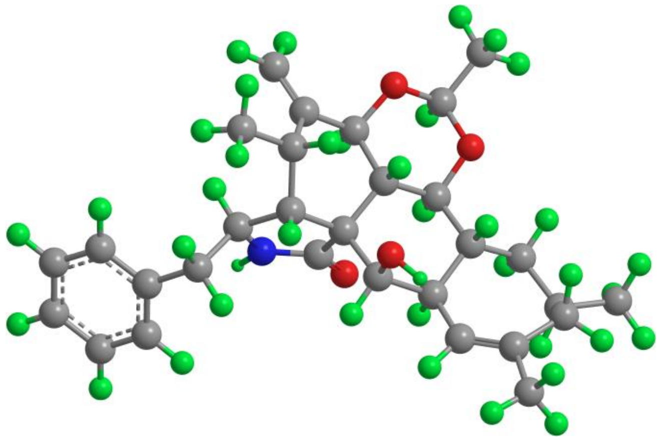

2bC1

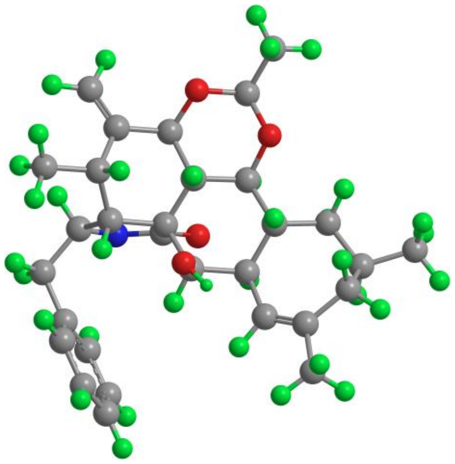

2bC2

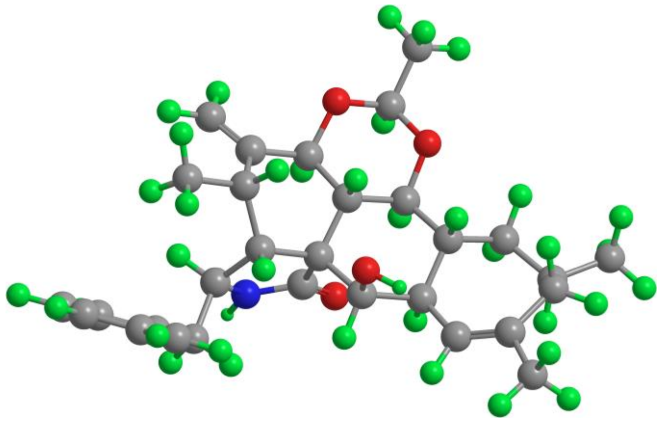

2bC3

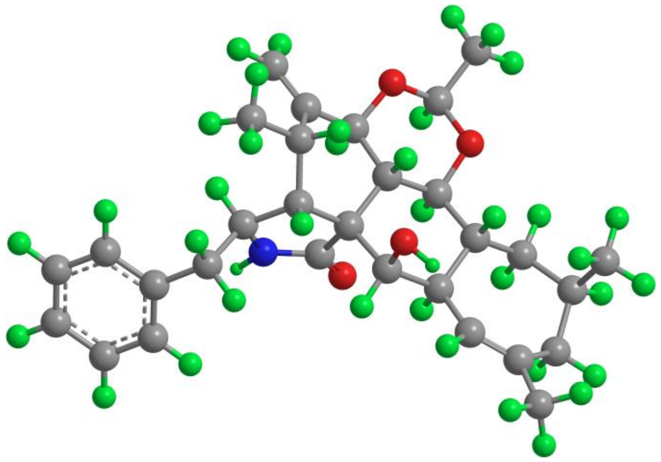

2bC4

Figure S10. B3LYP/6-31G(d) optimized lowest energy conformers for 16*R*-**2b**.

Table S8. Energy (298.15 K) analysis for **2**.

Conf.	G (Hartree)	ΔG (Kcal/mol)	Boltzmann Distribution
**2aC1**	−1522.3869473	0.0000	0.369548591
**2aC2**	−1522.3868923	0.0345	0.348635737
**2aC3**	−1522.3853022	1.0323	0.064704734
**2aC4**	−1522.3861282	0.5140	0.155199665
**2aC5**	−1522.3849053	1.2814	0.042497618
**2aC6**	−1522.3841656	1.7455	0.019413655
**2bC1**	−1522.3861768	0.0000	0.634165694
**2bC2**	−1522.3845472	1.0226	0.11287484
**2bC3**	−1522.3841162	1.2930	0.071505574
**2bC4**	−1522.3849954	0.7413	0.181453892

Table S9. Calculated ^{13}C-NMR chemical shifts for conformers of 16*S*-**2a**.

Position	**2aC1**	**2aC2**	**2aC3**	**2aC4**	**2aC5**	**2aC6**
1	182.2993	181.9223	182.7428	181.7275	182.4796	182.2618
3	57.885	57.8406	55.5723	57.8986	56.7574	56.8001
4	50.931	51.434	46.989	51.4948	48.7757	49.225
5	38.7339	38.6324	38.648	38.9532	37.9125	38.1849
6	158.6007	158.5866	159.4142	158.1013	158.8518	158.3335
7	77.4092	77.7003	77.7559	77.6166	78.3893	78.4439
8	40.4501	38.3712	38.619	38.6275	38.8722	38.8156
9	54.7263	54.3213	53.9751	55.6009	54.1891	55.4403
10	48.3908	48.364	43.3806	48.2687	48.0376	47.9224
11	14.3398	14.5691	14.3638	14.7362	13.1246	13.124
12	121.1301	121.0933	120.9263	121.2758	120.7349	121.0249
13	78.5855	79.5681	79.0627	79.7075	79.8551	80.1365
14	38.6015	42.6994	42.4895	41.6228	42.3538	41.0705
15	34.4334	41.7722	41.3058	44.975	41.5748	45.0598
16	37.4641	35.7618	35.6479	35.0841	35.7237	35.1942
17	36.8245	45.745	46.0474	43.9307	46.2429	43.85
18	150.5681	153.9974	153.1767	150.7953	154.2673	150.6168
19	129.6569	131.8426	132.1978	133.0803	131.8001	133.4327
20	52.0237	46.5766	46.5009	44.3518	46.967	44.4766
21	76.9973	79.6816	77.0895	78.7887	79.3421	78.3194
22	22.0287	25.0685	24.9488	25.0739	25.0059	25.0708
23	30.8251	30.623	30.4612	28.5691	30.6168	28.6471
1'	145.7668	145.6602	143.0331	145.6829	144.1364	144.002
2'	134.2639	134.5544	137.0739	134.676	135.7594	135.9292
3'	134.6791	134.6349	133.5779	134.6332	134.2278	134.3033
4'	132.1102	132.2413	132.043	132.3022	132.2064	132.2594
5'	134.1925	134.0822	133.6581	134.0646	134.5207	134.4497
6'	134.6228	134.8521	137.2318	134.9305	135.1109	135.1675
1''	101.3995	101.6102	101.8119	101.7216	102.0294	102.2434
2''	21.6035	21.643	21.631	21.6449	21.6792	21.6494

Table S10. Calculated ^{13}C-NMR chemical shifts for conformers of 16*R*-**2b**.

Position	**2bC1**	**2bC2**	**2bC3**	**2bC4**
1	181.9289	182.6358	182.5258	181.8354
3	57.8996	55.5553	56.8894	57.9038
4	51.6161	46.9707	48.9303	51.6222
5	38.9633	38.8638	37.8507	38.7833
6	158.9491	159.3015	158.7705	158.7421
7	77.6214	77.7616	78.3545	77.6963
8	38.2483	38.2527	38.6396	38.2757
9	54.437	54.1398	54.3042	54.3701
10	48.3998	43.3582	48.1369	48.4481
11	14.6305	14.3072	13.233	14.5936
12	120.8763	120.7452	120.4645	121.0426
13	81.0947	80.9143	81.6279	79.8685
14	39.3356	39.1338	38.655	35.4965
15	40.1407	39.8173	39.8459	40.3997
16	36.4662	36.7421	36.8666	33.2835
17	39.5314	39.6216	39.5651	43.5525
18	156.9182	156.1651	156.9803	152.2366
19	130.7588	130.8471	130.7912	130.9337
20	46.8794	46.5297	47.1071	46.4717
21	78.6723	75.8601	78.0148	79.4993
22	24.1436	23.9478	24.0645	16.1785
23	29.8473	29.5694	29.8539	30.8488
1'	145.7281	143.0534	144.0762	145.7277
2'	134.2691	137.036	135.8671	134.4388
3'	134.6823	133.6016	134.2932	134.6883
4'	132.1363	132.062	132.2123	132.1932
5'	134.209	133.819	134.4584	134.1498
6'	134.7184	137.3611	135.0632	134.7303
1''	101.6196	101.7934	101.9305	101.66
2''	21.5772	21.6374	21.68	21.597

Functional		Solvent?		Basis Set		Type of Data	
mPW1PW91		PCM		6-311+G(d, p)		Unscaled Shifts	
		DP4+	100. 00%	0. 00%	-	-	-
Nuclei	sp2?	Experimental	Isomer 1	Isomer 2	Isomer 3	Isomer 4	Isomer 5
C	x	176. 30	182. 11	182. 03			
C		53. 30	57. 65	57. 56			
C		47. 10	50. 81	50. 90			
C		34. 00	38. 68	38. 84			
C	x	146. 80	158. 58	158. 94			
C		75. 40	77. 63	77. 70			
C		36. 90	39. 23	38. 28			
C		50. 00	54. 66	54. 38			
C		45. 70	48. 01	47. 82			
C		14. 10	14. 41	14. 49			
C	x	115. 60	121. 11	120. 86			
C		78. 10	79. 22	80. 89			
C		38. 60	40. 96	38. 57			
C		40. 40	39. 58	40. 13			
C		32. 10	36. 27	35. 95			
C		42. 00	42. 17	40. 27			
C	x	140. 80	152. 13	155. 99			
C	x	125. 00	131. 28	130. 80			
C		43. 70	48. 22	46. 78			
C		75. 00	78. 34	78. 46			
C		24. 50	23. 94	22. 67			
C		28. 40	30. 33	30. 00			
C	x	137. 70	145. 44	145. 31			
C	x	129. 50	134. 71	134. 73			
C	x	128. 90	134. 56	134. 53			
C	x	127. 10	132. 19	132. 14			
C	x	128. 90	134. 12	134. 17			
C	x	129. 50	134. 95	135. 04			
C		99. 40	101. 59	101. 67			
C		21. 30	21. 63	21. 59			

Functional	Solvent?		Basis Set		Type of Data	
mPW1PW91	PCM		6-311+G(d, p)		Unscaled Shifts	
	Isomer 1	Isomer 2	Isomer 3	Isomer 4	Isomer 5	Isomer 6
sDP4+ (H data)	-	-	-	-	-	-
sDP4+ (C data)	98. 25%	1. 75%	-	-	-	-
sDP4+ (all data)	98. 25%	1. 75%	-	-	-	-
uDP4+ (H data)	-	-	-	-	-	-
uDP4+ (C data)	99. 96%	0. 04%	-	-	-	-
uDP4+ (all data)	99. 96%	0. 04%	-	-	-	-
DP4+ (H data)	-	-	-	-	-	-
DP4+ (C data)	100. 00%	0. 00%	-	-	-	-
DP4+ (all data)	100. 00%	0. 00%	-	-	-	-

Figure S11. DP4+ evaluation of theoretical and experimental data of **2.**

Reference

[1] (a) Becke, A. D. J. Chem. Phys. 1993, 98, 56485652. (b) Lee, T.; Yang, W. T.; Parr, R. G. Phys.Rev. B: Condens. Matter Mater. Phys. 1988, 37, 785789.

[2] Gaussian 09, Revision A.1, Frisch, M. J.; Trucks, G. W.; Schlegel, H. B.; Scuseria, G. E.; Robb, M.A.; Cheeseman, J. R.; Scalmani, G.; Barone, V.; Mennucci, B.; Petersson, G. A.; Nakatsuji, H.; Caricato, M.; Li, X.; Hratchian, H. P.; Izmaylov, A. F.; Bloino, J.; Zheng, G.; Sonnenberg, J. L.; Hada, M.; Ehara, M.; Toyota, K.; Fukuda, R.; Hasegawa, J.; Ishida, M.; Nakajima, T.; Honda, Y.; Kitao, O.; Nakai, H.; Vreven, T.; Montgomery, Jr., J. A.; Peralta, J. E.; Ogliaro, F.; Bearpark, M.; Heyd, J. J.; Brothers, E.; Kudin, K. N.; Staroverov, V. N.; Kobayashi, R.; Normand, J.; Raghavachari, K.; Rendell, A.; Burant, J. C.; Iyengar, S. S.; Tomasi, J.; Cossi, M.; Rega, N.; Millam, J. M.; Klene, M.; Knox, J. E.; Cross, J. B.;Bakken, V.; Adamo, C.; Jaramillo, J.; Gomperts, R.; Stratmann, R. E.; Yazyev, O.; Austin, A. J.; Cammi, R.; Pomelli, C.; Ochterski, J. W.; Martin, R. L.; Morokuma, K.; Zakrzewski, V. G.; Voth, G. A.; Salvador, P.; Dannenberg, J. J.; Dapprich, S.; Daniels, A. D.; Farkas, Ö.; Foresman, J. B.; Ortiz, J. V.; Cioslowski, J.; Fox, D. J. Gaussian, Inc., Wallingford CT, 2009.

[3] Zhao, Y.; Truhlar, D. G. Theor. Chem. Acc. 2008, 120, 215-241.

[4] Weigend, F.; Ahlrichs, R. Phys. Chem. Chem. Phys. 2005, 7, 32973305.

[5] Bruhn, T.; Schaumlöffel, A.; Hemberger, Y.; Bringmann, G. Chirality 2013, 25, 243249.

[6] (a) R. Ditchfield, J. Chem. Phys. 56, 56885691 (1972); (b) R. Ditchfield, Mol. Phys. 27, 789807 (1974); (c) K. Wolinski, J. F. Hinton, P. Pulay, J. Am. Chem. Soc. 112, 82518260 (1990).

[7] Lodewyk, M.W.; Soldi, C.; Jones, P. B.; Olmstead, M.M.; Rita, J.; Shaw, J. T.; Tantillo, D. J. J. Am. Chem. Soc. 2012, 134, 1855018552.

[8] Smith, S. G.; Goodman, J. M.; J. Am. Chem. Soc. 2010, 132, 1294612959.

[9] Grimblat, N.S.; Zanardi, M.M.; Sarotti, A. M. J. Org. Chem. 2015, 80, 1252612534.

S3. 1D and 2D NMR spectra of compounds **1–10**

Spectrum S1. ^{1}H NMR spectrum of **1** in $CDCl_3$

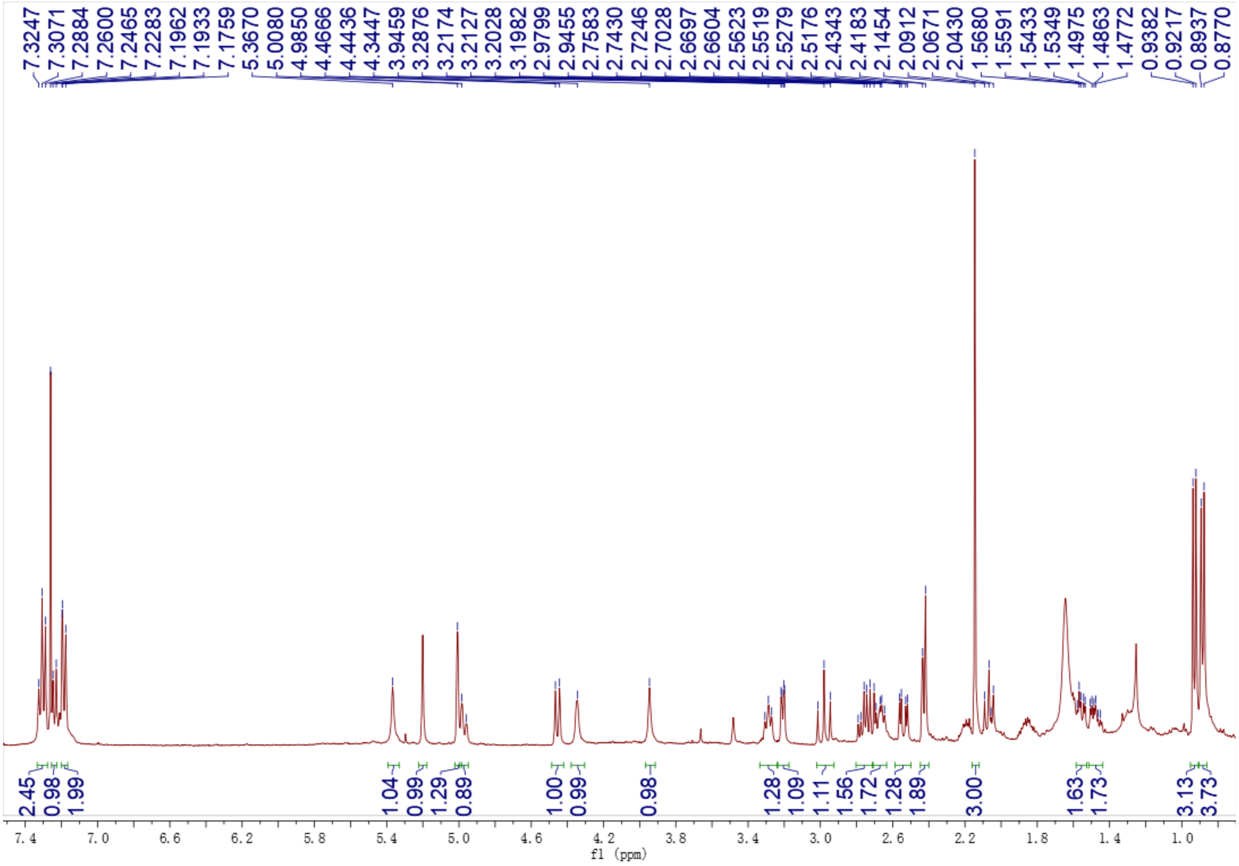

Spectrum S2. ^{13}C NMR and DEPT spectra of **1** in $CDCl_3$

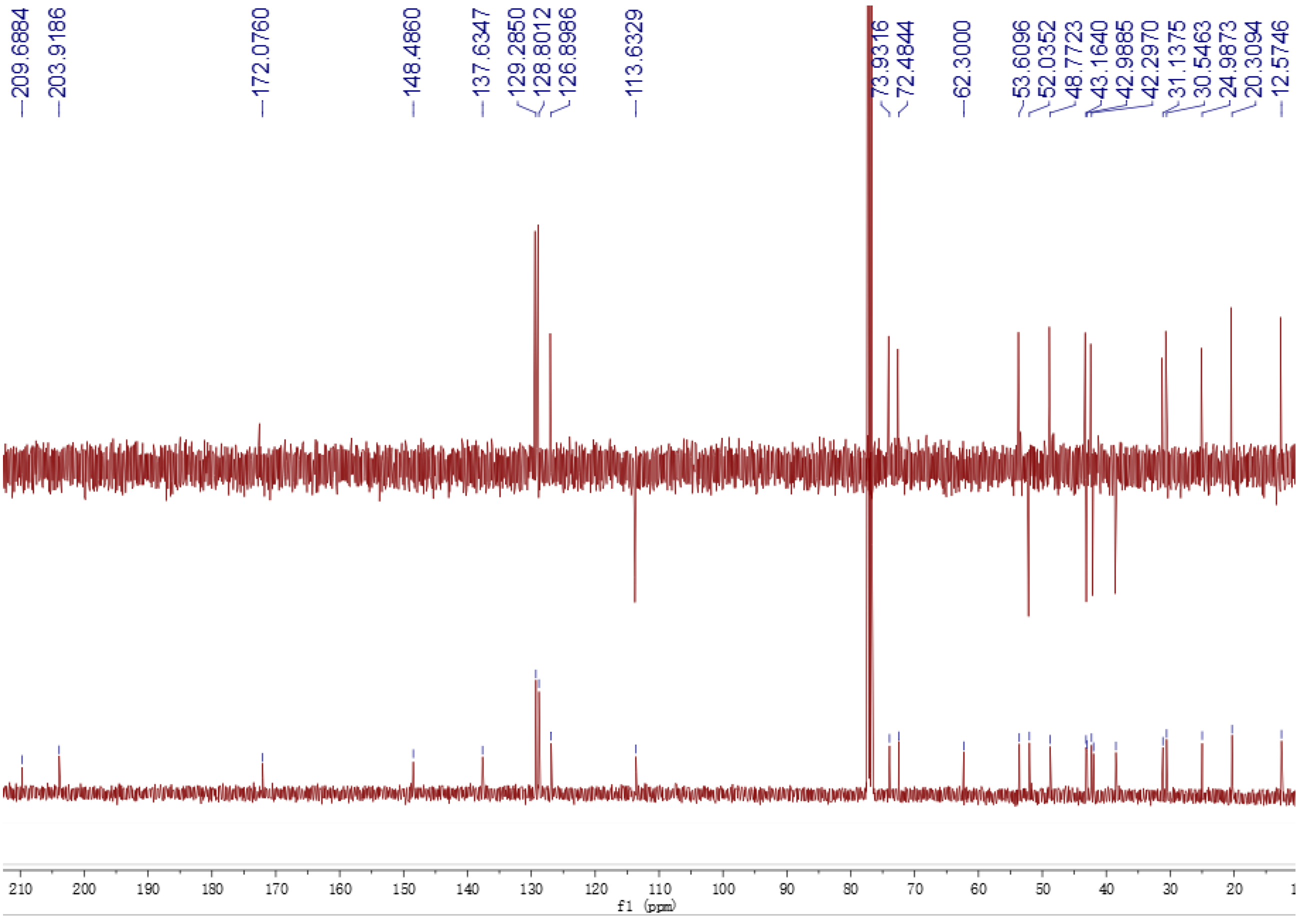

Spectrum S3. HSQC spectrum of **1** in $CDCl_3$

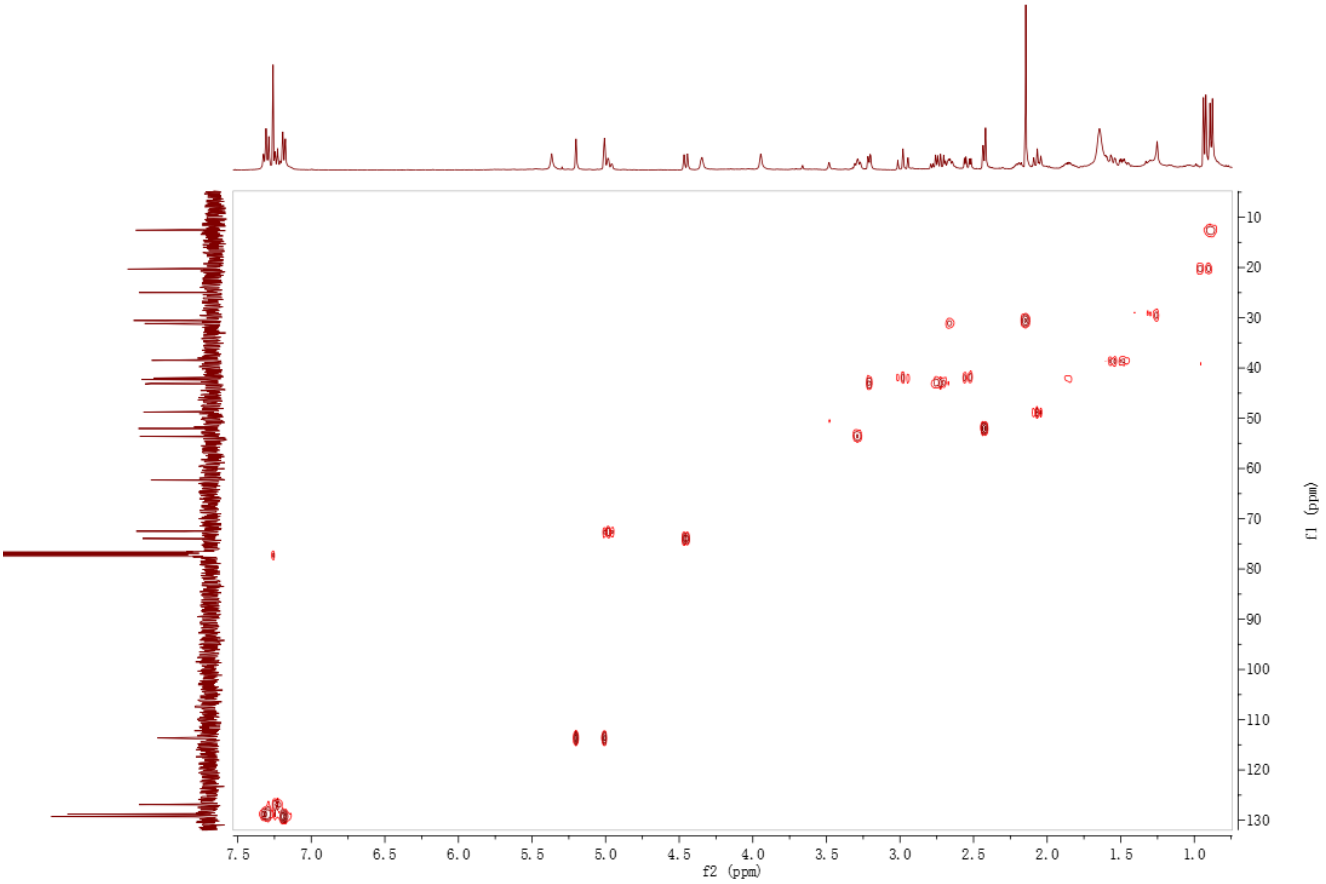

Spectrum S4. ^{1}H–^{1}H COSY spectrum of **1** in $CDCl_3$

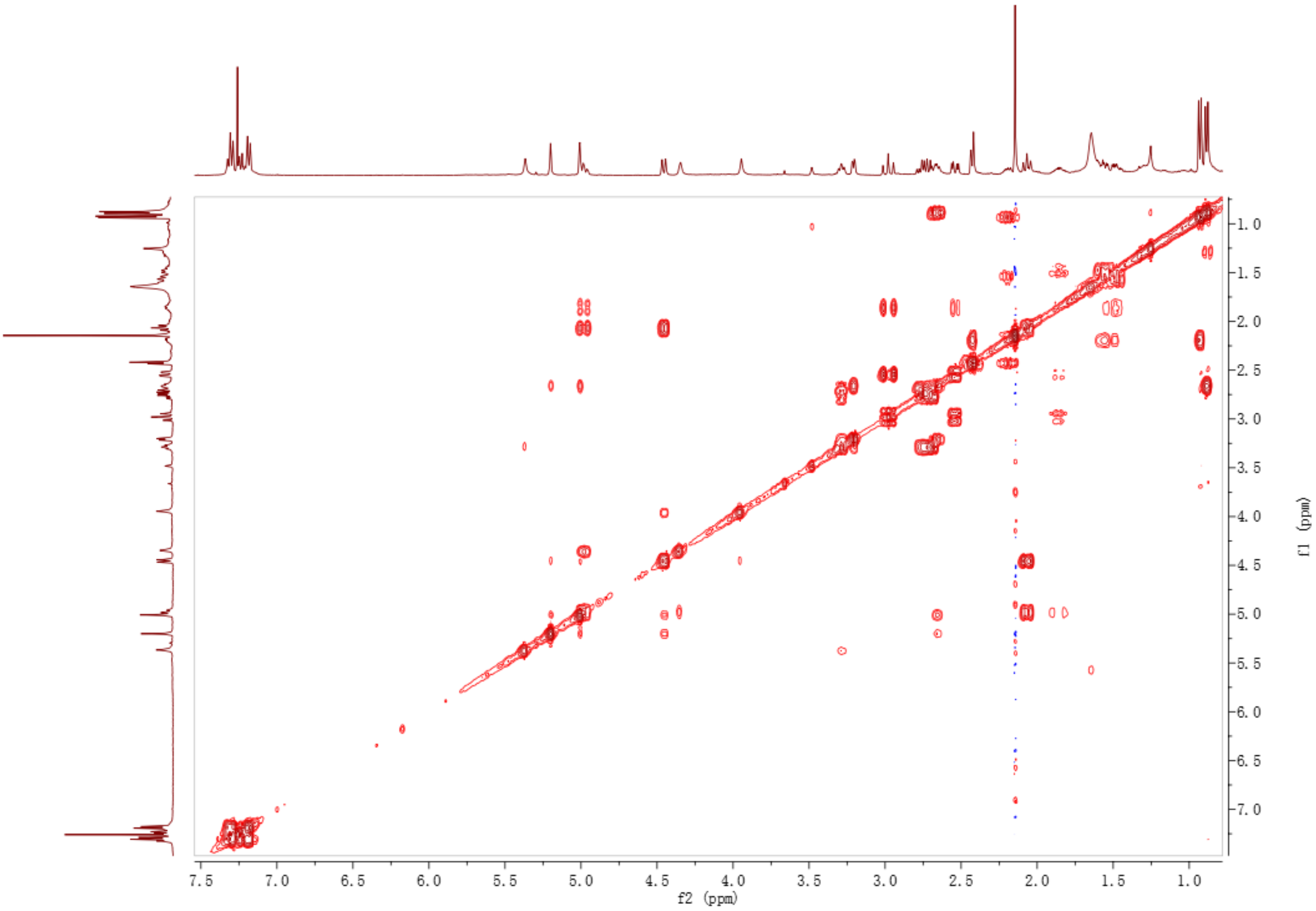

Spectrum S5. HMBC spectrum of **1** in $CDCl_3$

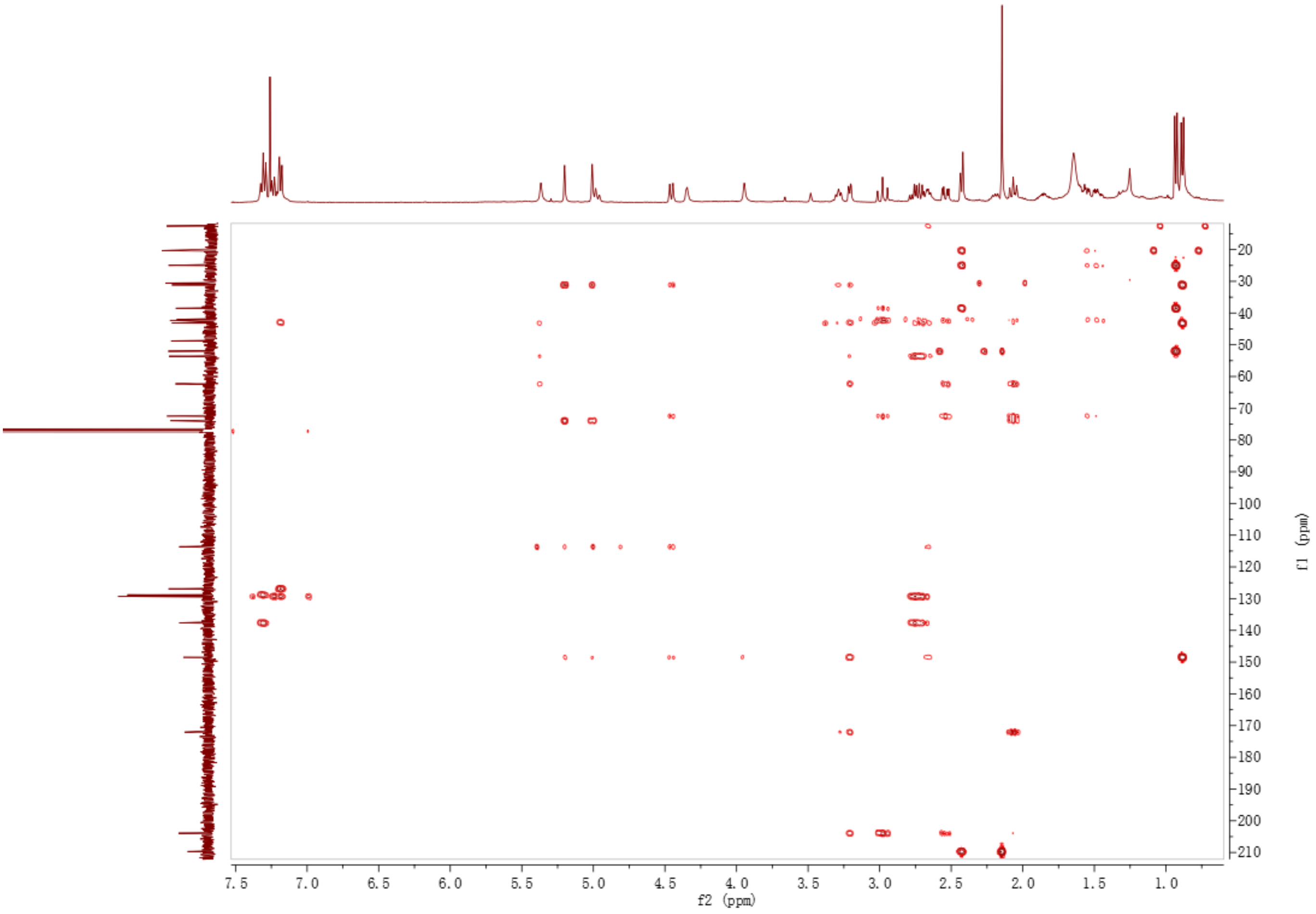

Spectrum S6. NOESY spectrum of **1** in $CDCl_3$

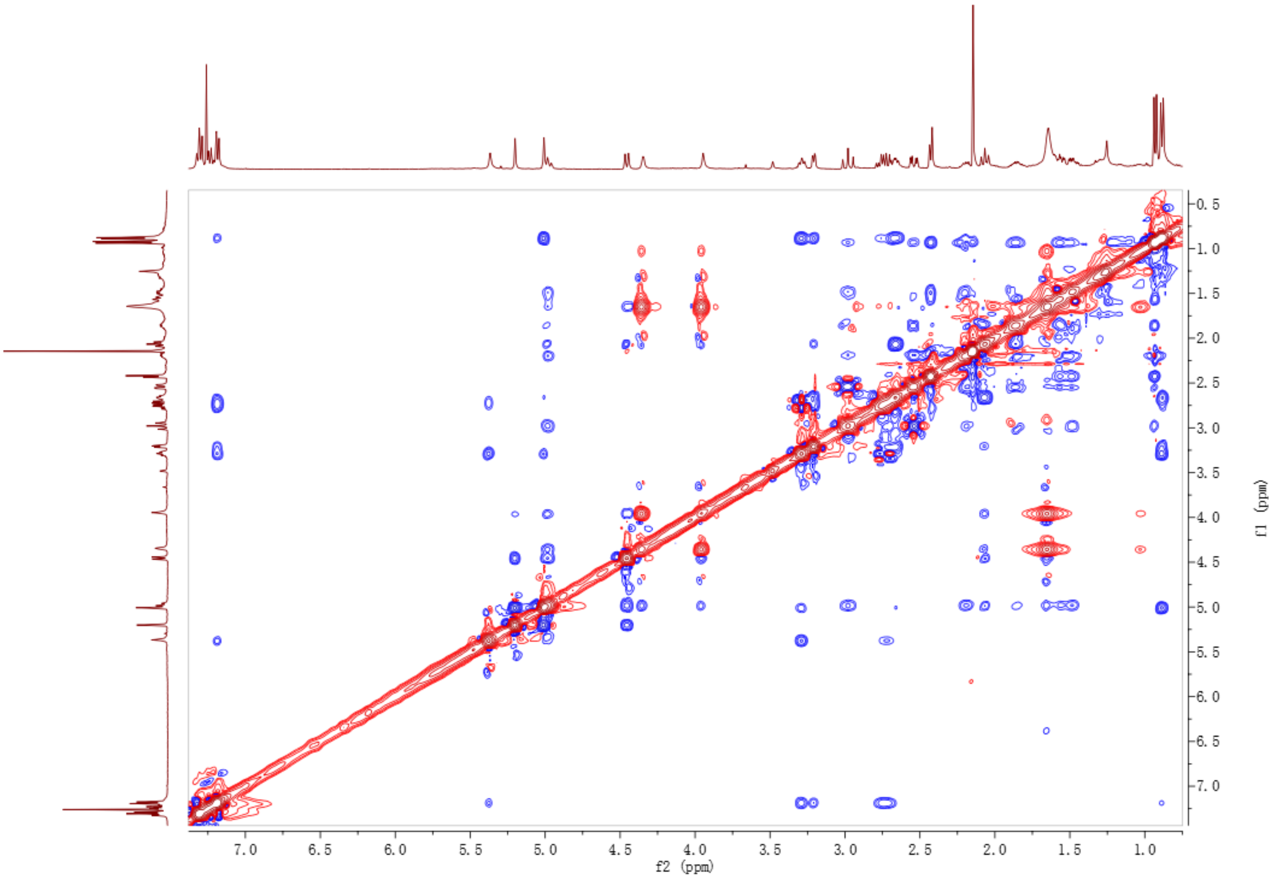

Spectrum S7. ^{1}H NMR spectrum of **2** in $CDCl_3$

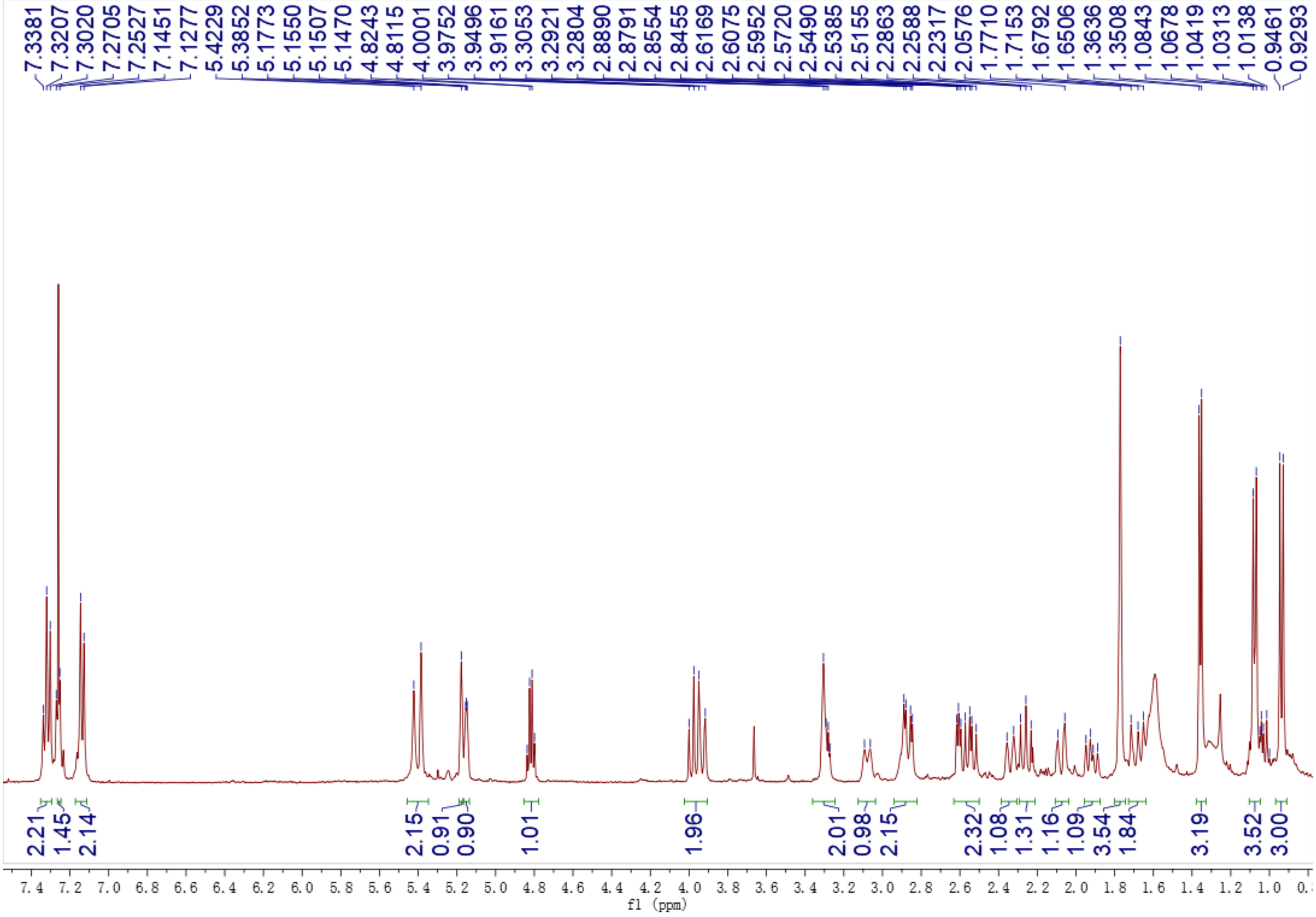

Spectrum S8. ^{13}C NMR and DEPT spectra of **2** in $CDCl_3$

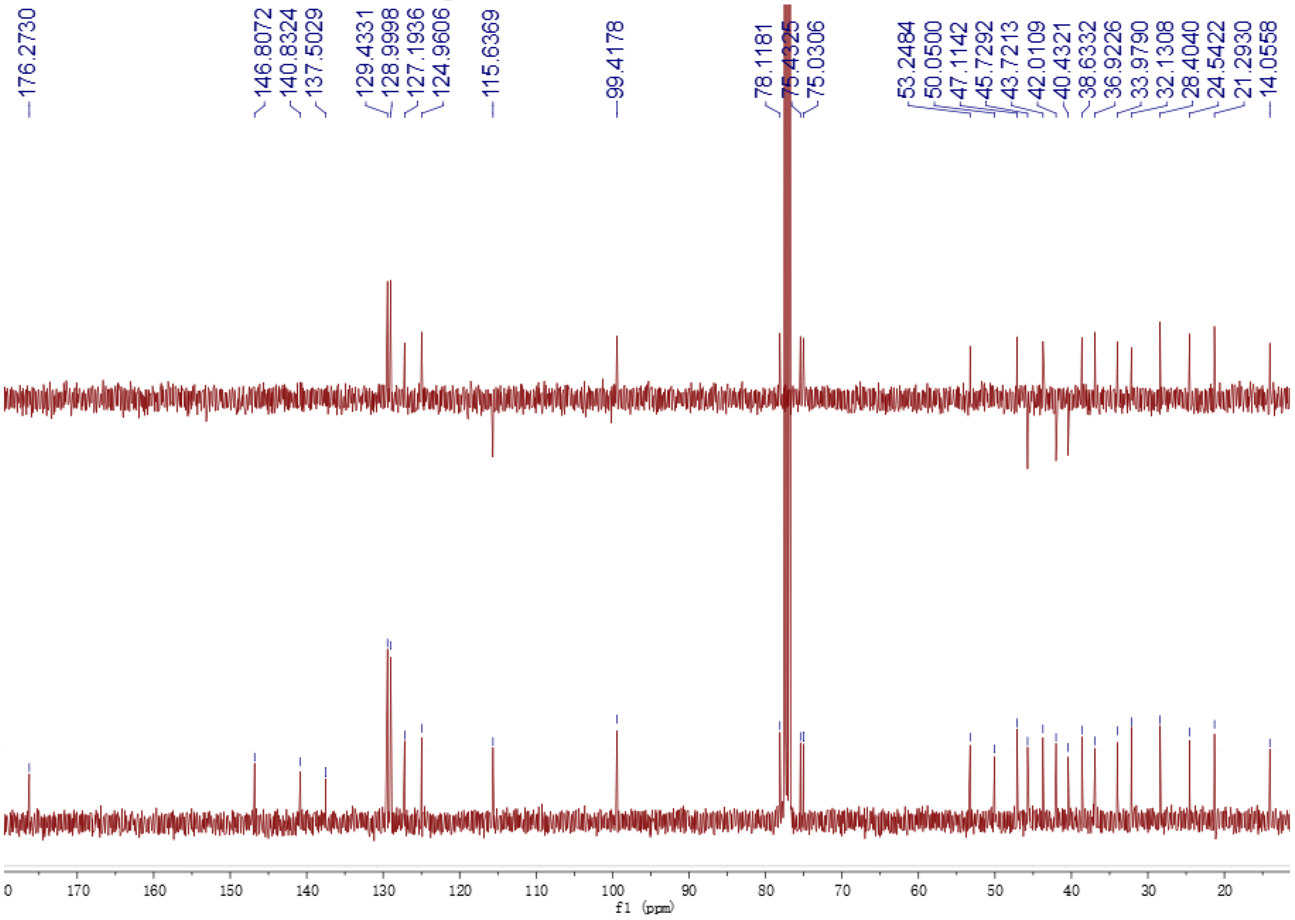

Spectrum S9. HSQC spectrum of **2** in $CDCl_3$

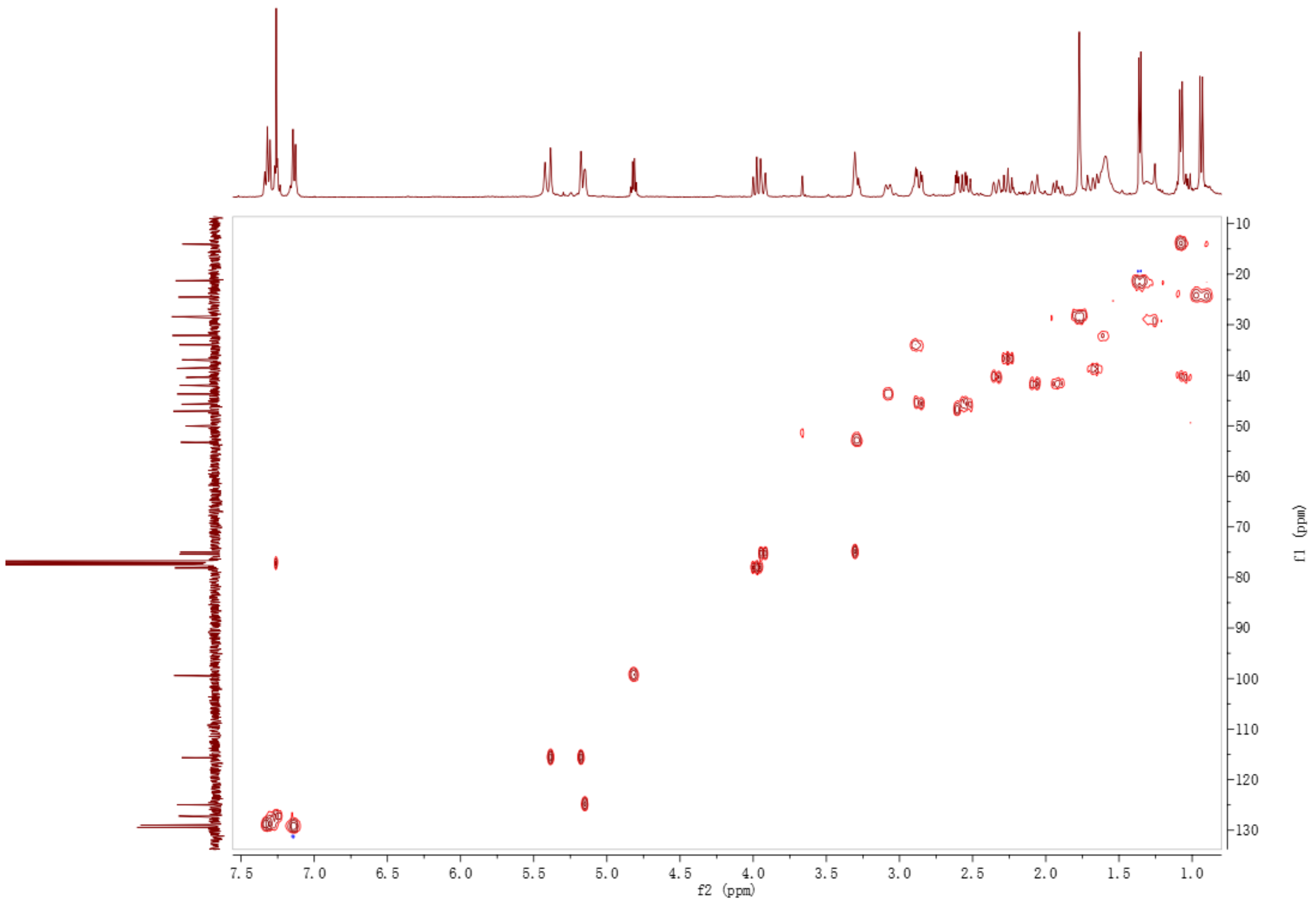

Spectrum S10. ^{1}H–^{1}H COSY spectrum of **2** in $CDCl_3$

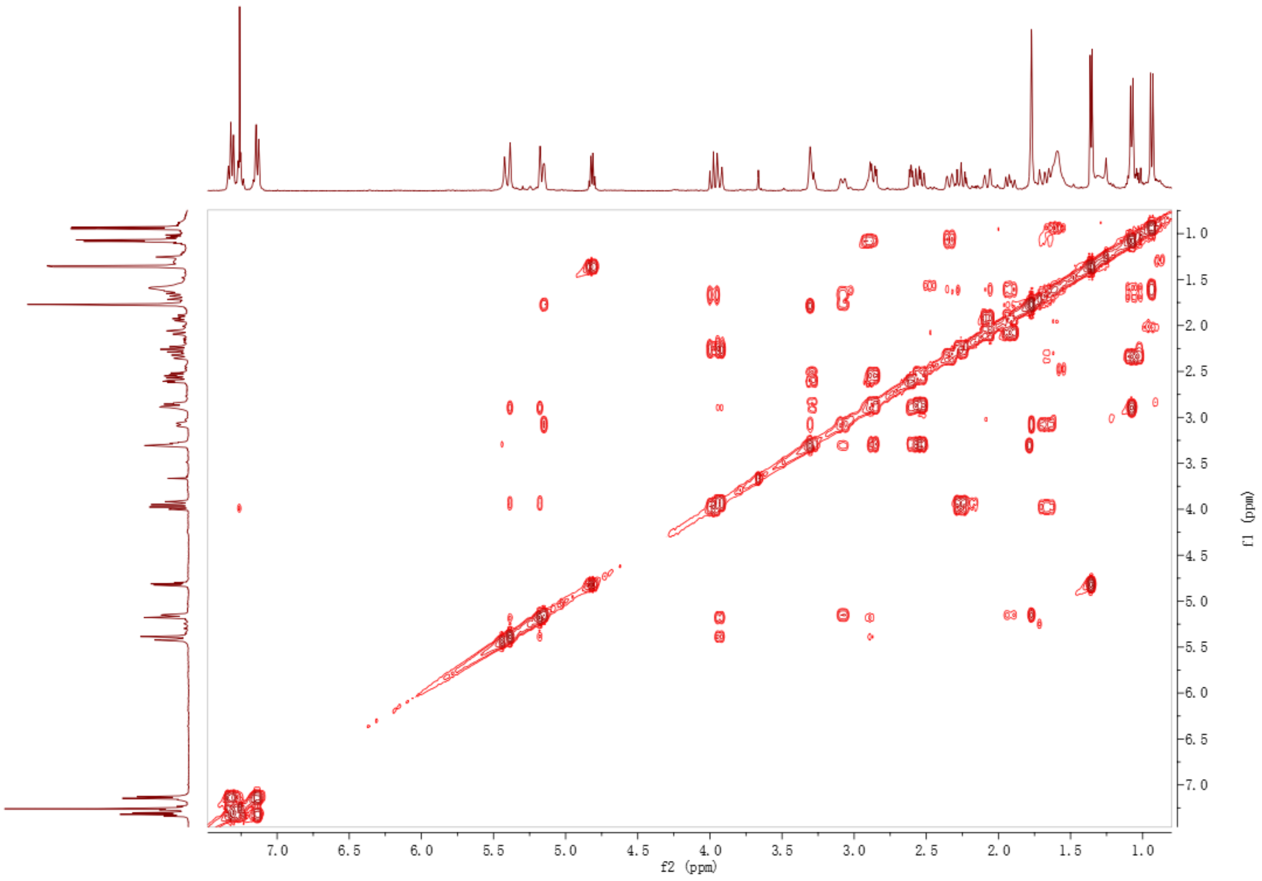

Spectrum S11. HMBC spectrum of **2** in $CDCl_3$

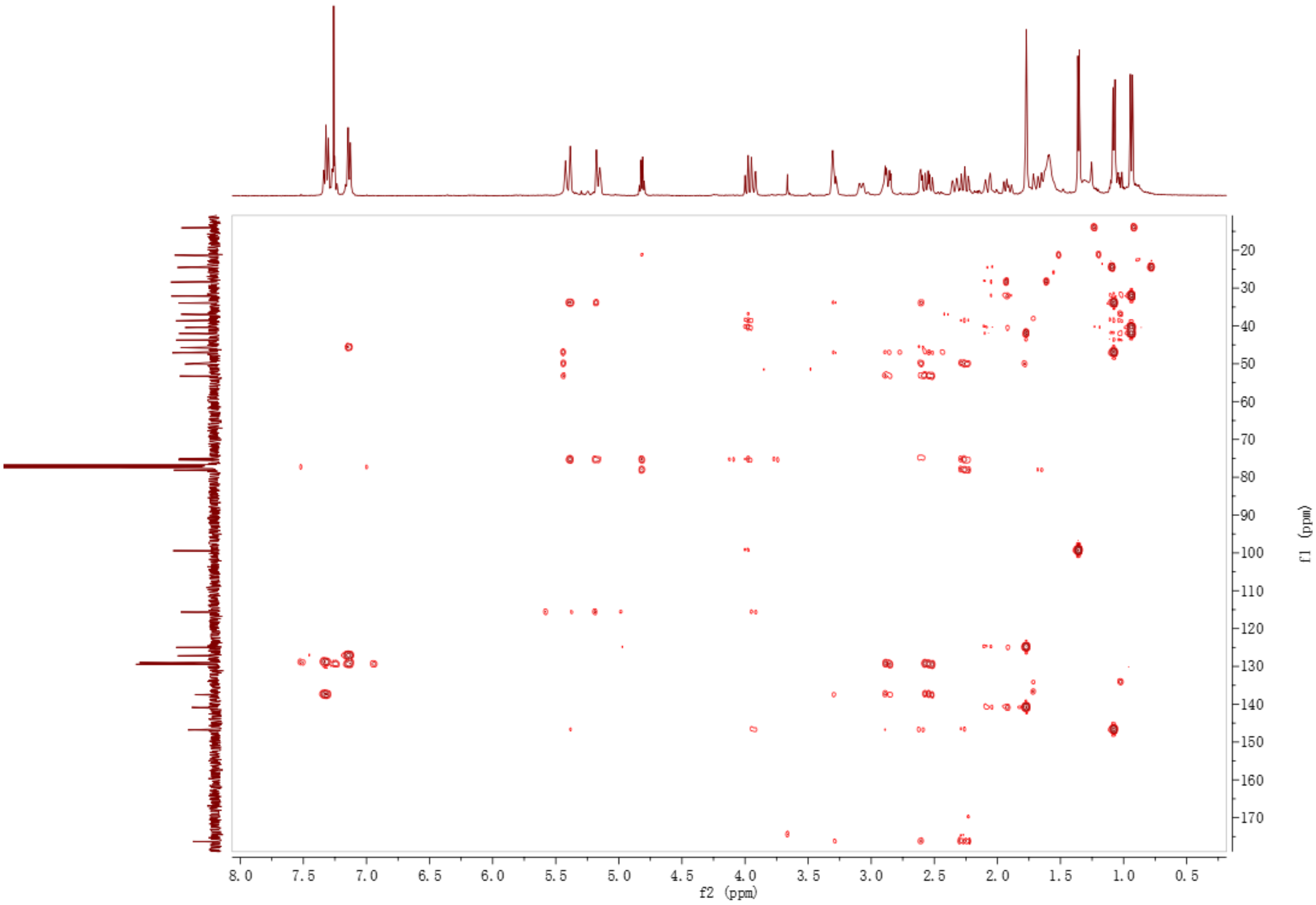

Spectrum S12. NOESY spectrum of **2** in $CDCl_3$

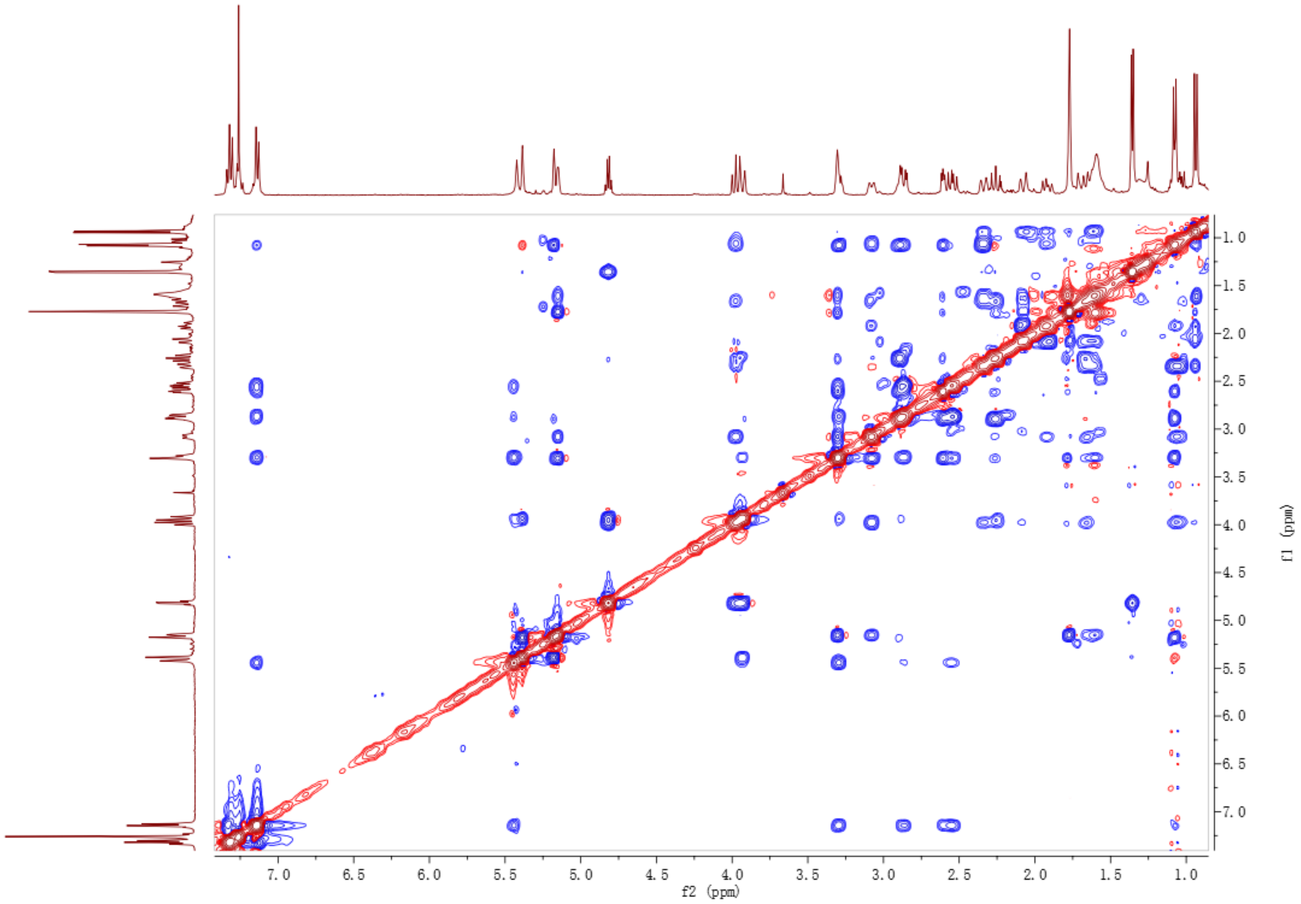

Spectrum S13. ^{1}H NMR spectrum of **3** in $CDCl_3$

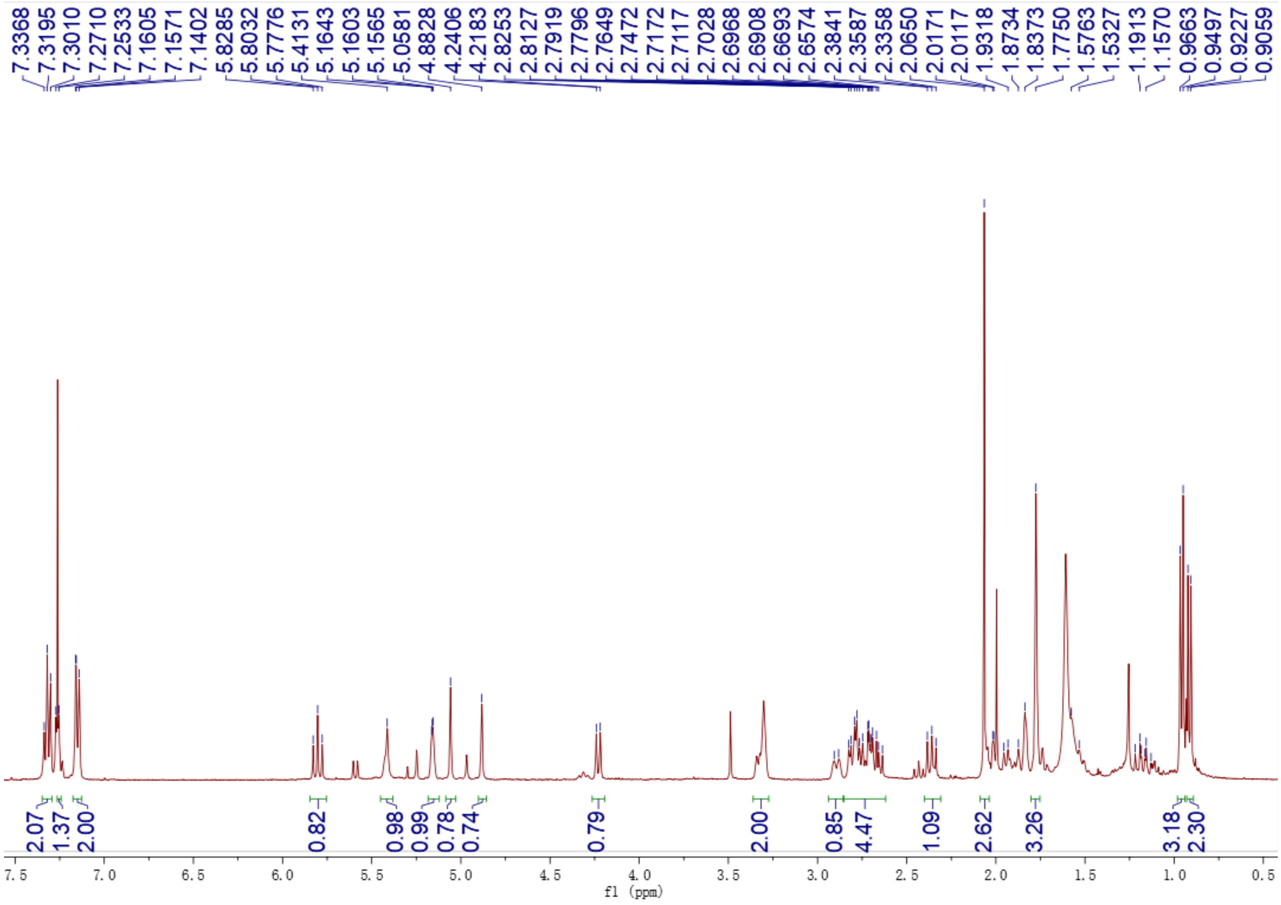

Spectrum S14. ^{13}C NMR and DEPT spectra of **3** in $CDCl_3$

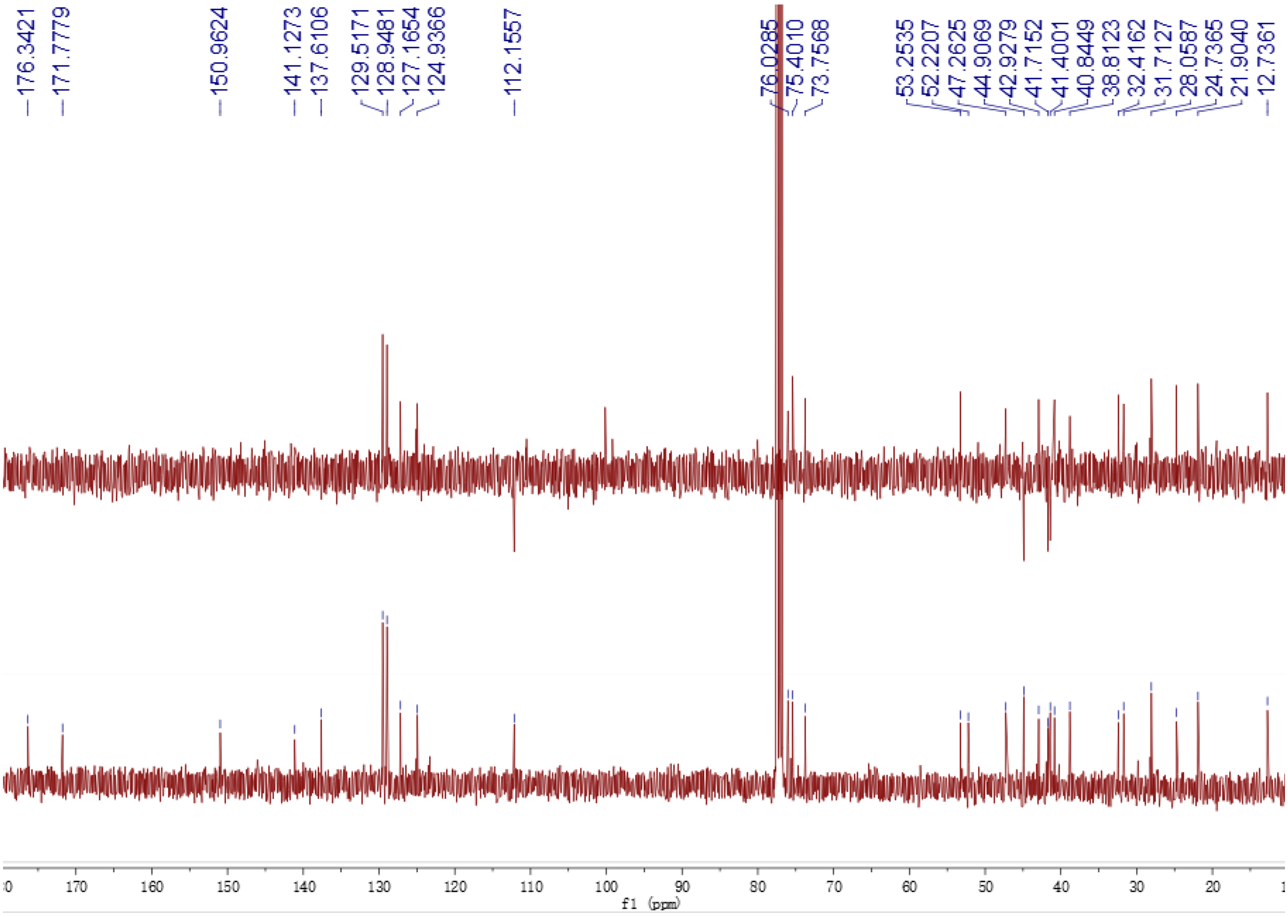

Spectrum S15. HSQC spectrum of **3** in $CDCl_3$

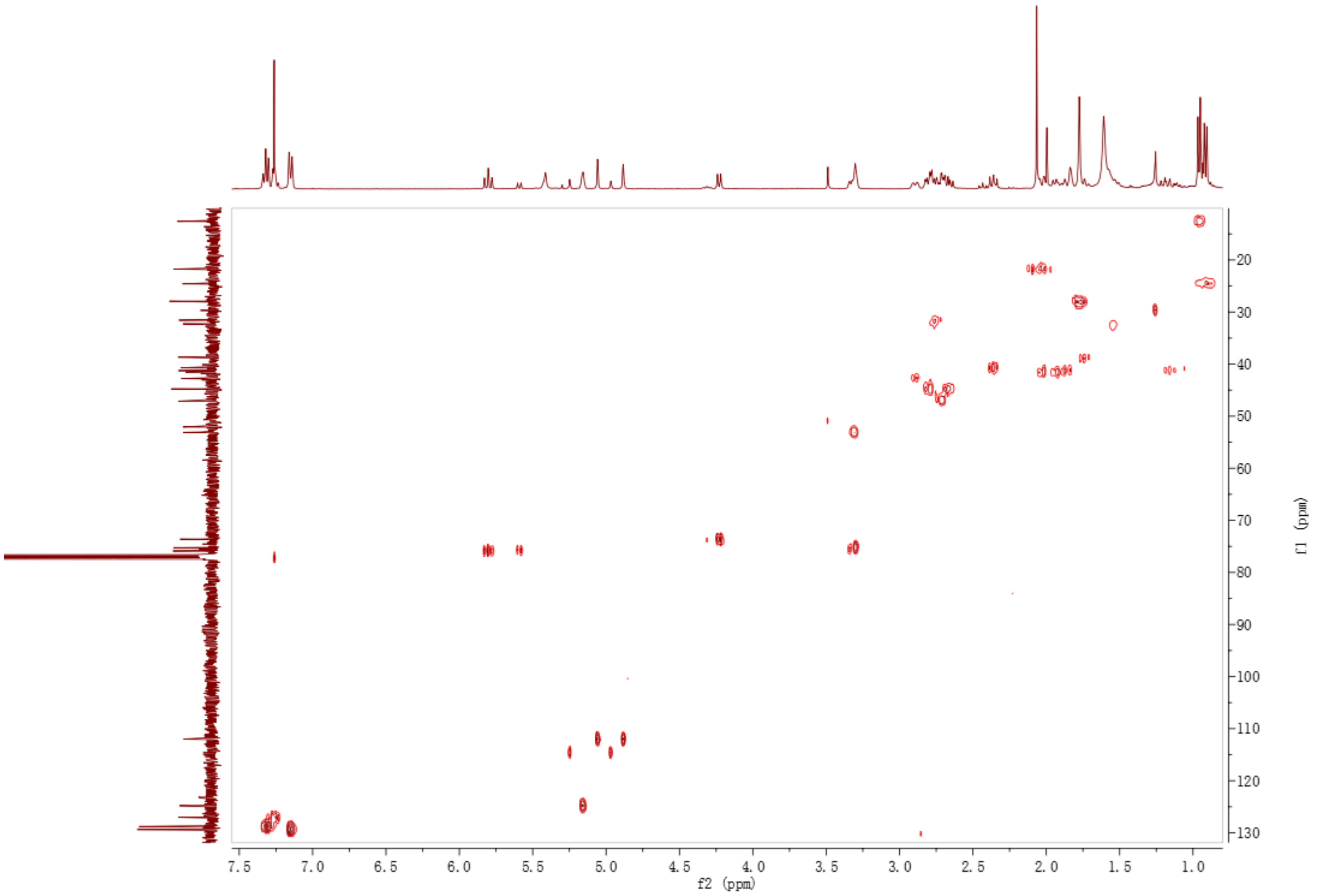

Spectrum S16. ^{1}H–^{1}H COSY spectrum of **3** in $CDCl_3$

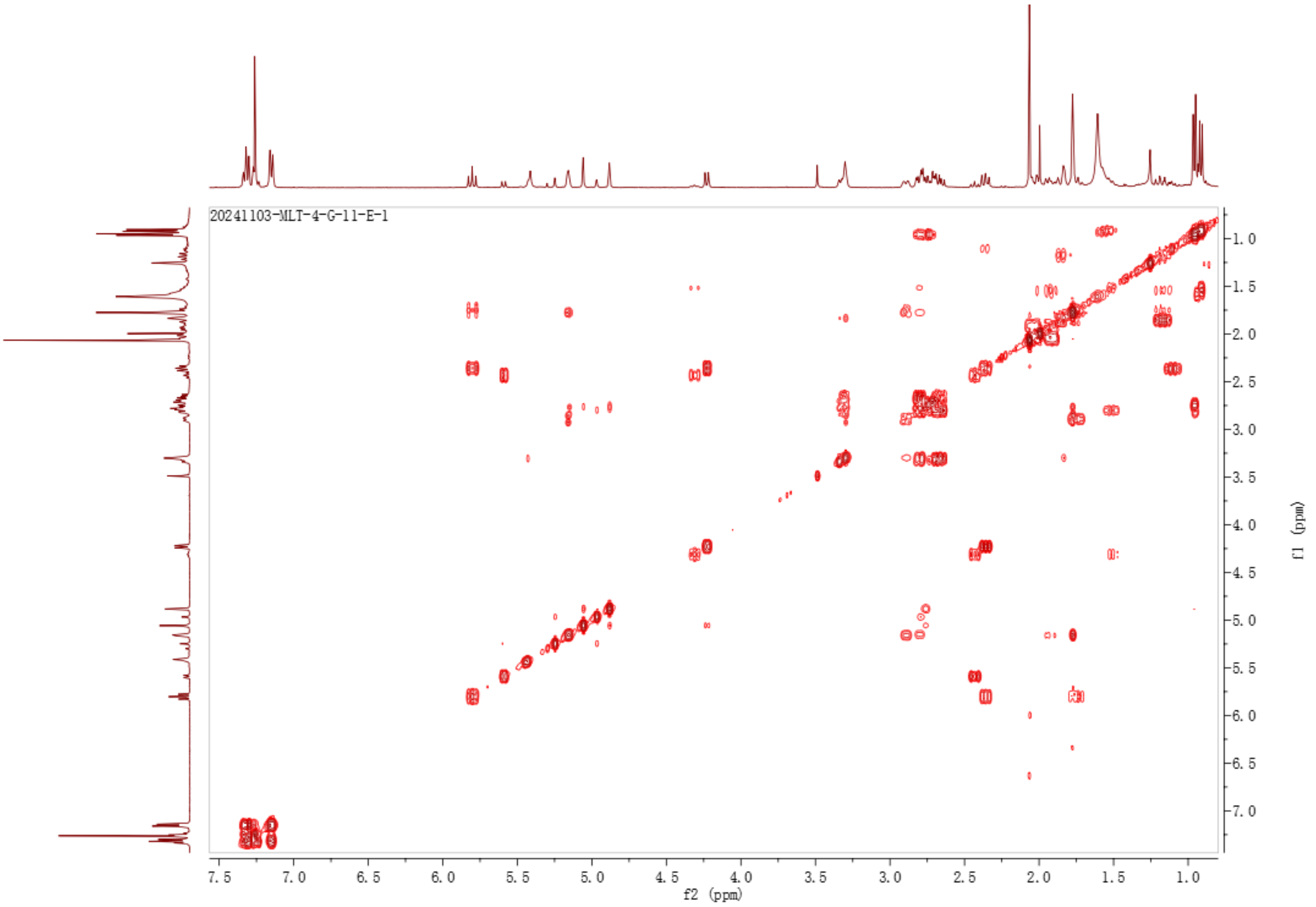

Spectrum S17. HMBC spectrum of **3** in $CDCl_3$

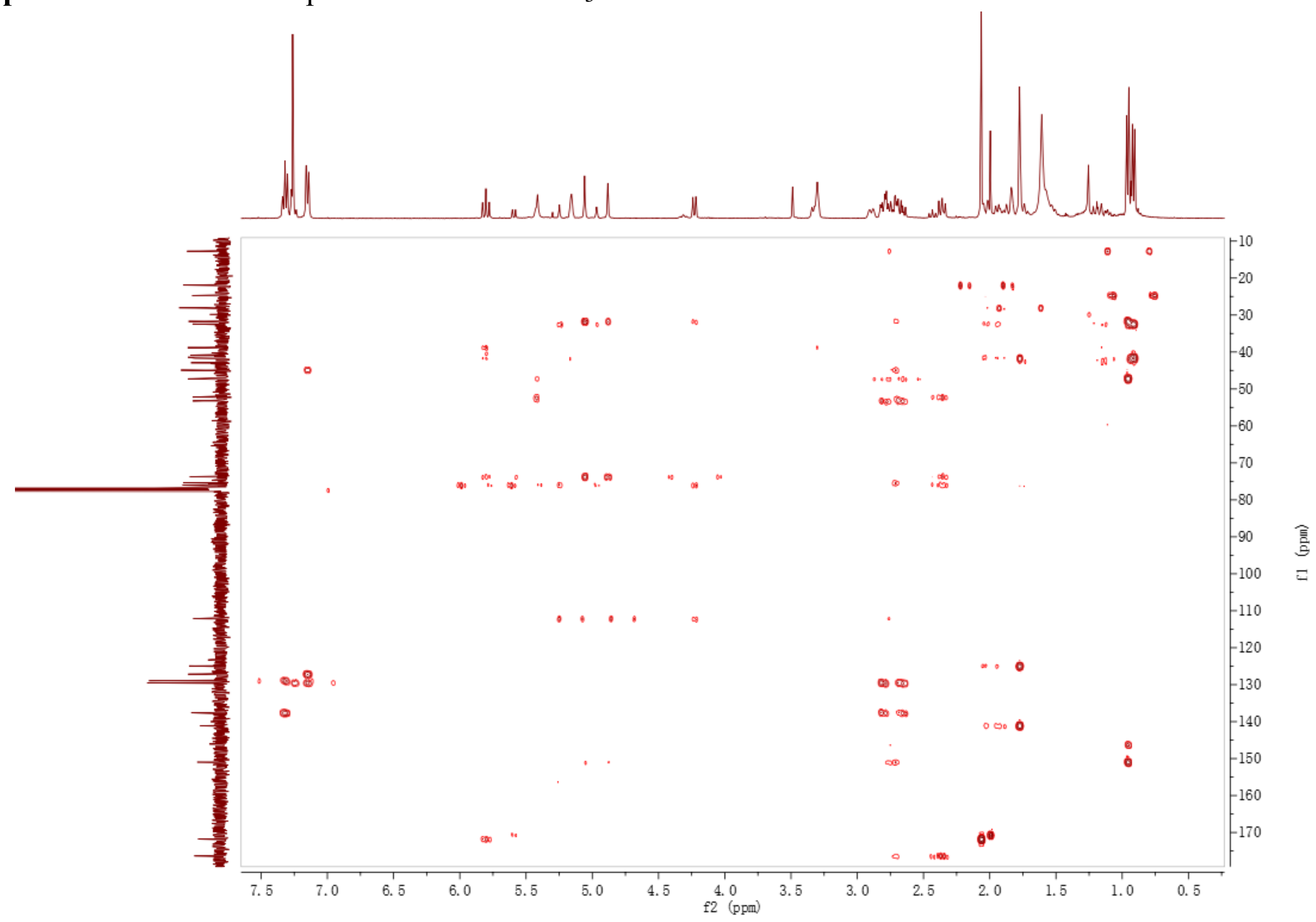

Spectrum S18. NOESY spectrum of **3** in $CDCl_3$

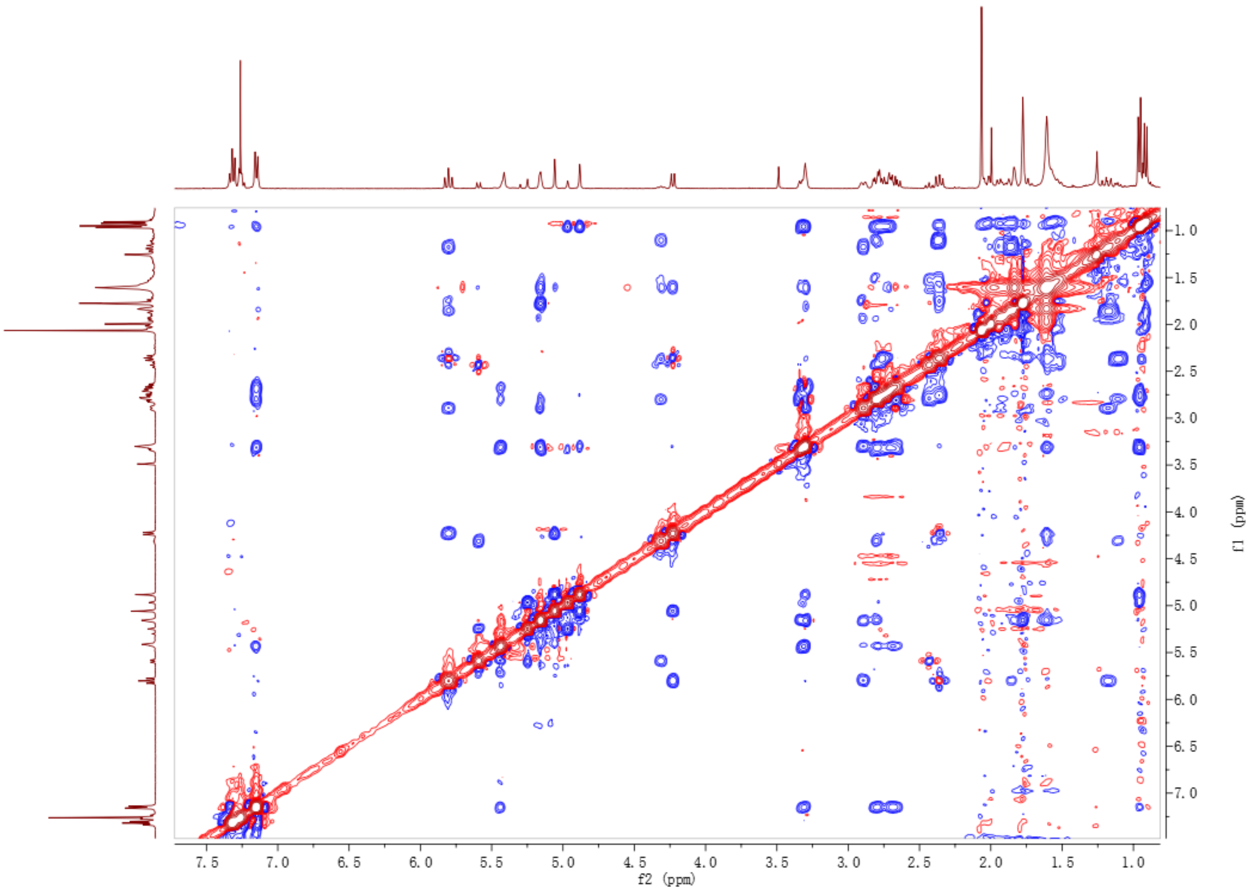

Spectrum S19. ^{1}H NMR spectrum of **4** in $CDCl_3$

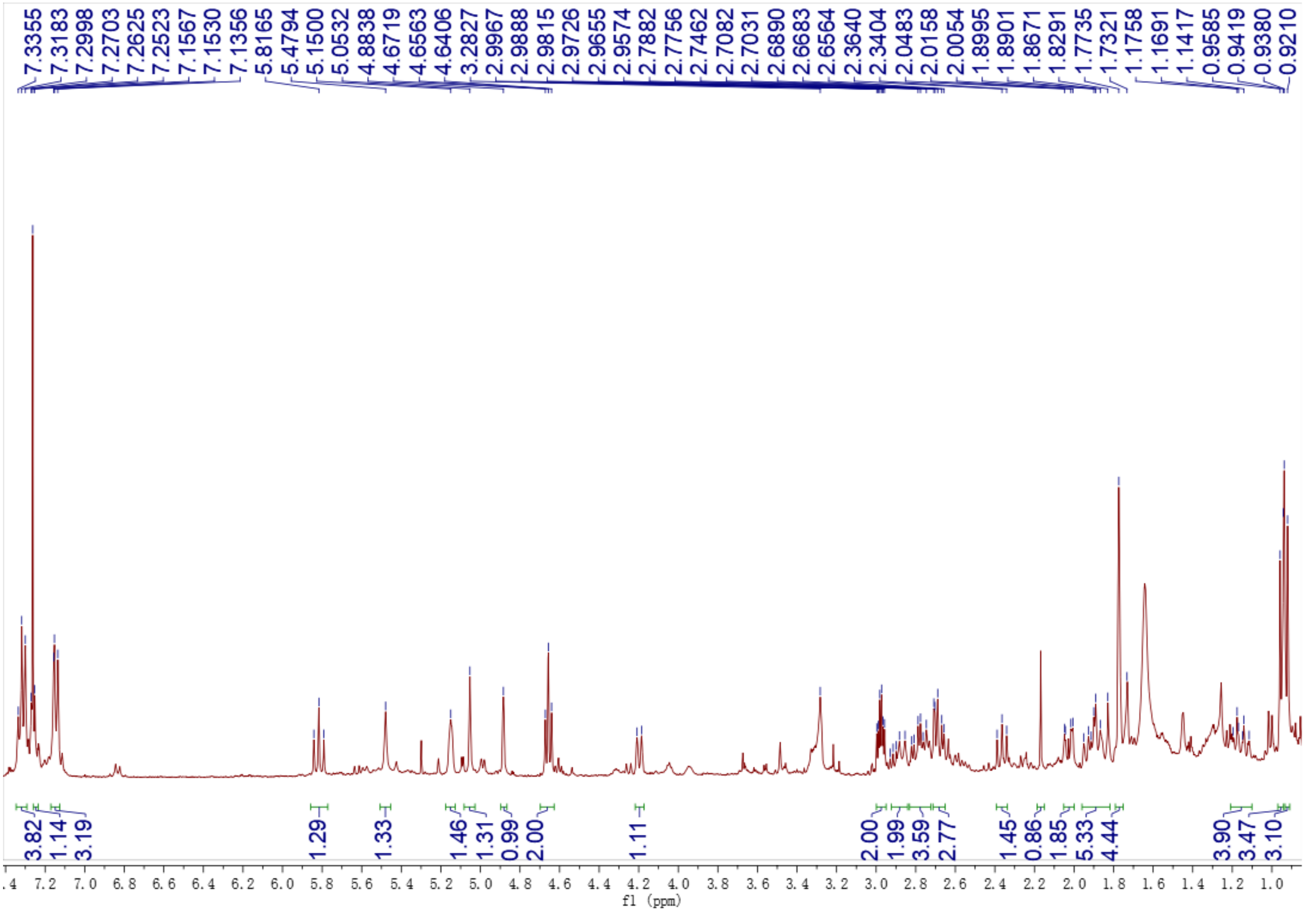

Spectrum S20. ^{13}C NMR and DEPT spectra of **4** in $CDCl_3$

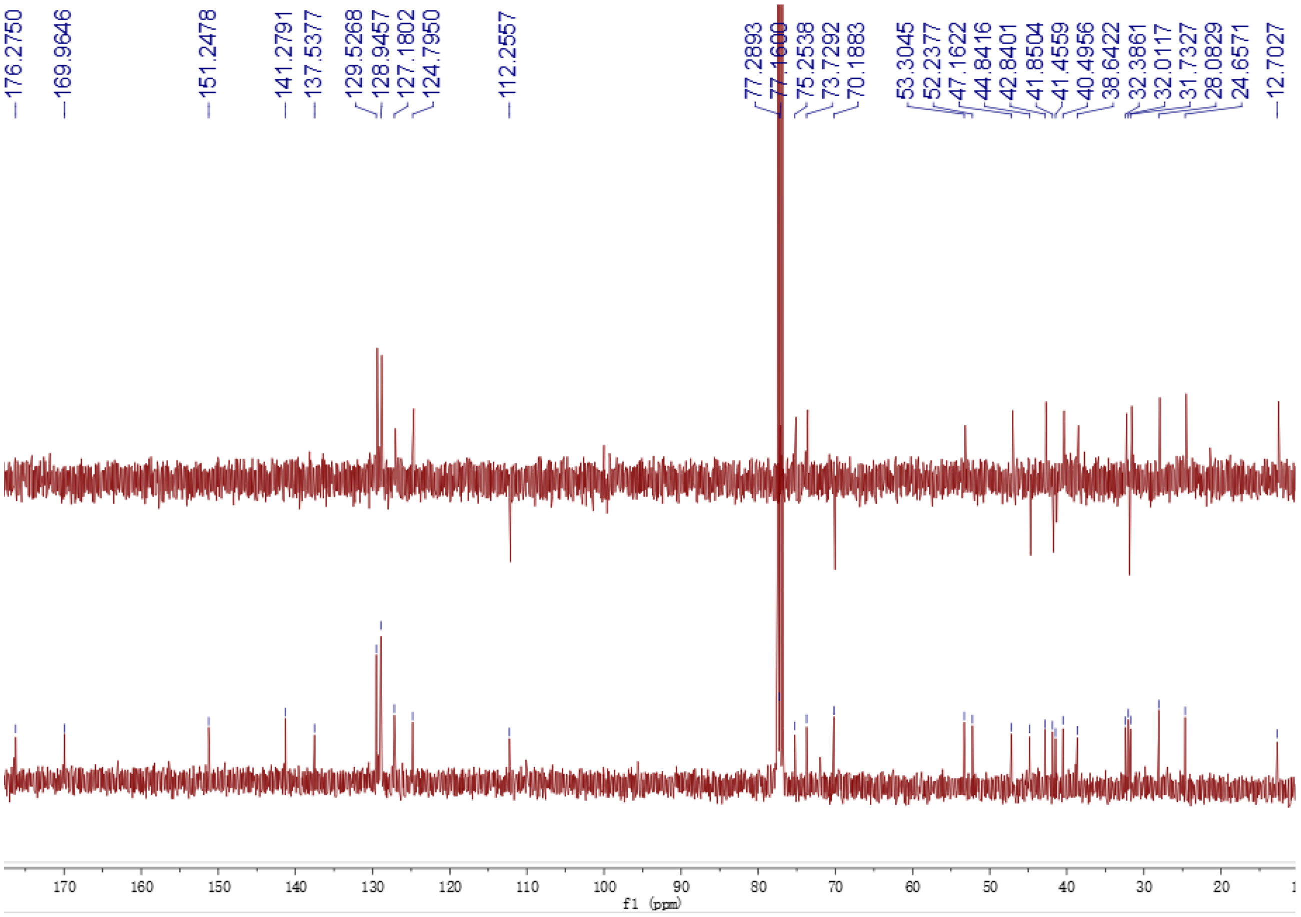

Spectrum S21. HSQC spectrum of **4** in $CDCl_3$

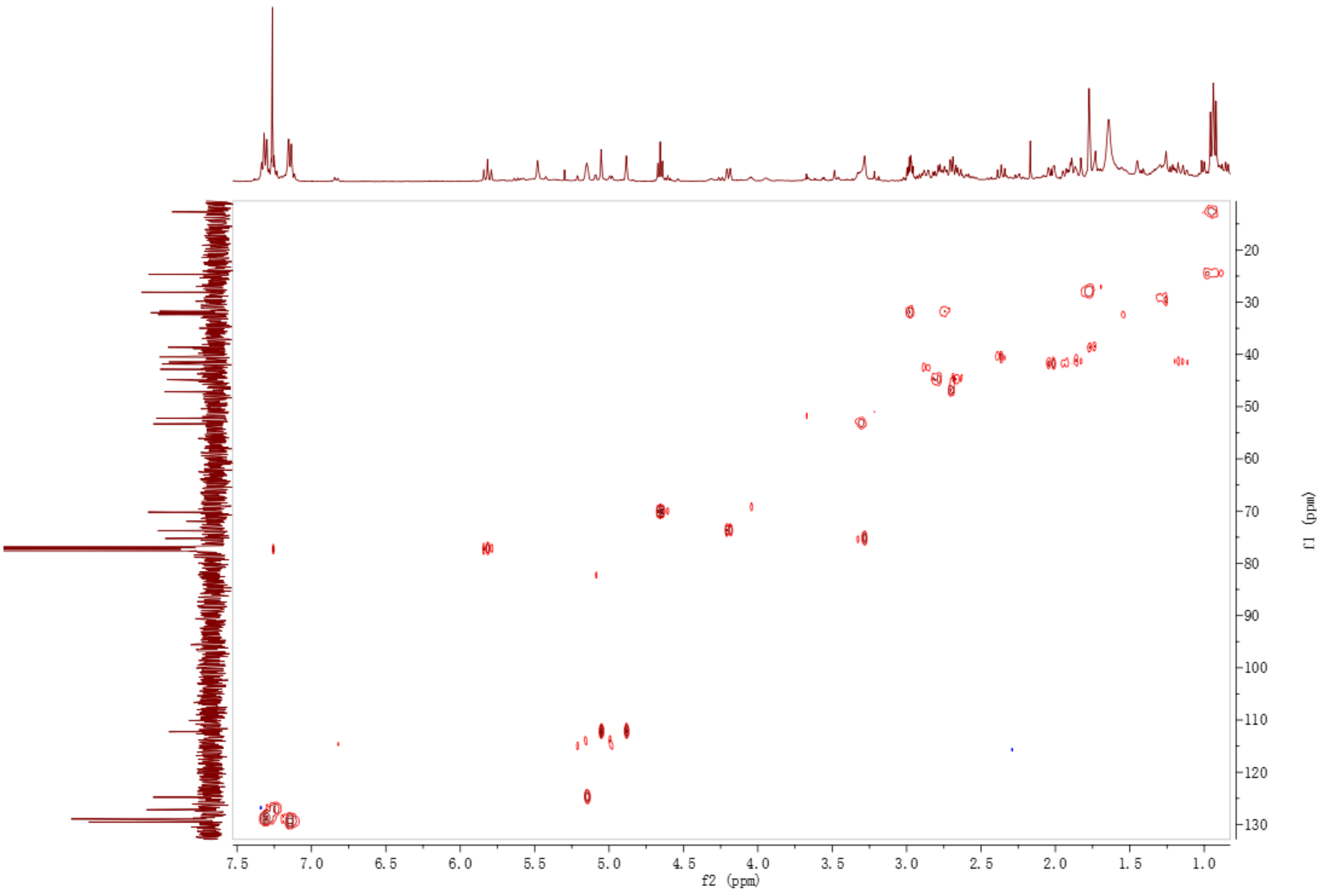

Spectrum S22. ^1H–^1H COSY spectrum of **4** in $CDCl_3$

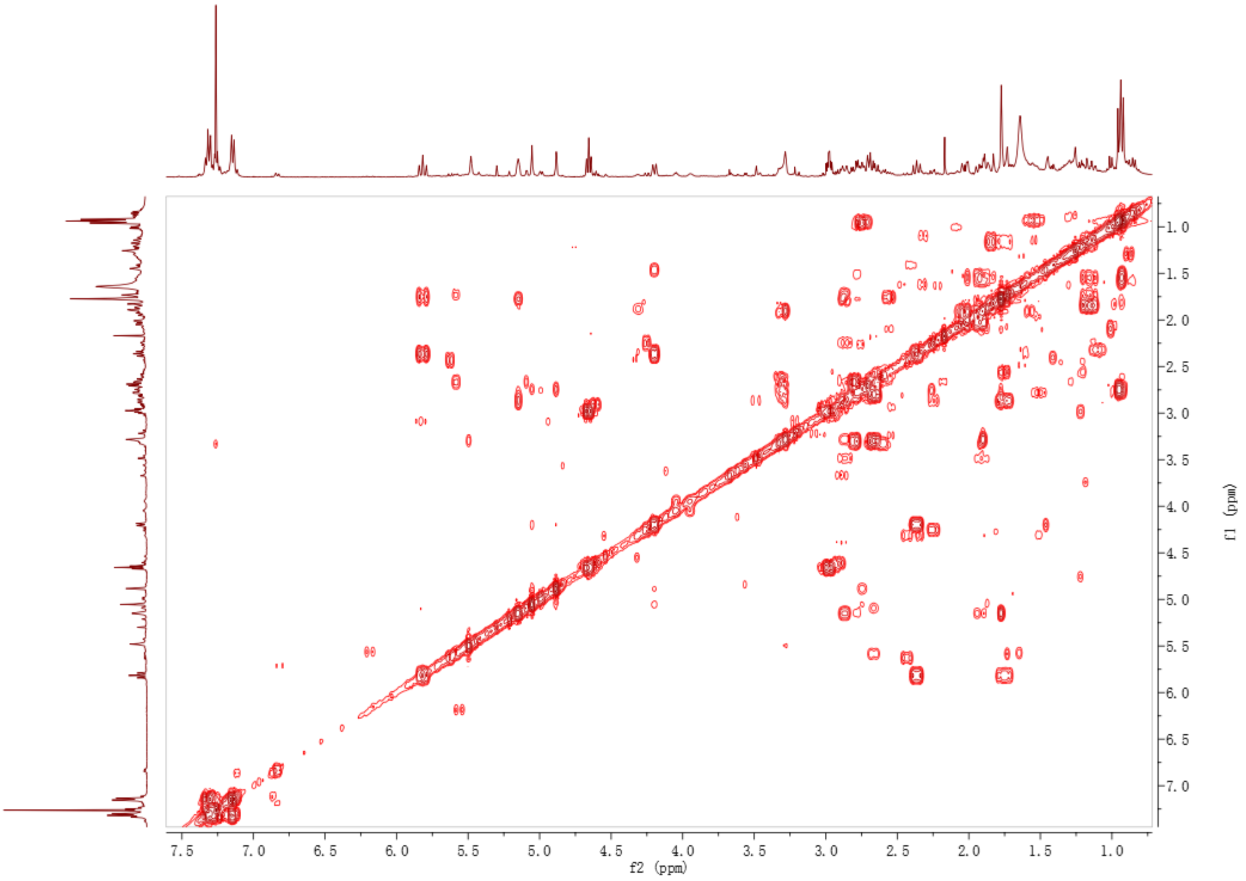

Spectrum S23. HMBC spectrum of **4** in $CDCl_3$

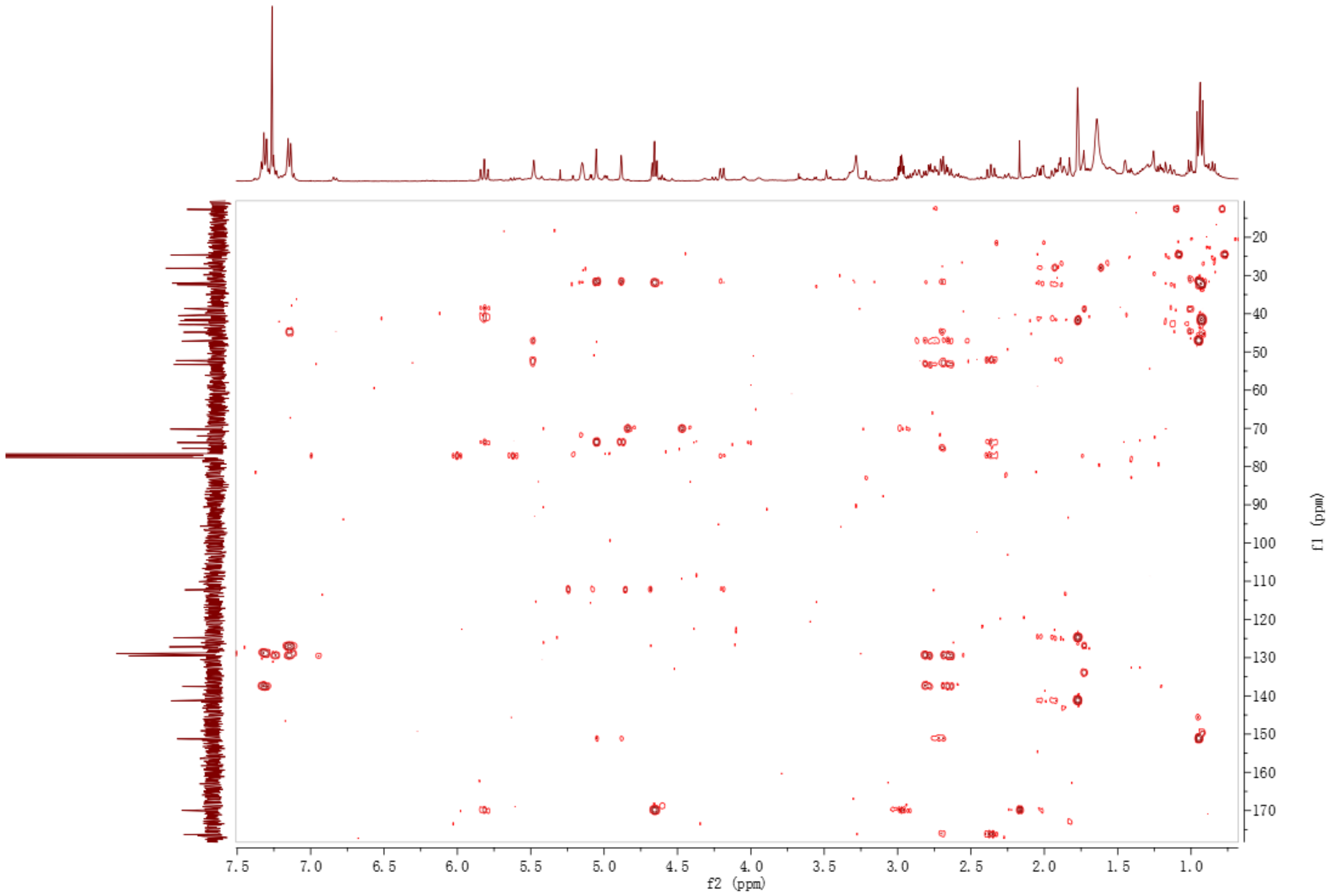

Spectrum S24. NOESY spectrum of **4** in $CDCl_3$

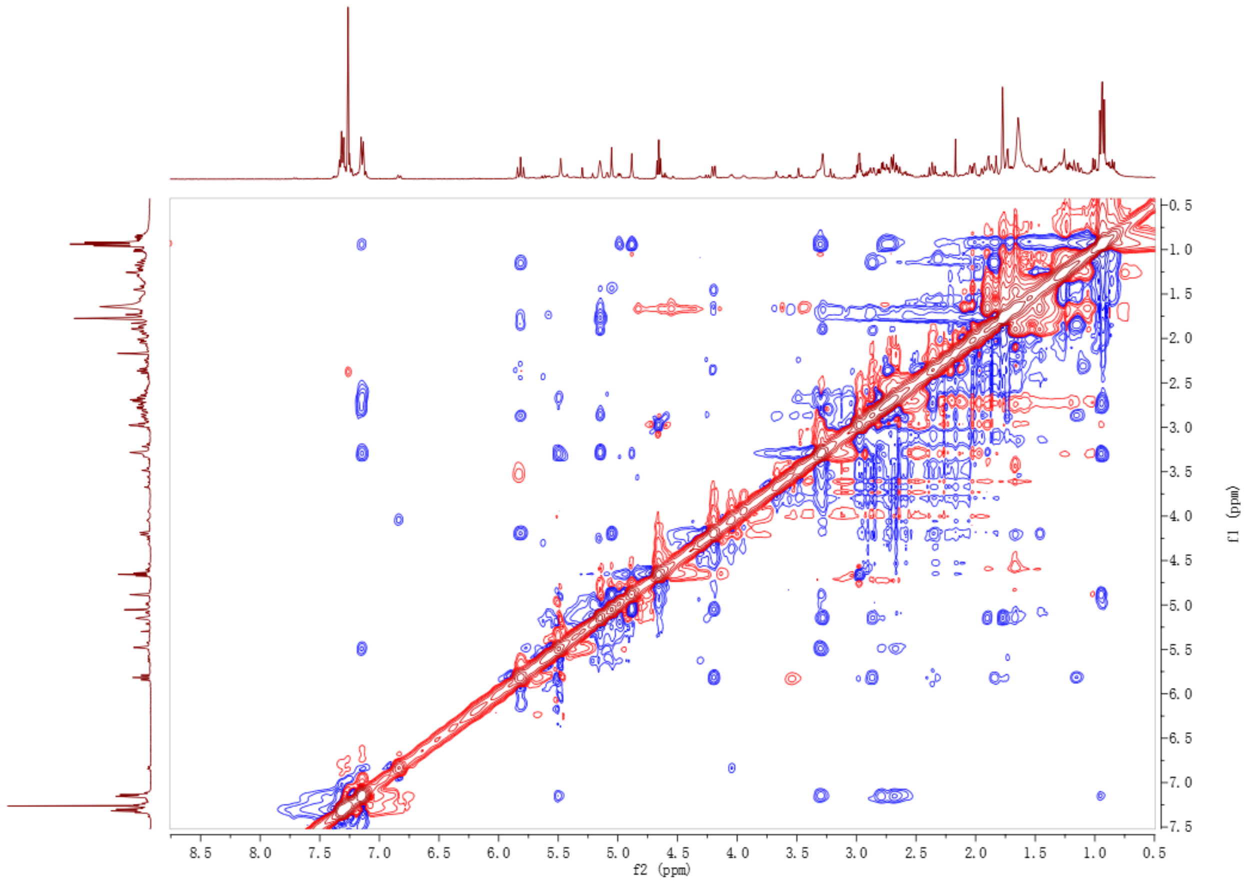

Spectrum S25. ^{1}H NMR spectrum of **5** in $CDCl_3$

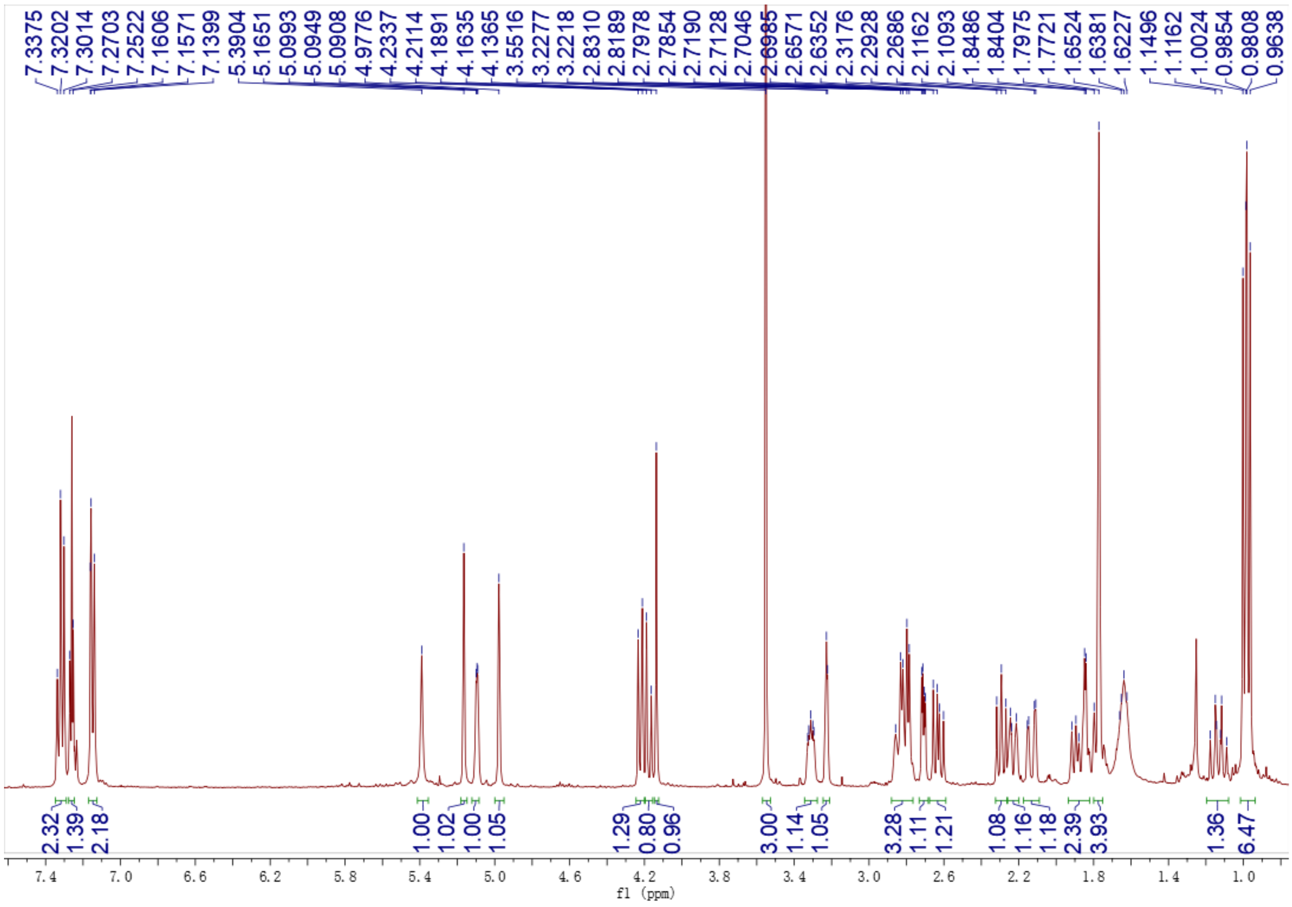

Spectrum S26. ^{13}C NMR and DEPT spectra of **5** in $CDCl_3$

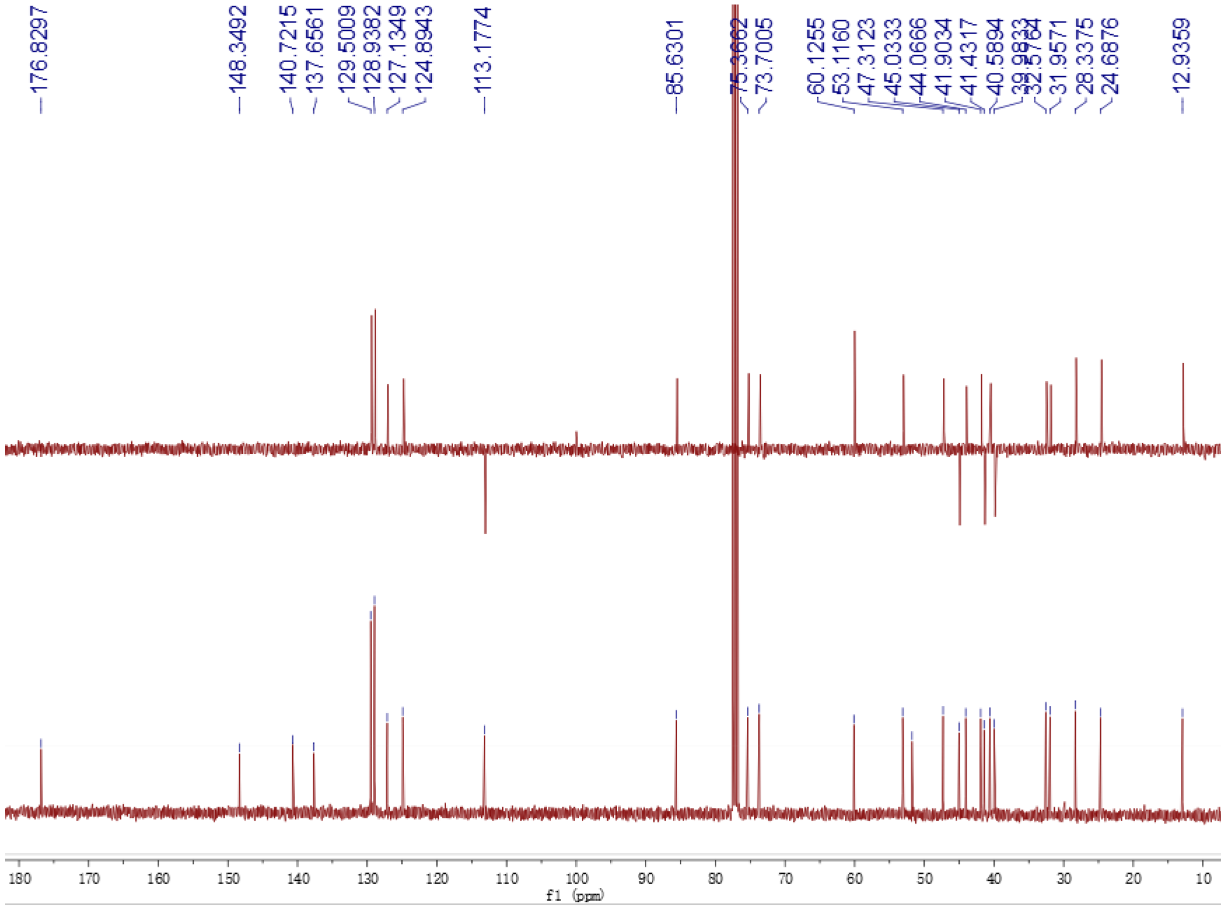

Spectrum S27. HSQC spectrum of **5** in $CDCl_3$

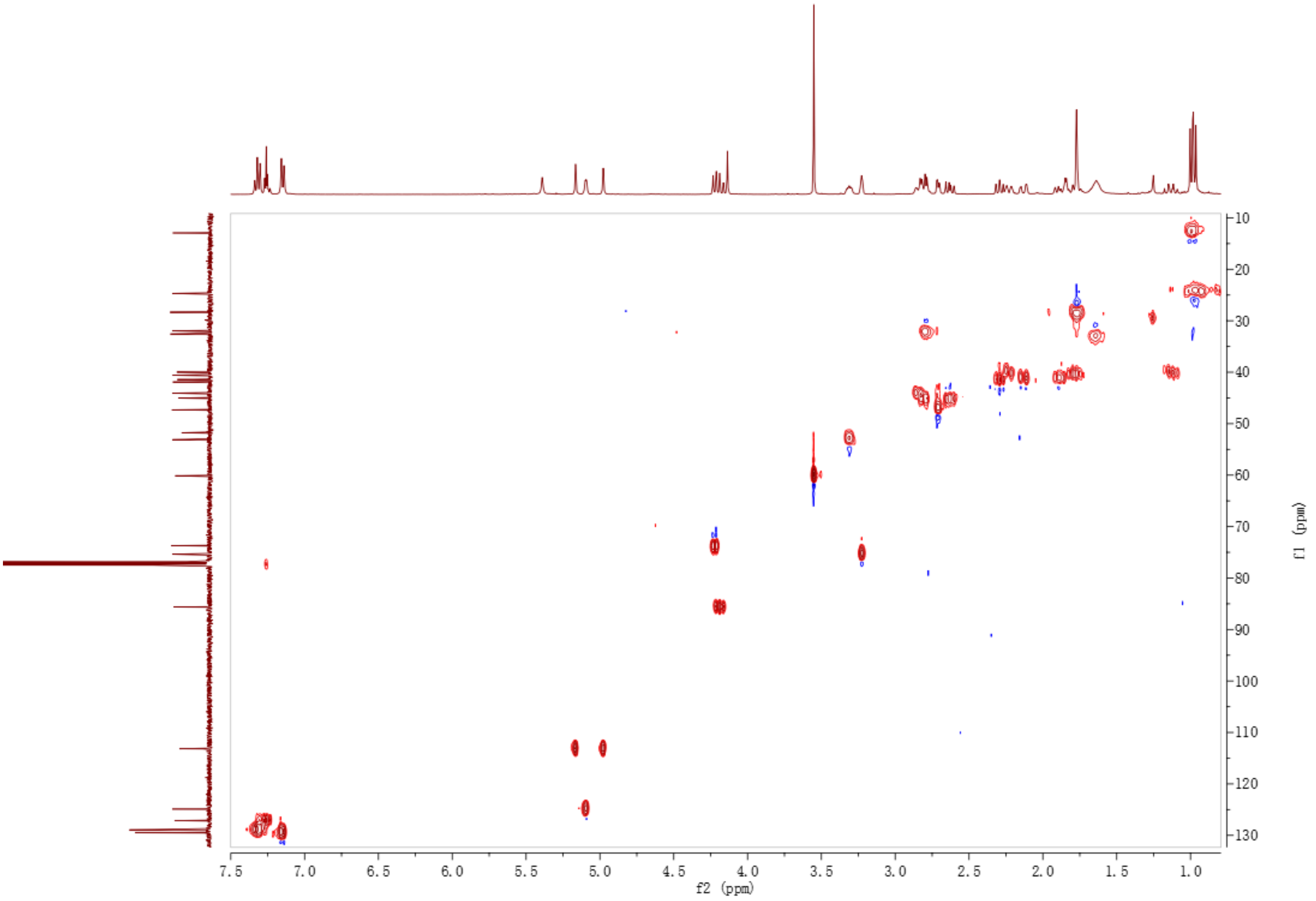

Spectrum S28. ^1H–^1H COSY spectrum of **5** in $CDCl_3$

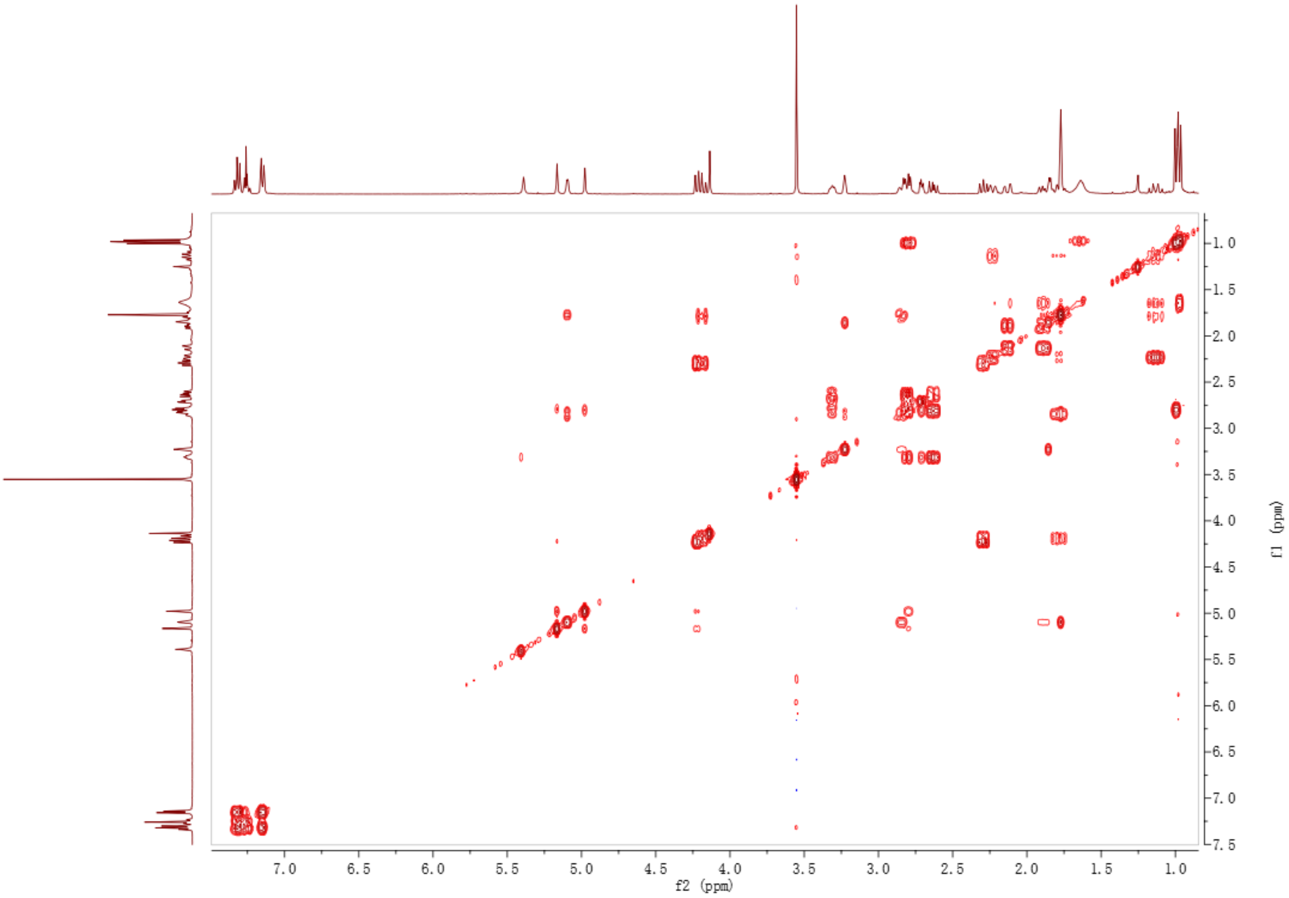

Spectrum S29. HMBC spectrum of **5** in $CDCl_3$

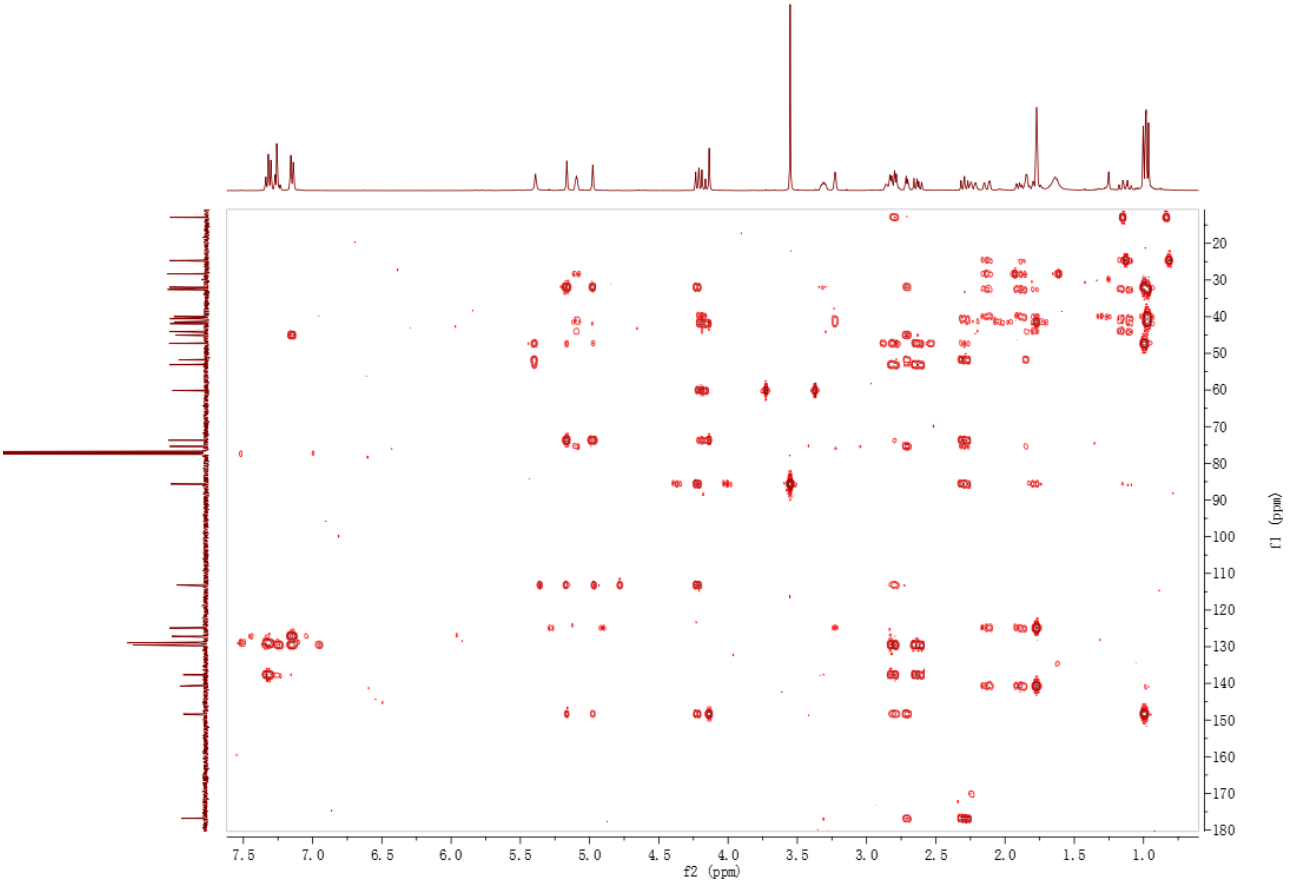

Spectrum S30. NOESY spectrum of **5** in $CDCl_3$

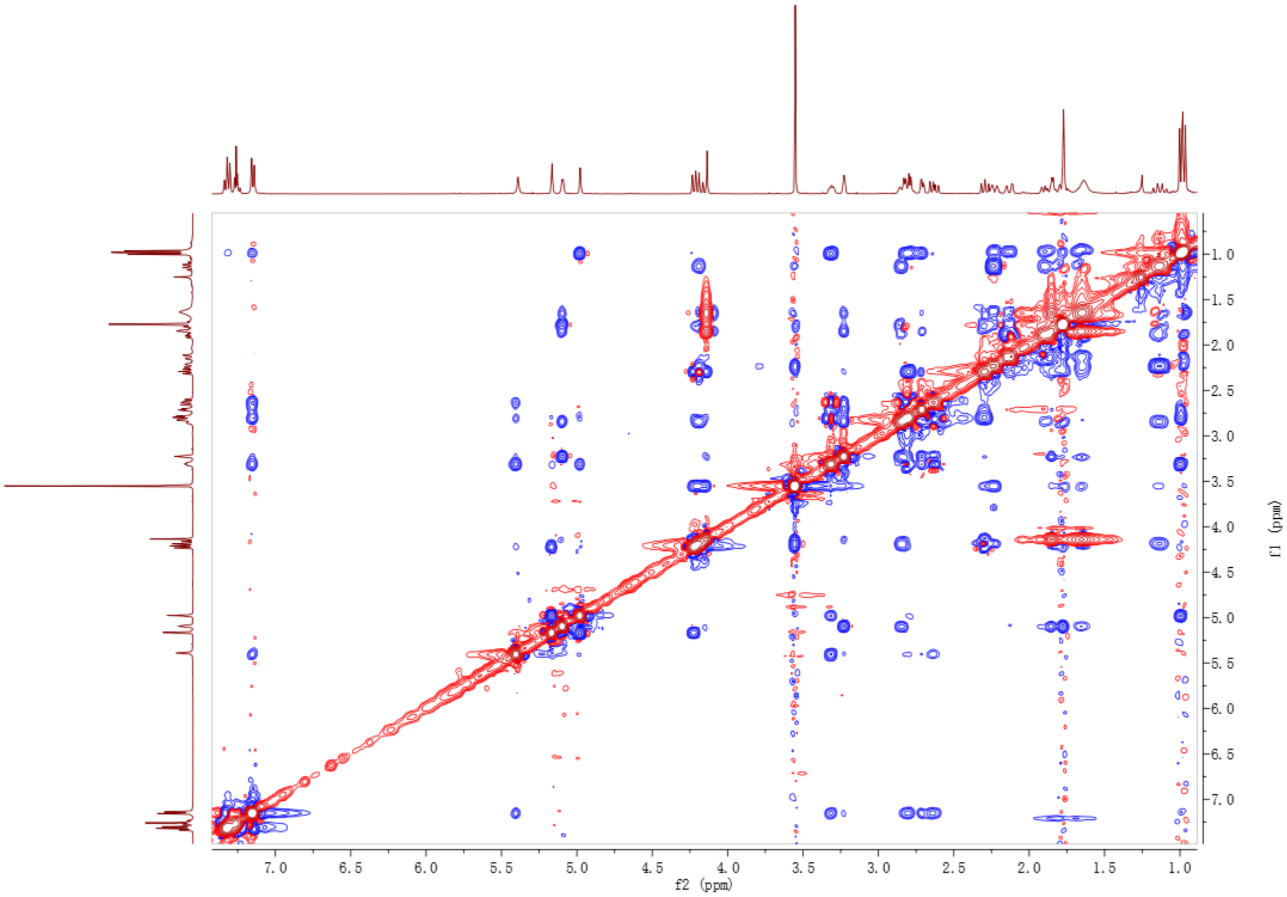

Spectrum S31. ^{1}H NMR spectrum of **6** in $CDCl_3$

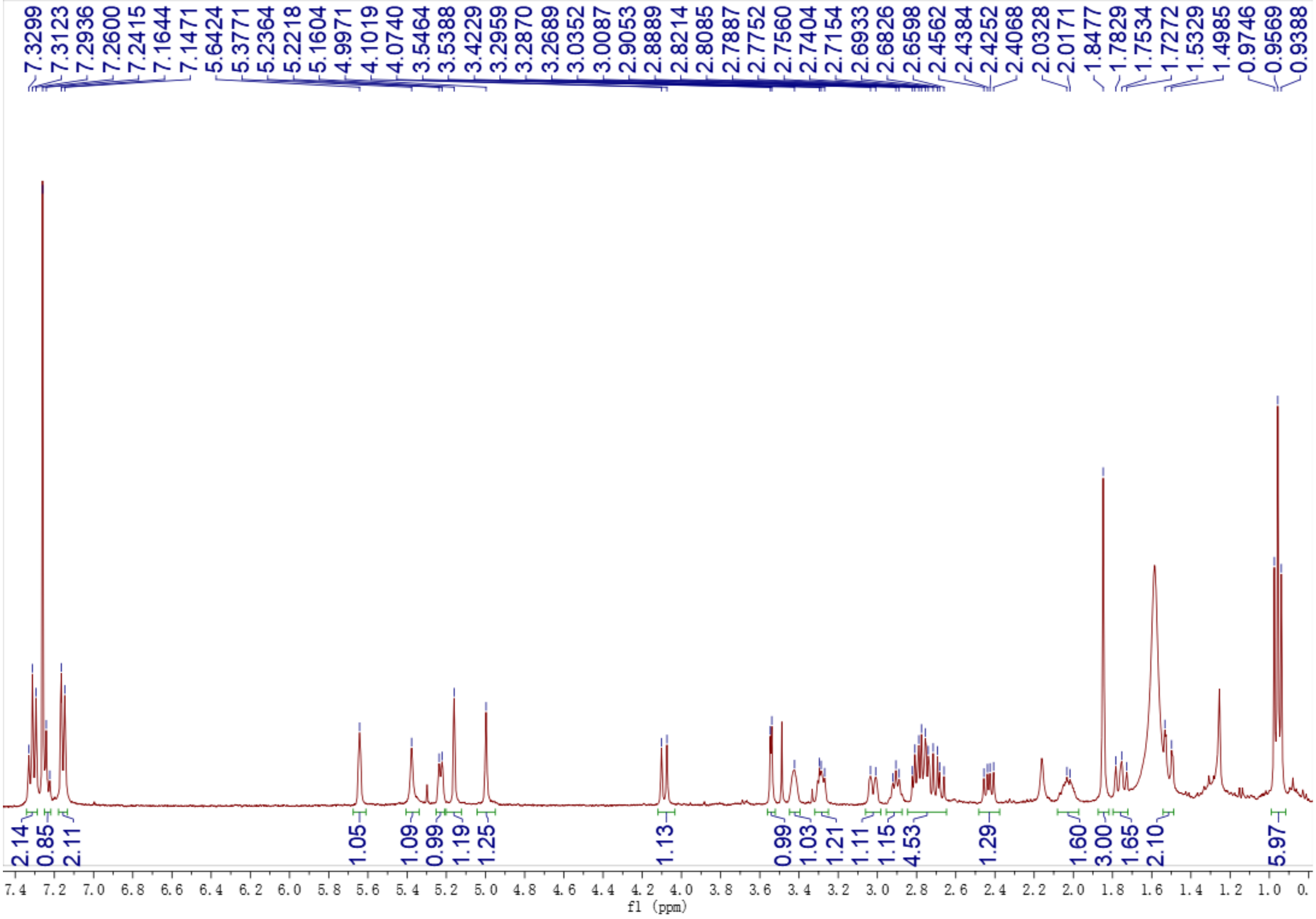

Spectrum S32. ^{13}C NMR and DEPT spectra of **6** in $CDCl_3$

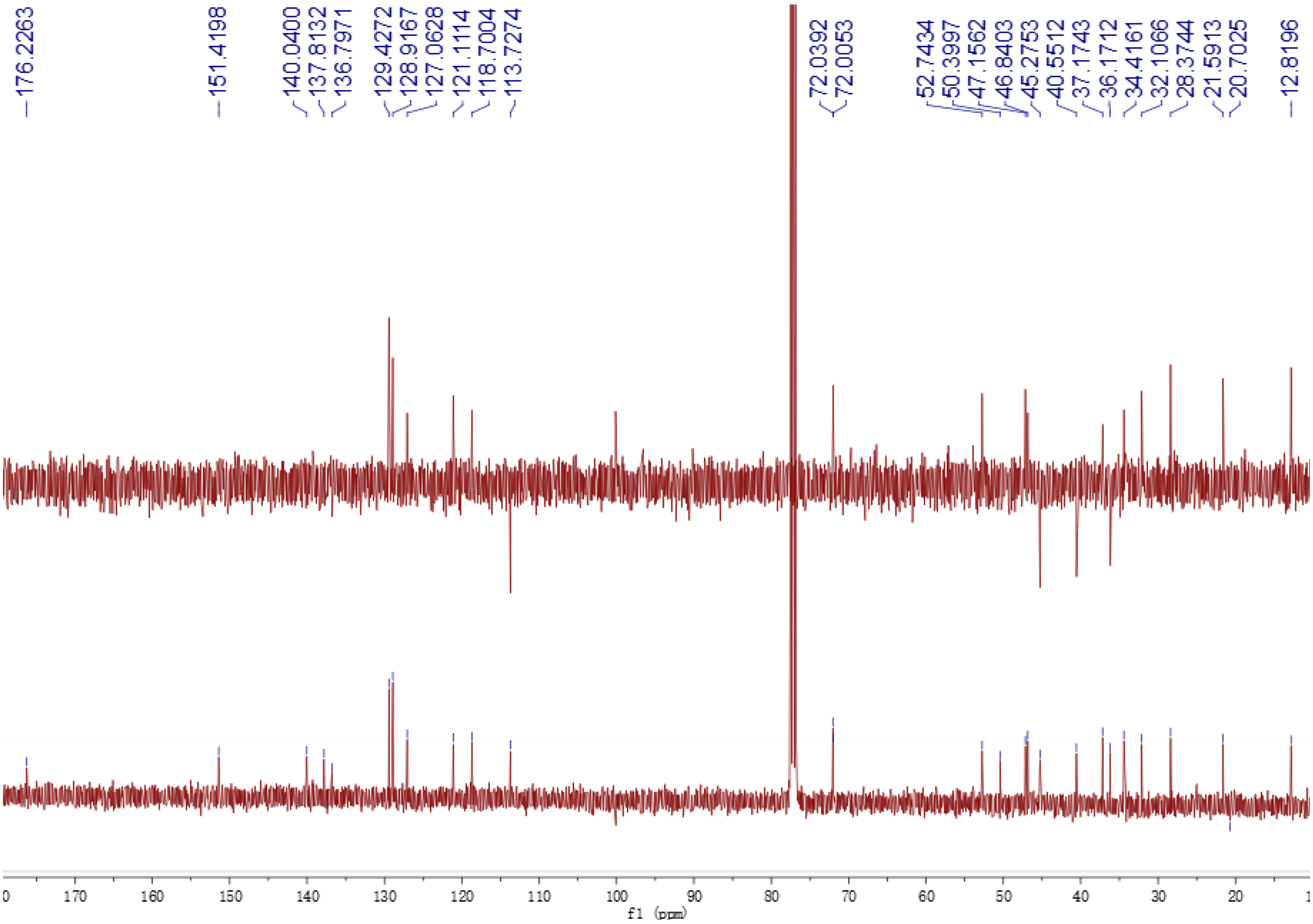

Spectrum S33. HSQC spectrum of **6** in $CDCl_3$

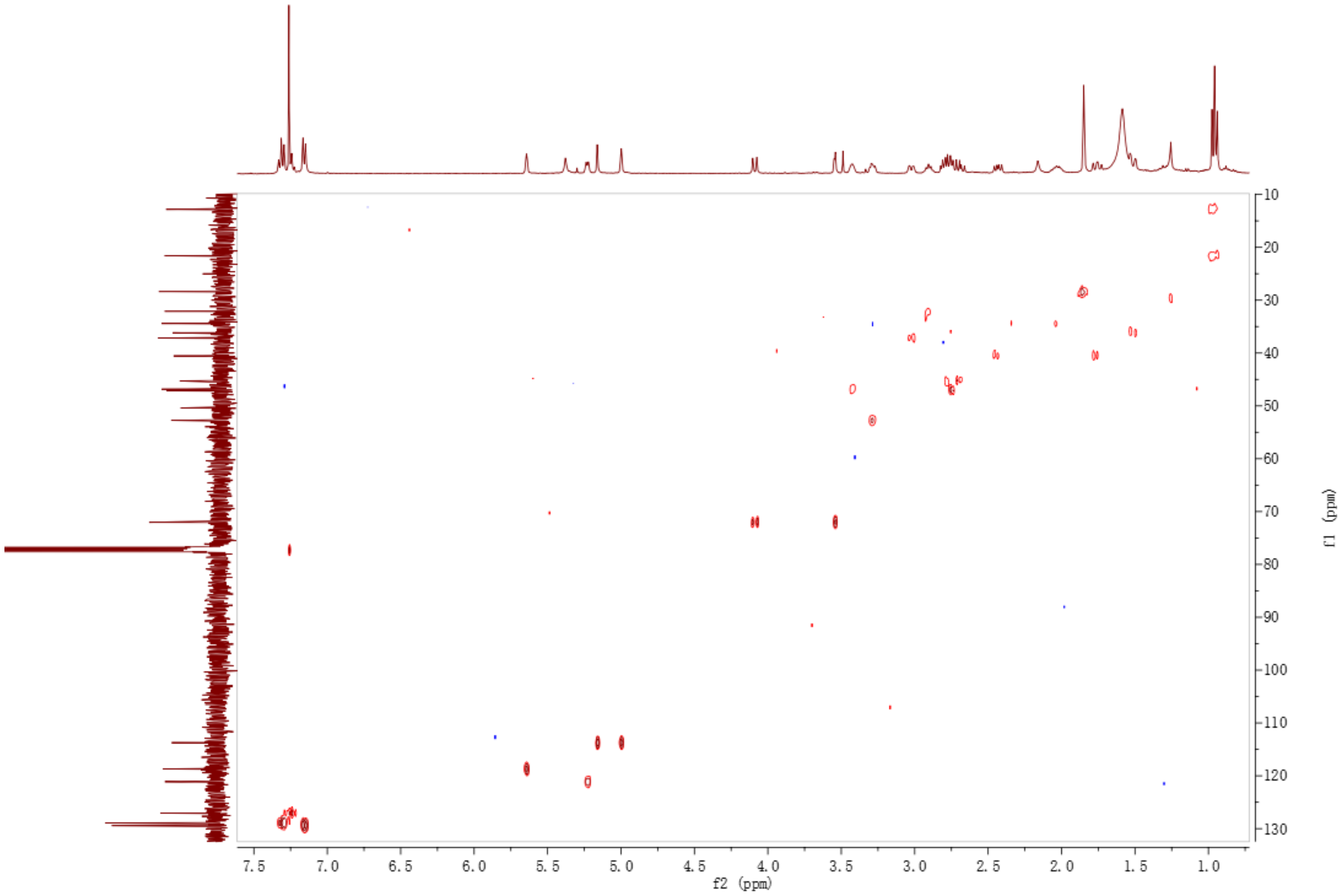

Spectrum S34. ^{1}H–^{1}H COSY spectrum of **6** in $CDCl_3$

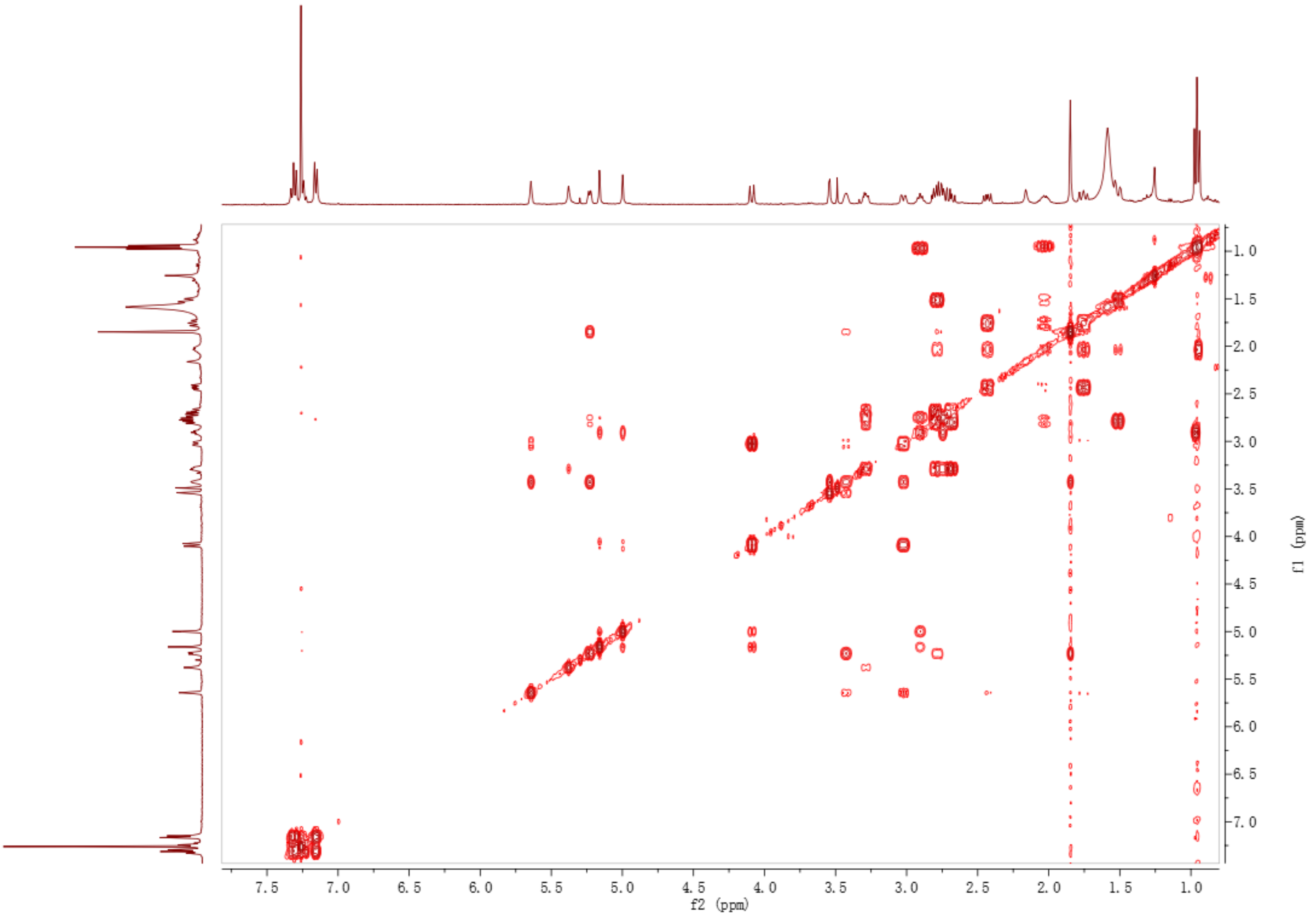

Spectrum S35. HMBC spectrum of **6** in $CDCl_3$

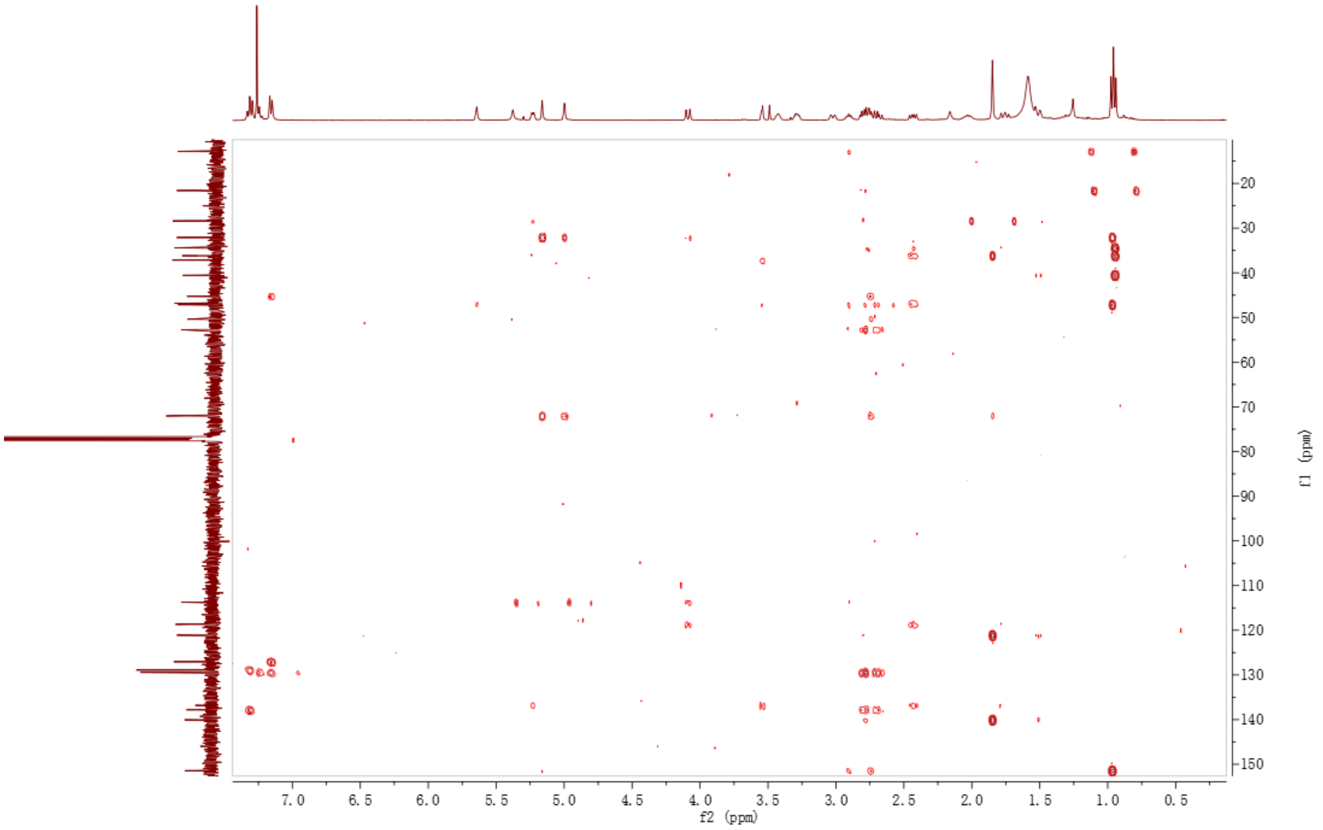

Spectrum S36. NOESY spectrum of **6** in $CDCl_3$

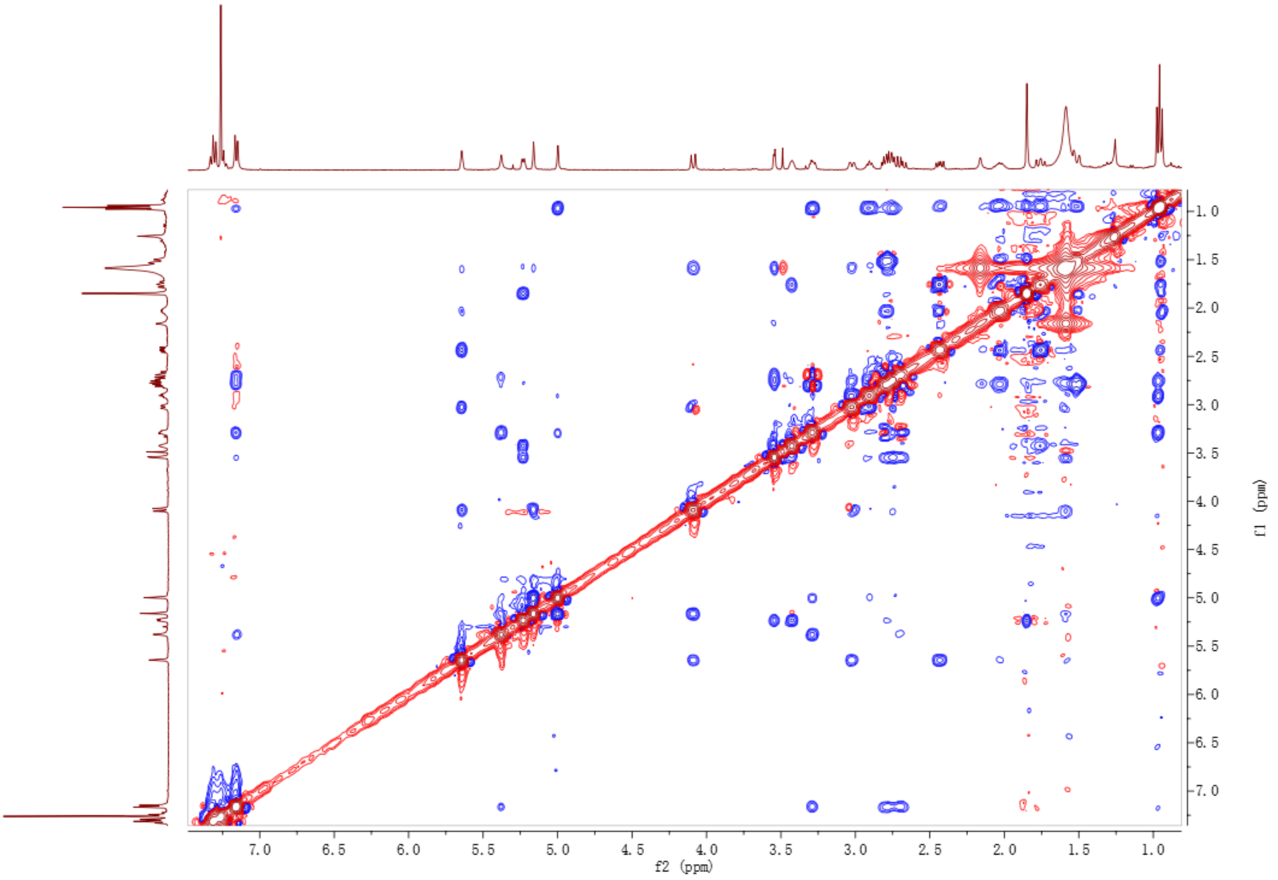

Spectrum S37. ^{1}H NMR spectrum of **7** in $CDCl_3$

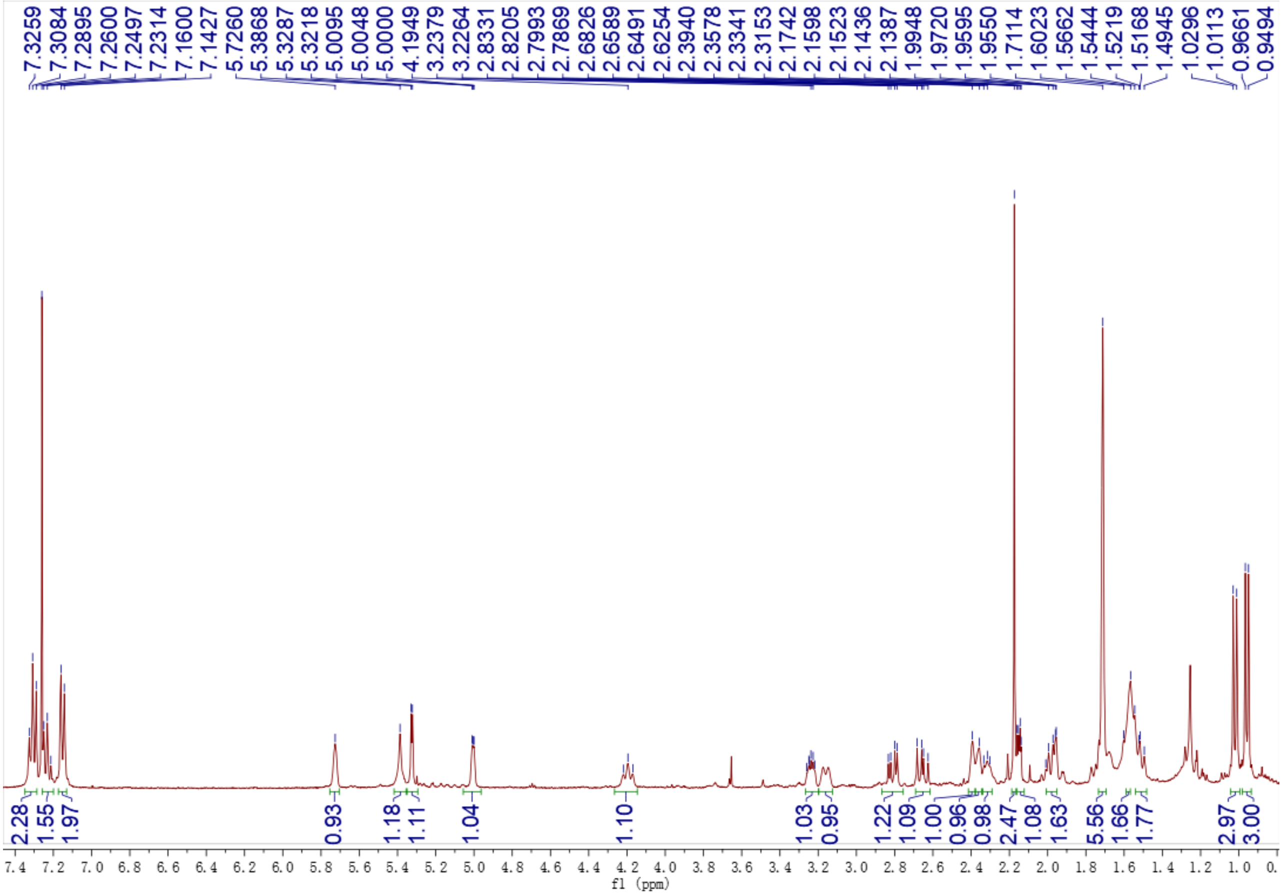

Spectrum S38. ^{13}C NMR and DEPT spectra of **7** in $CDCl_3$

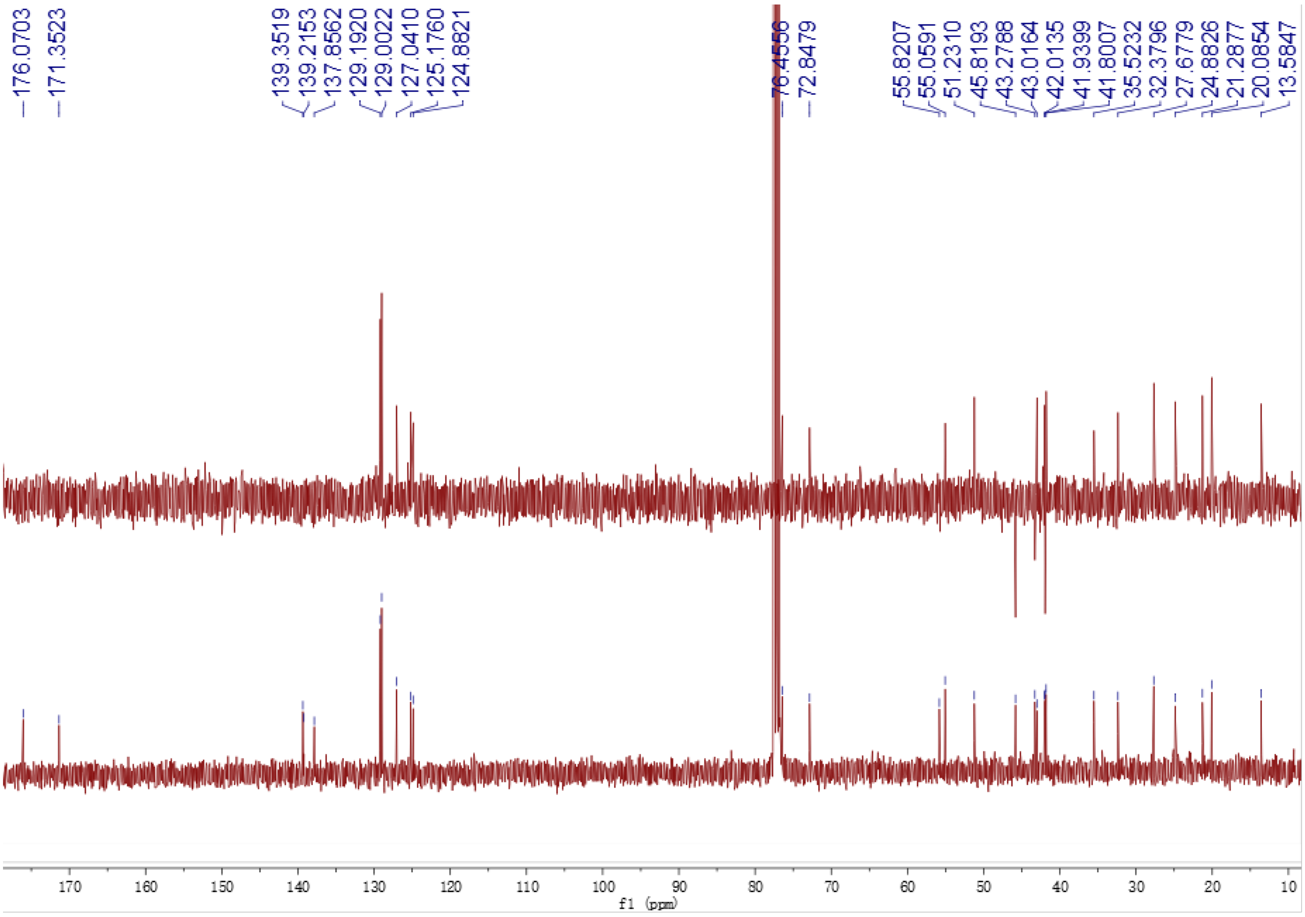

Spectrum S39. HSQC spectrum of **7** in $CDCl_3$

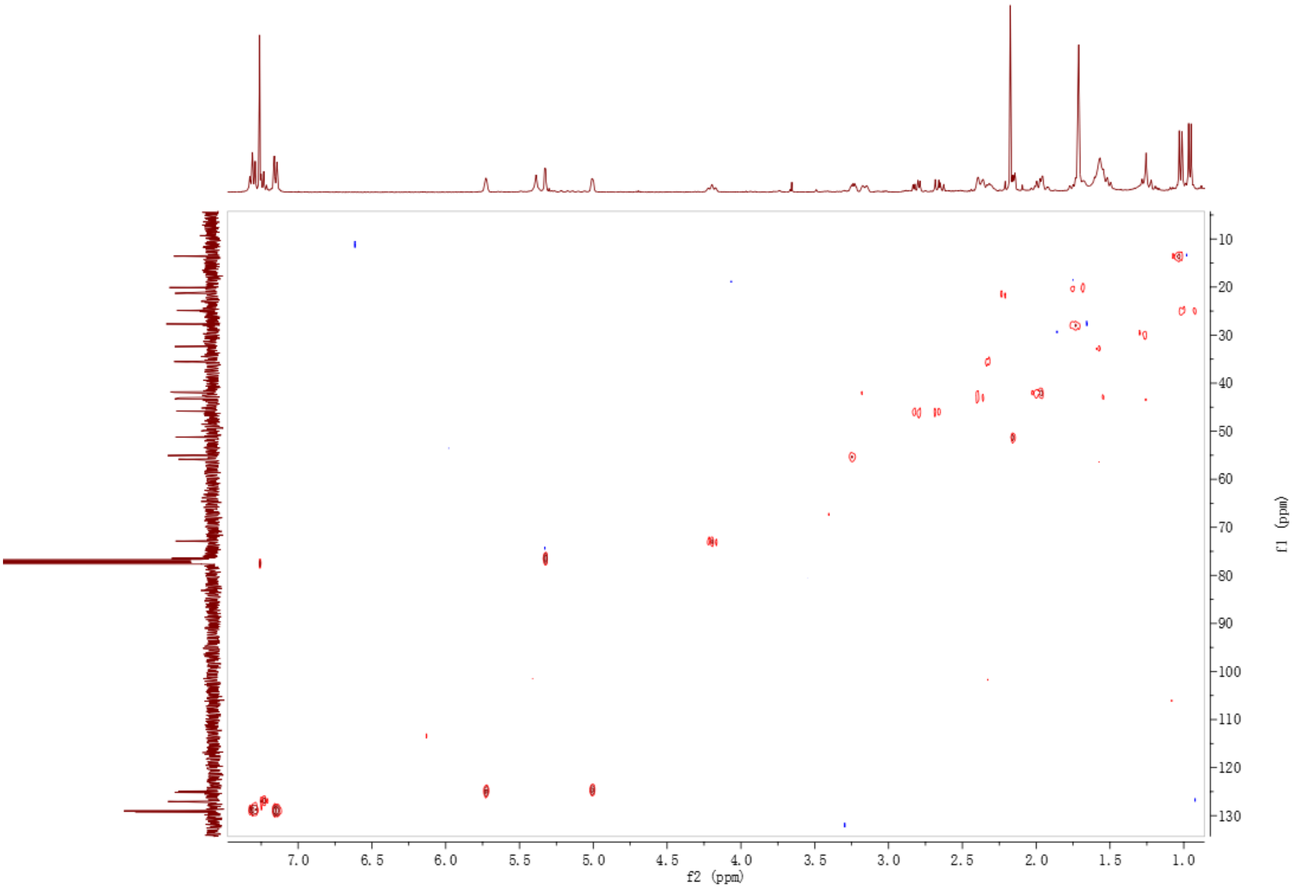

Spectrum S40. ^1H–^1H COSY spectrum of **7** in $CDCl_3$

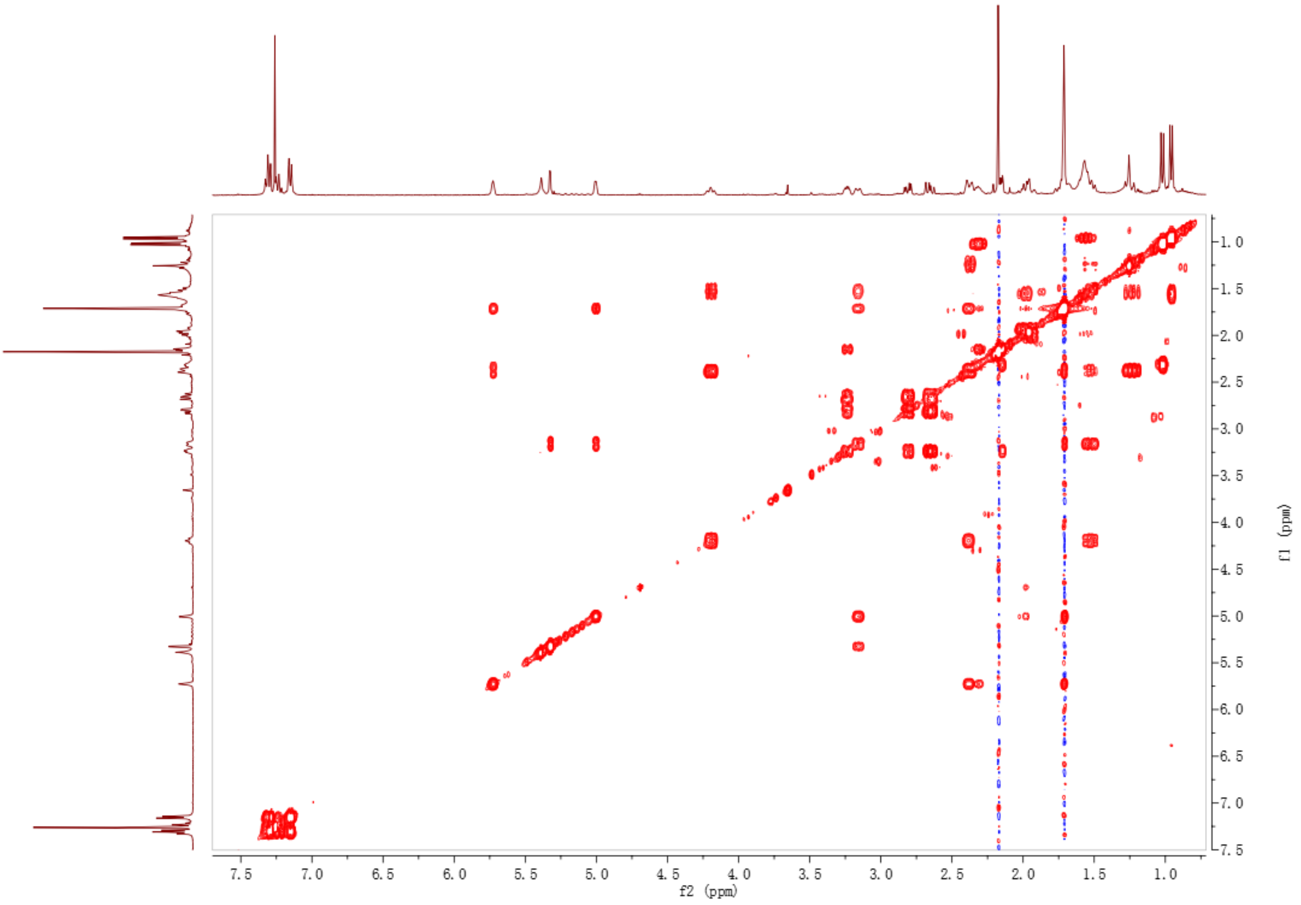

Spectrum S41. HMBC spectrum of **7** in $CDCl_3$

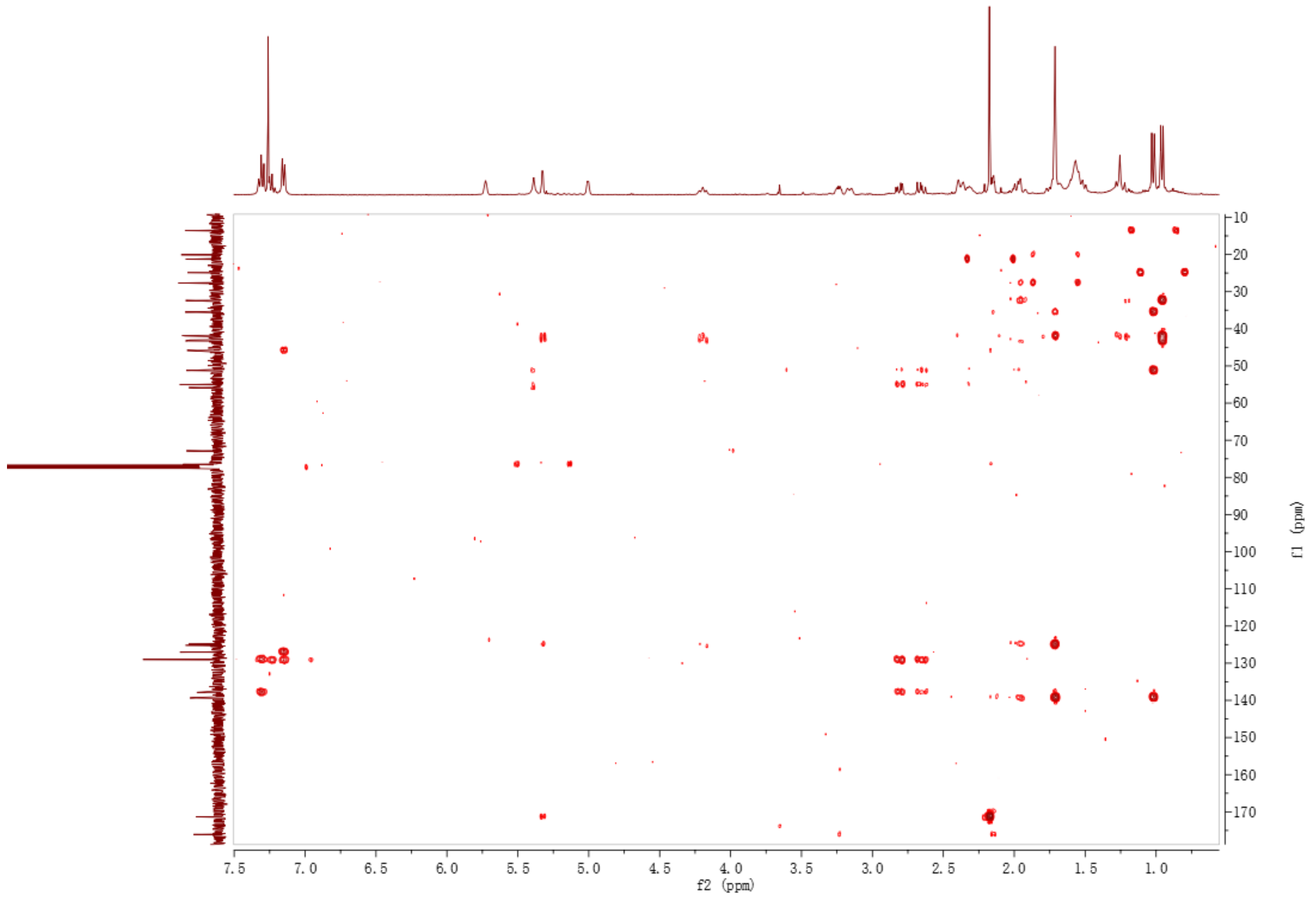

Spectrum S42. NOESY spectrum of **7** in $CDCl_3$

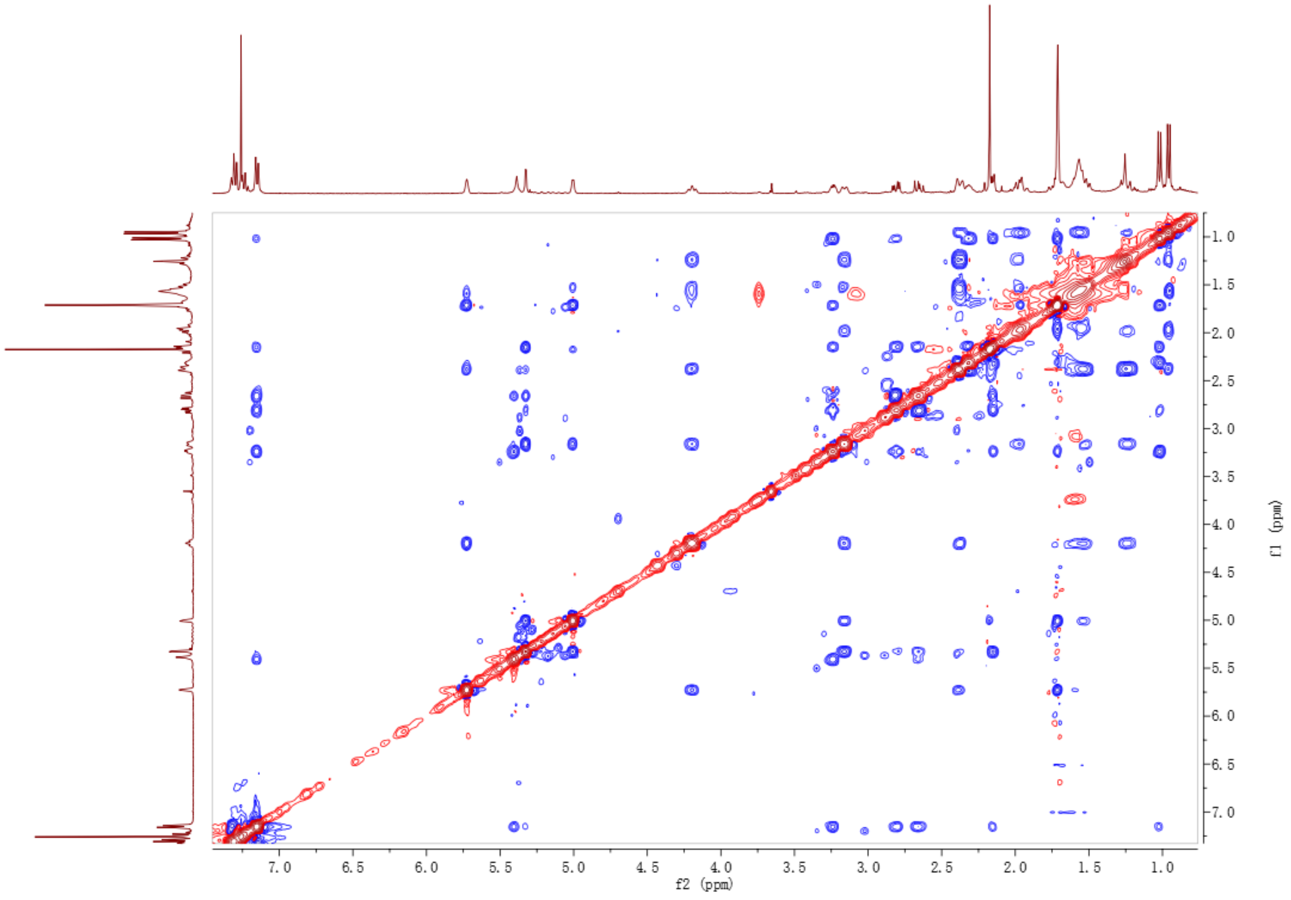

Spectrum S43. ^{1}H NMR spectrum of **8** in $CDCl_3$

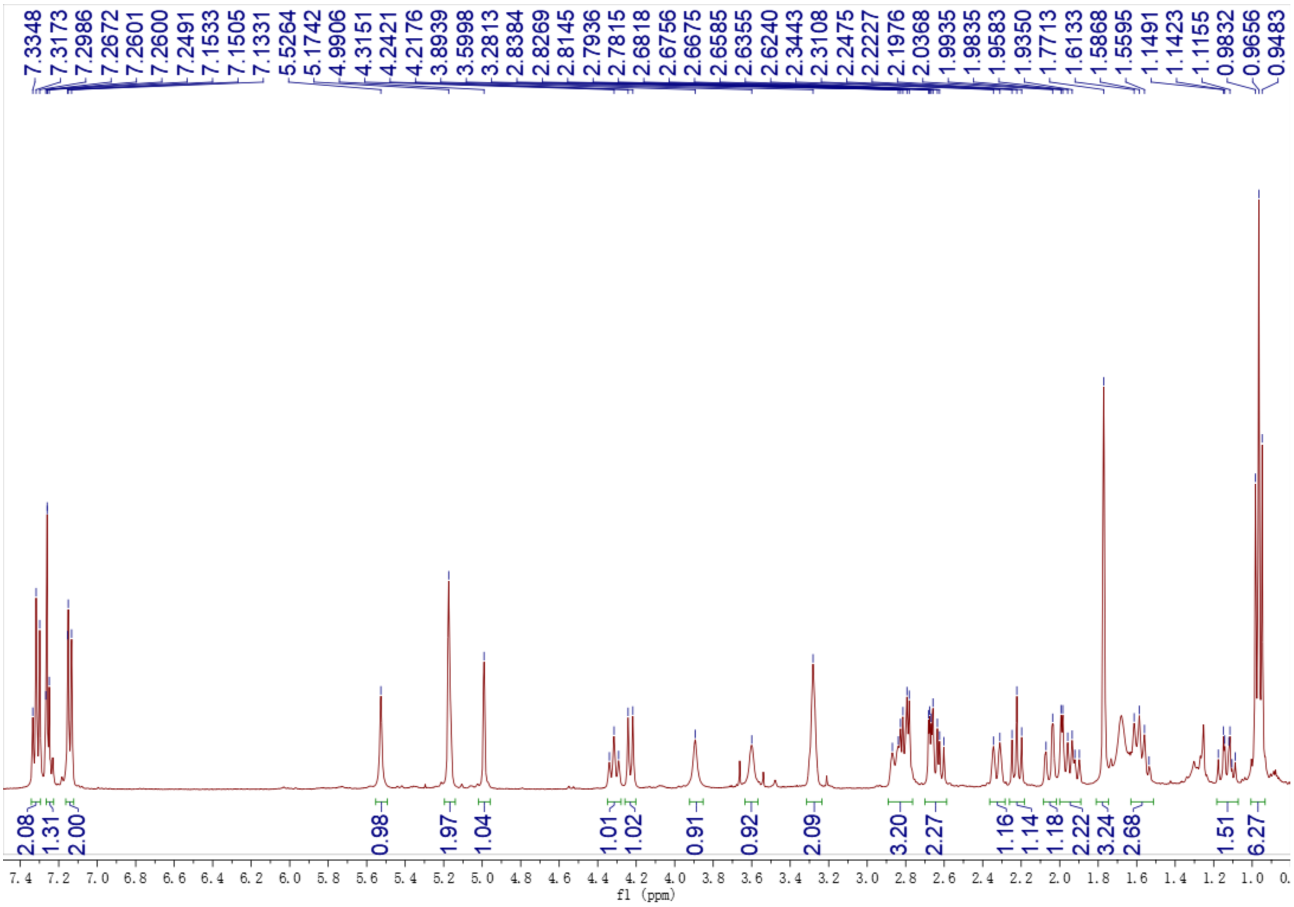

Spectrum S44. ^{13}C NMR and DEPT spectra of **8** in $CDCl_3$

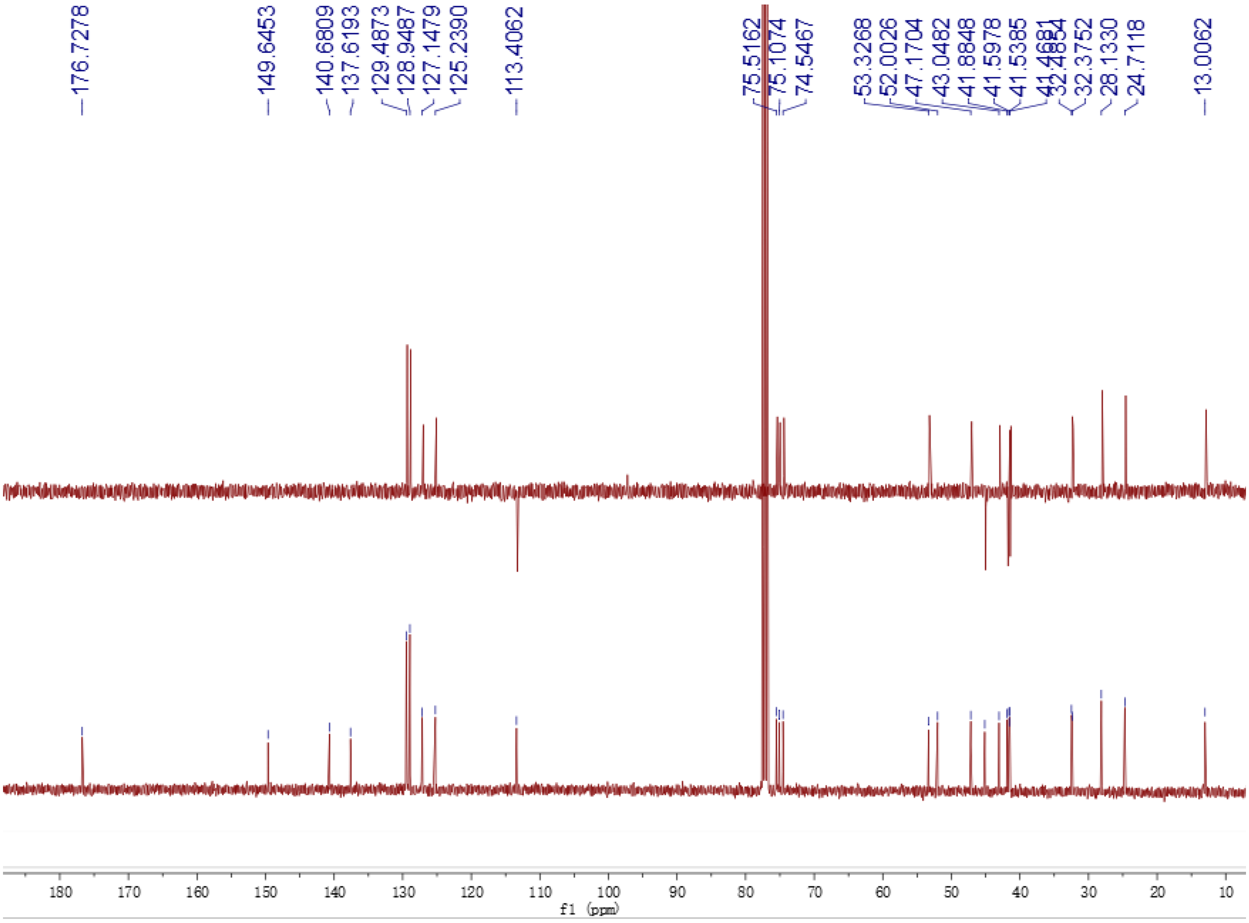

Spectrum S45. ^{1}H NMR spectrum of **9** in $CDCl_3$

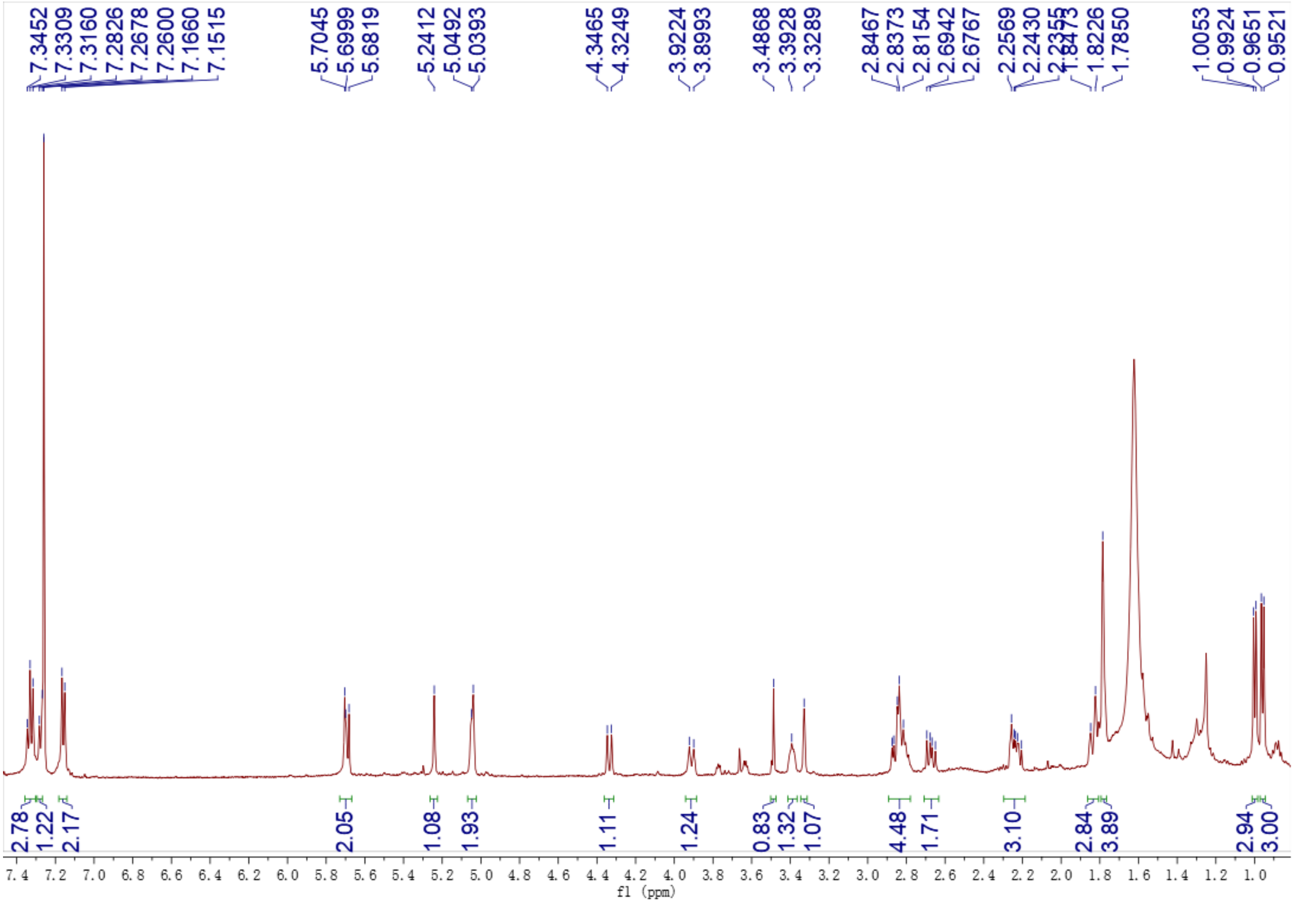

Spectrum S46. ^{13}C NMR and DEPT spectra of **9** in $CDCl_3$

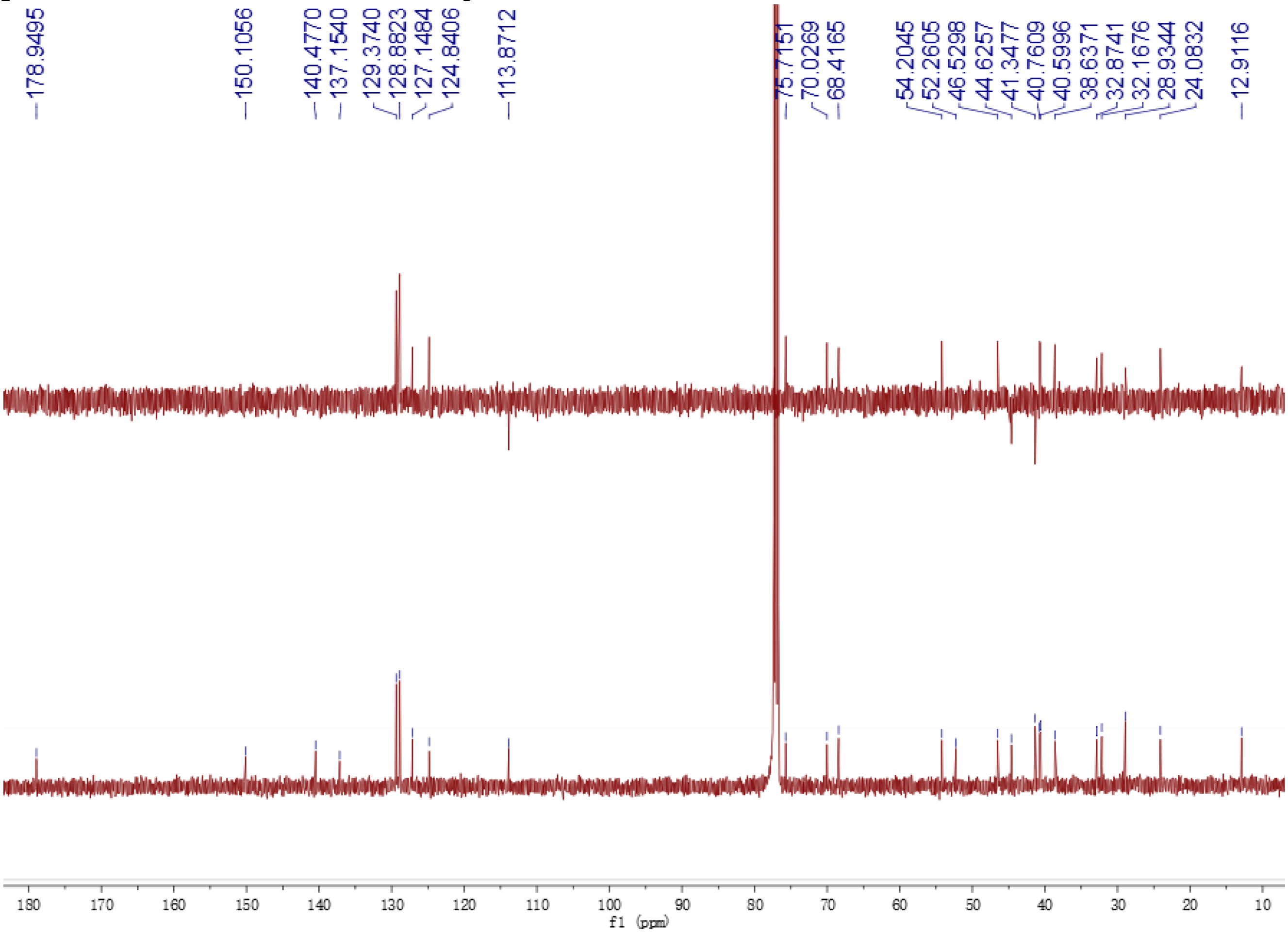

Spectrum S47. ^{1}H NMR spectrum of **10** in $CDCl_3$

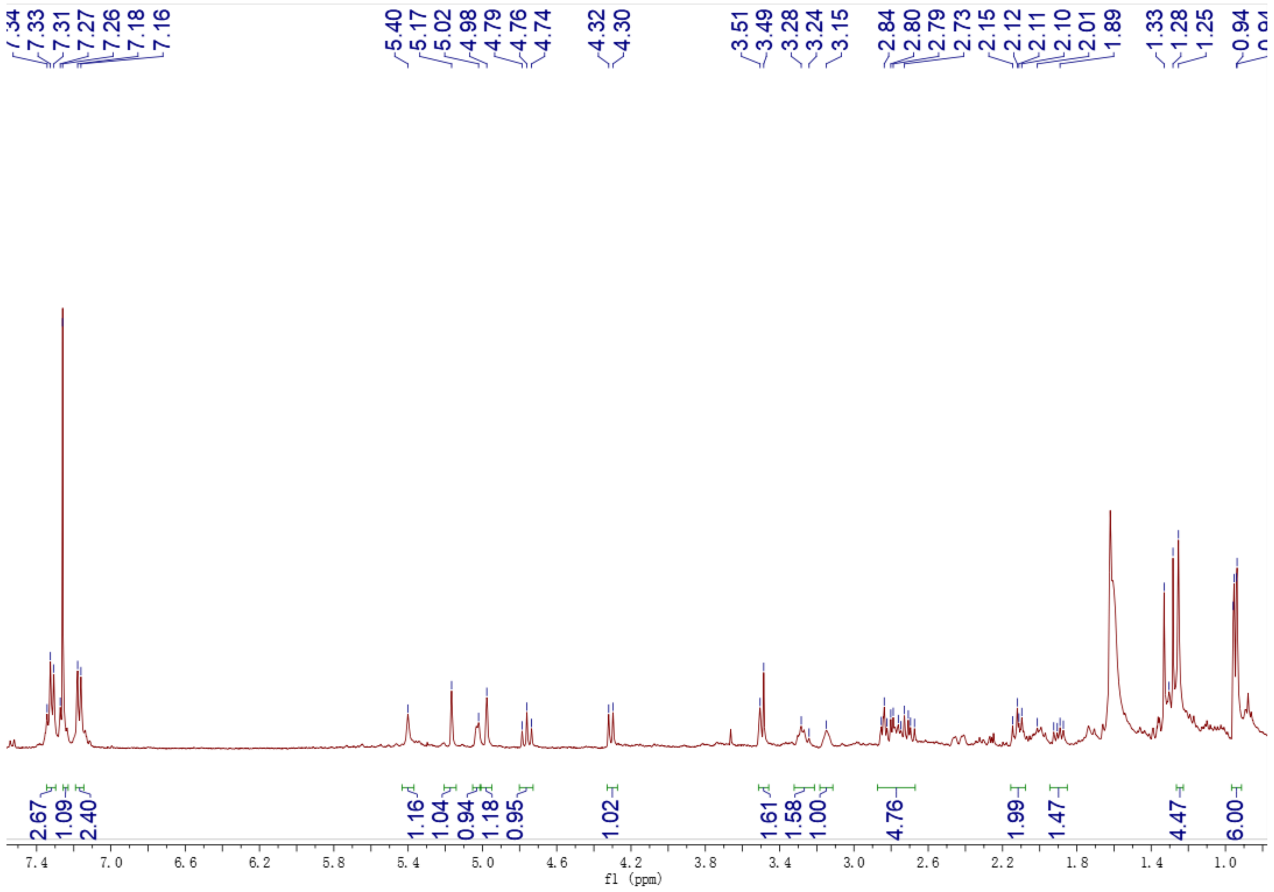

Spectrum S48. ^{13}C NMR and DEPT spectra of **10** in $CDCl_3$

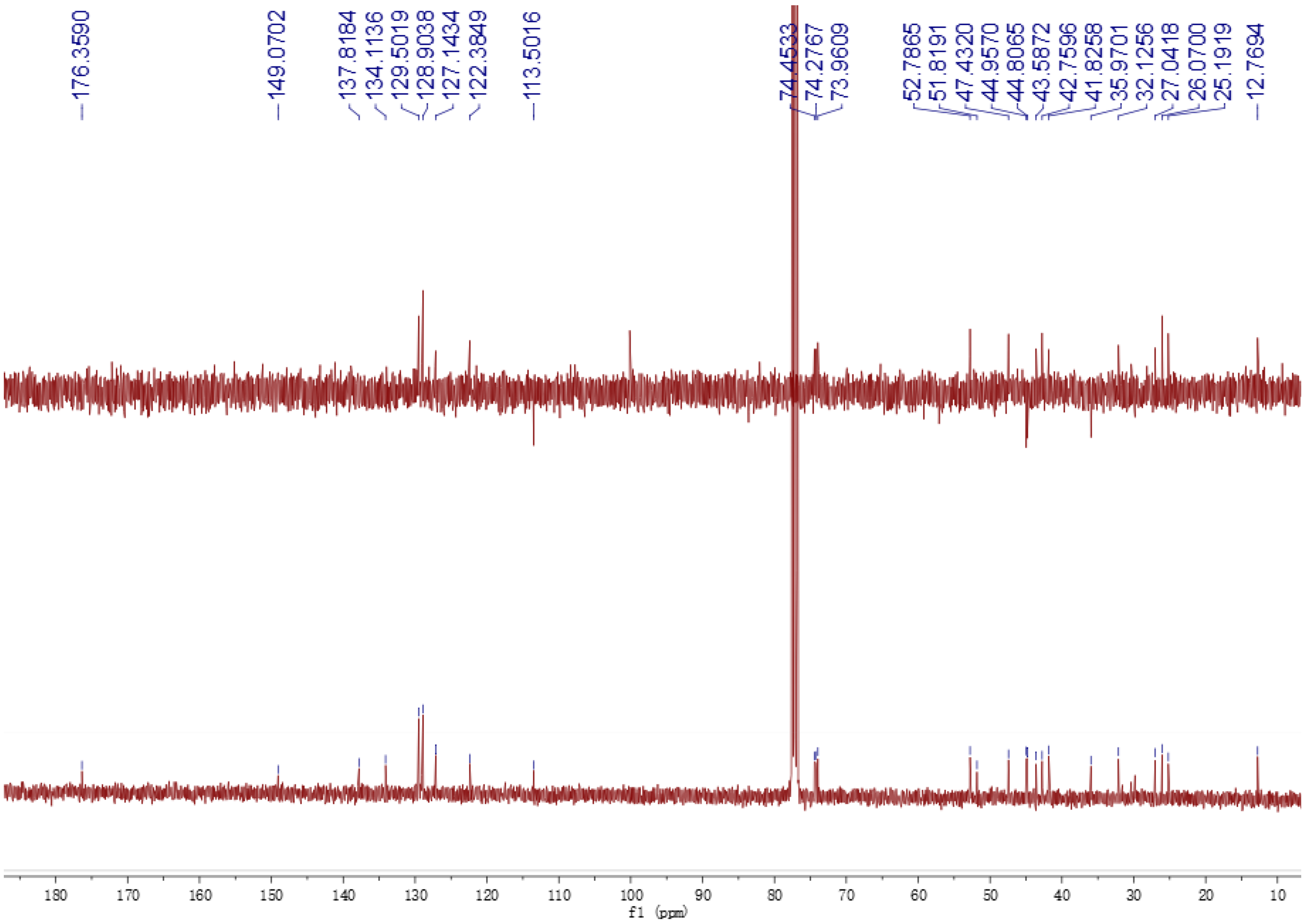

S4. HRESIMS spectra of 1–7

Spectrum S49. HRESIMS spectrum of **1**

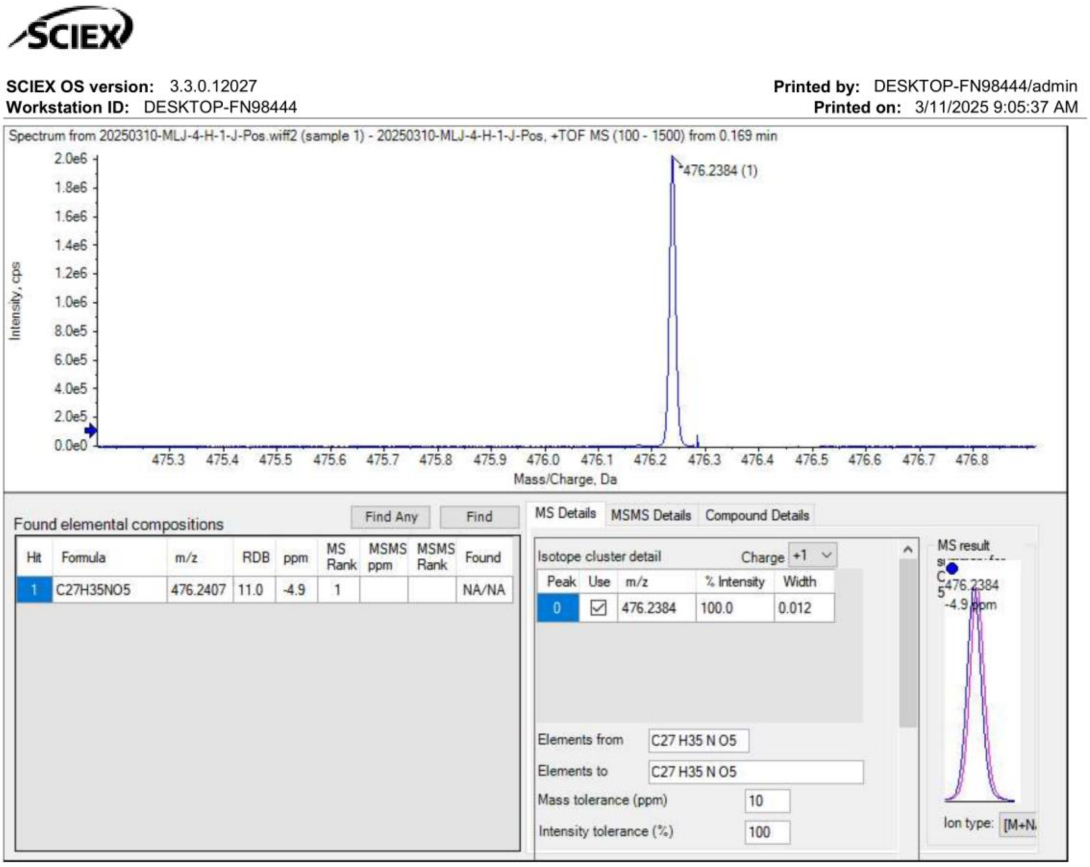

Spectrum S50. HRESIMS spectrum of **2**

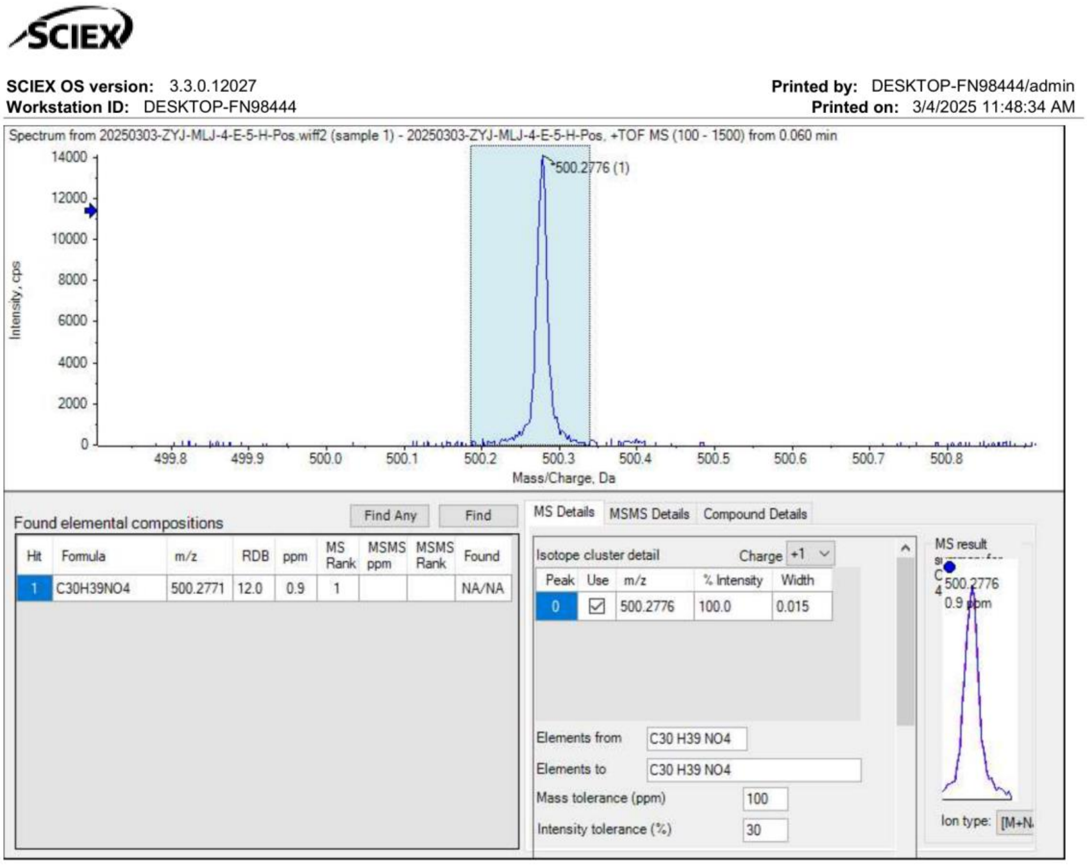

Spectrum S51. HRESIMS spectrum of **3**

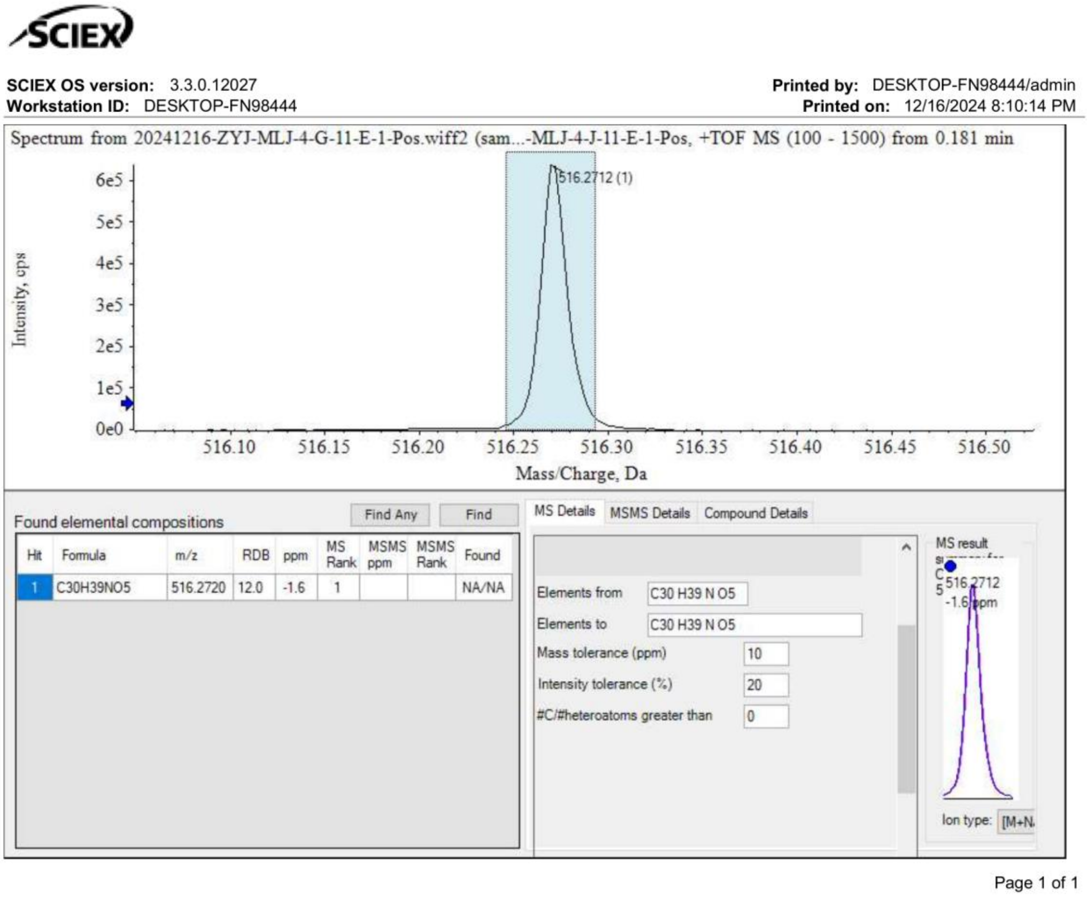

Spectrum S52. HRESIMS spectrum of **4**

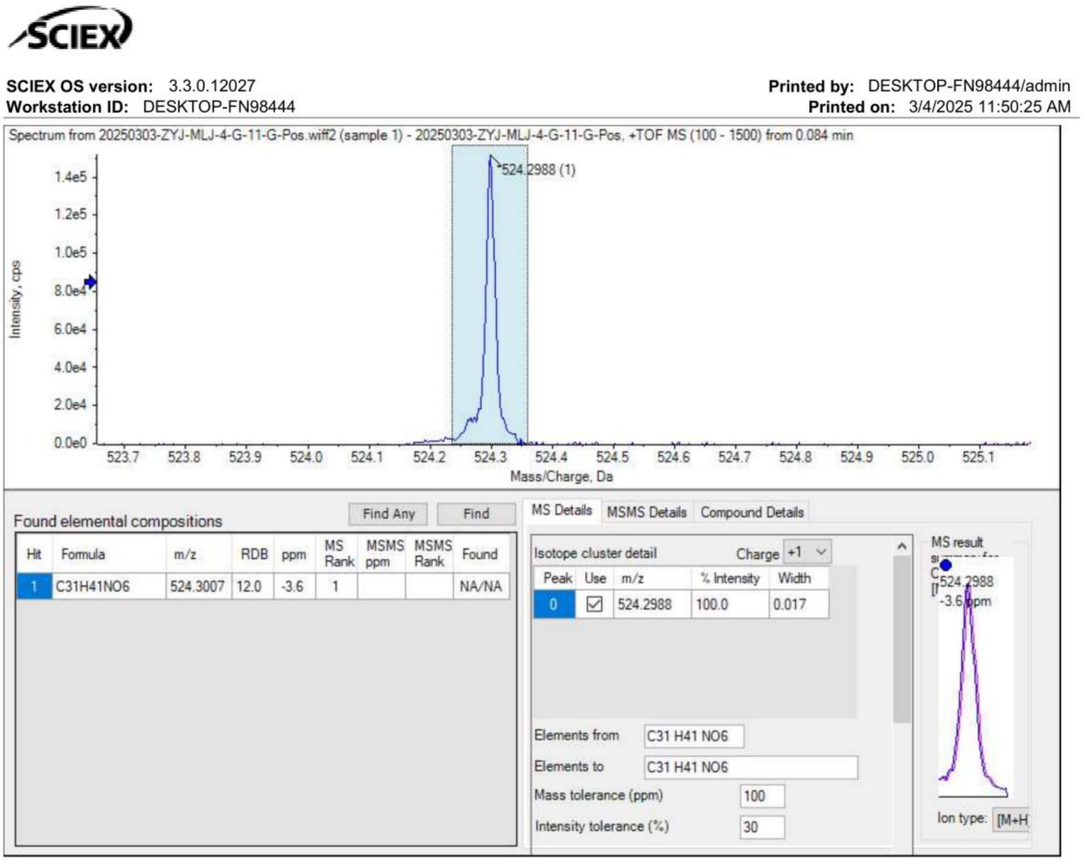

Spectrum S53. HRESIMS spectrum of **5**

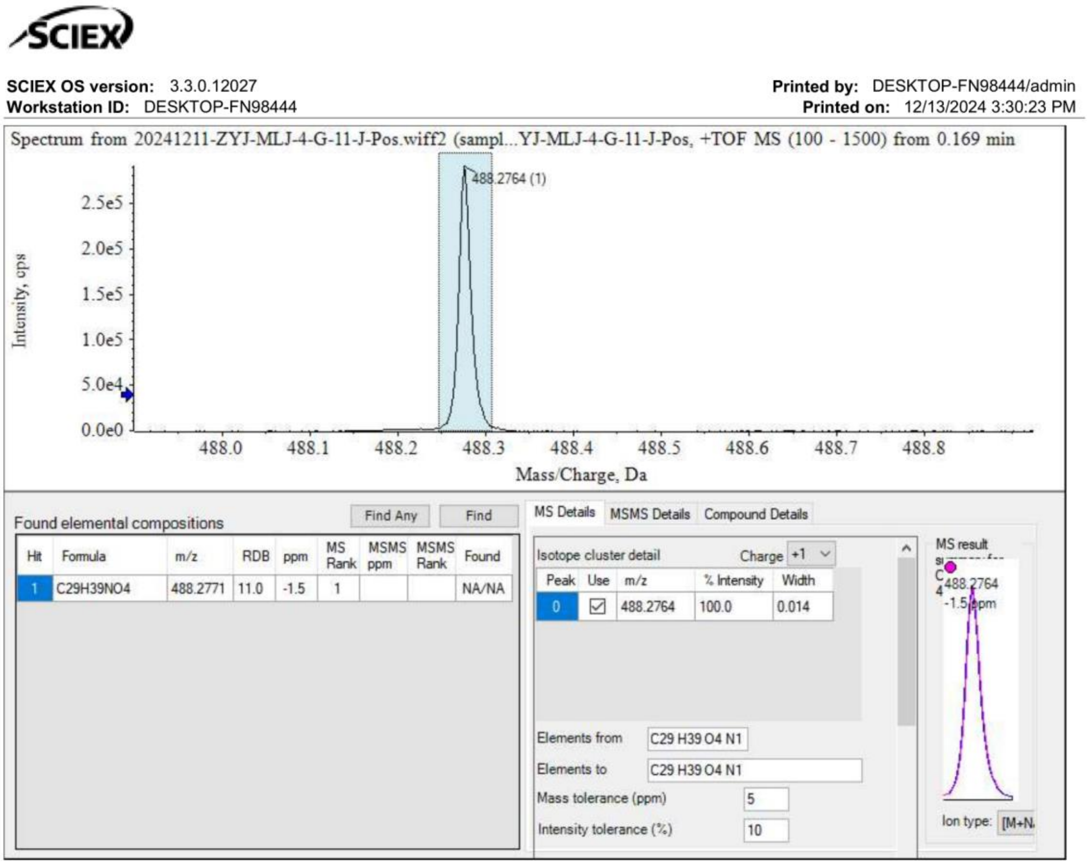

Spectrum S54. HRESIMS spectrum of **6**

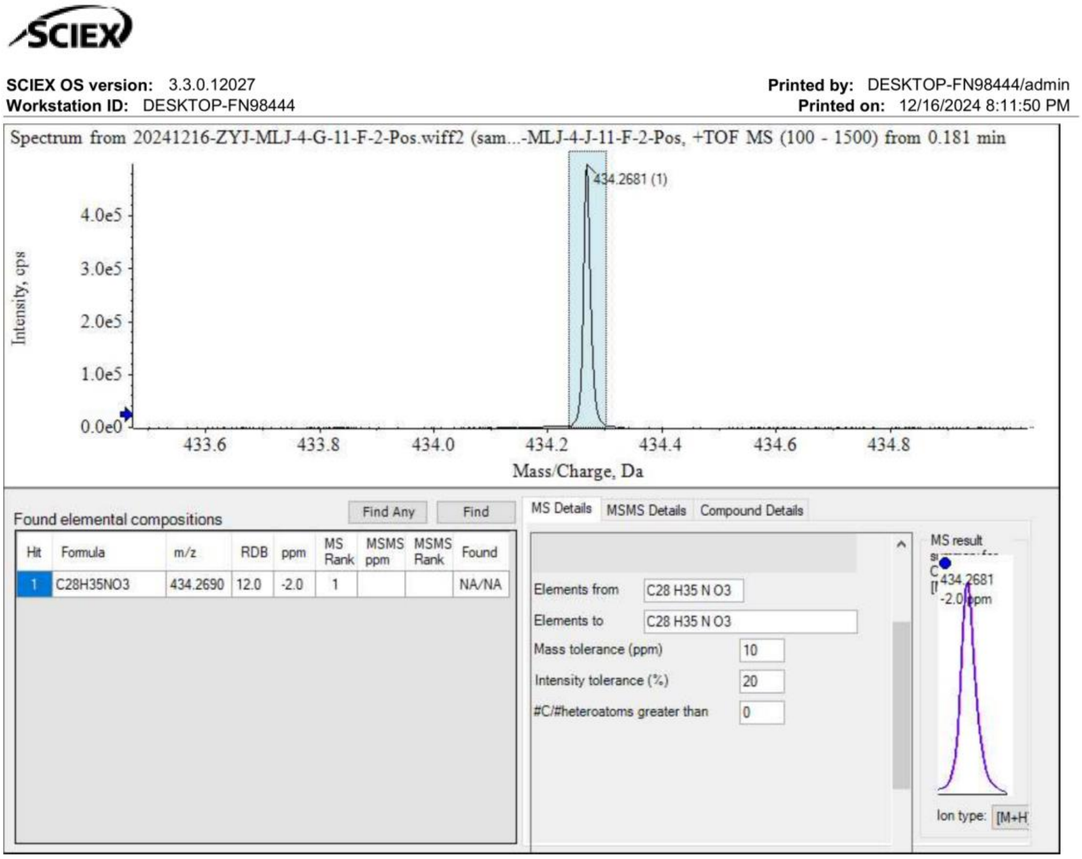

Spectrum S55. HRESIMS spectrum of **7**

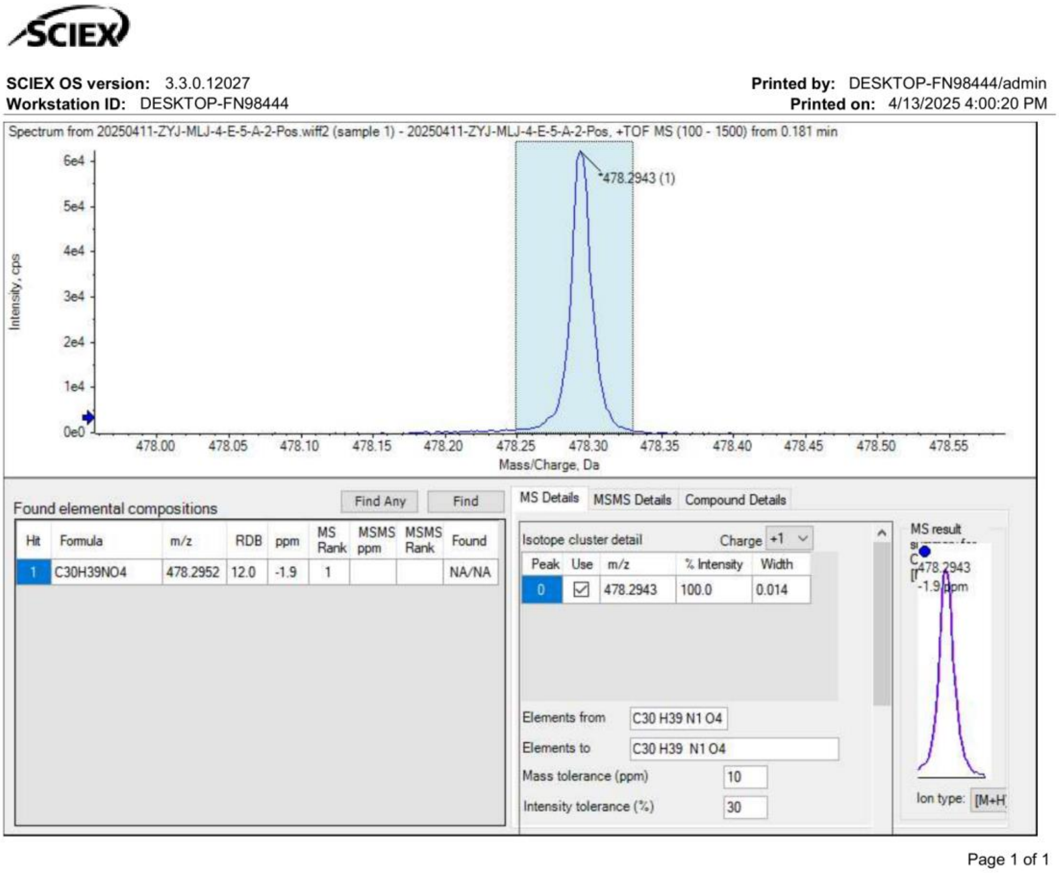

S5. IR spectra of **1–7**

Spectrum S56. The IR spectrum of **1**

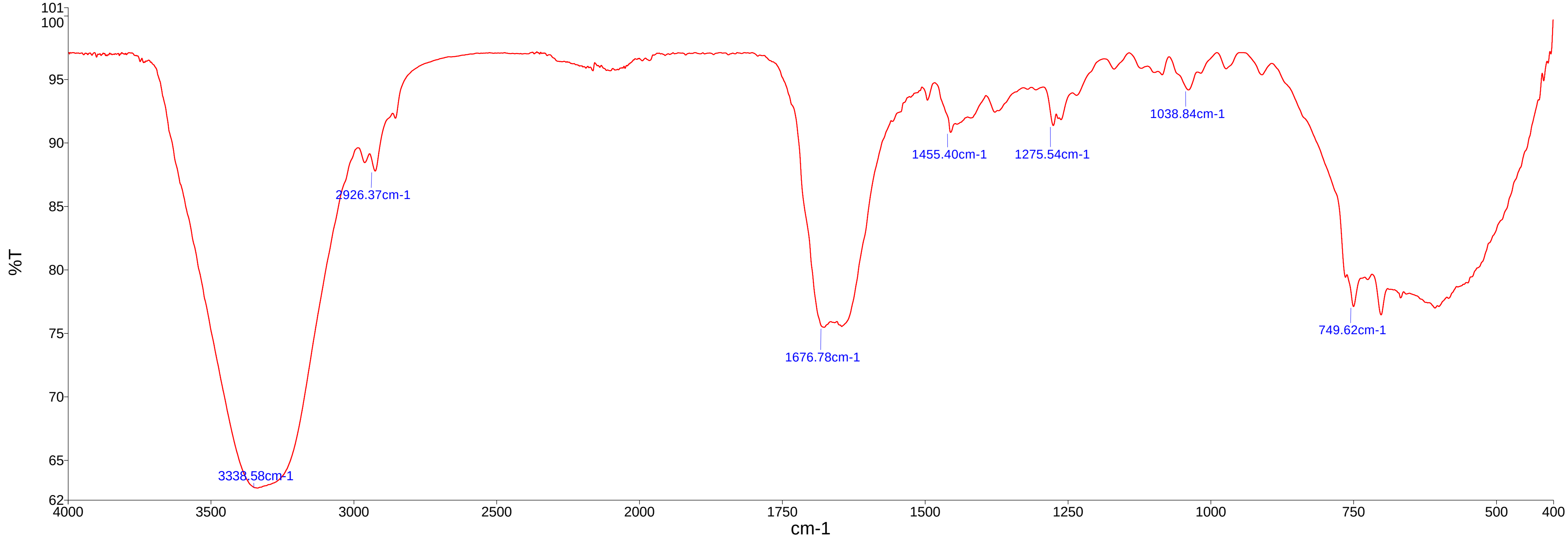

Spectrum S57. The IR spectrum of **2**

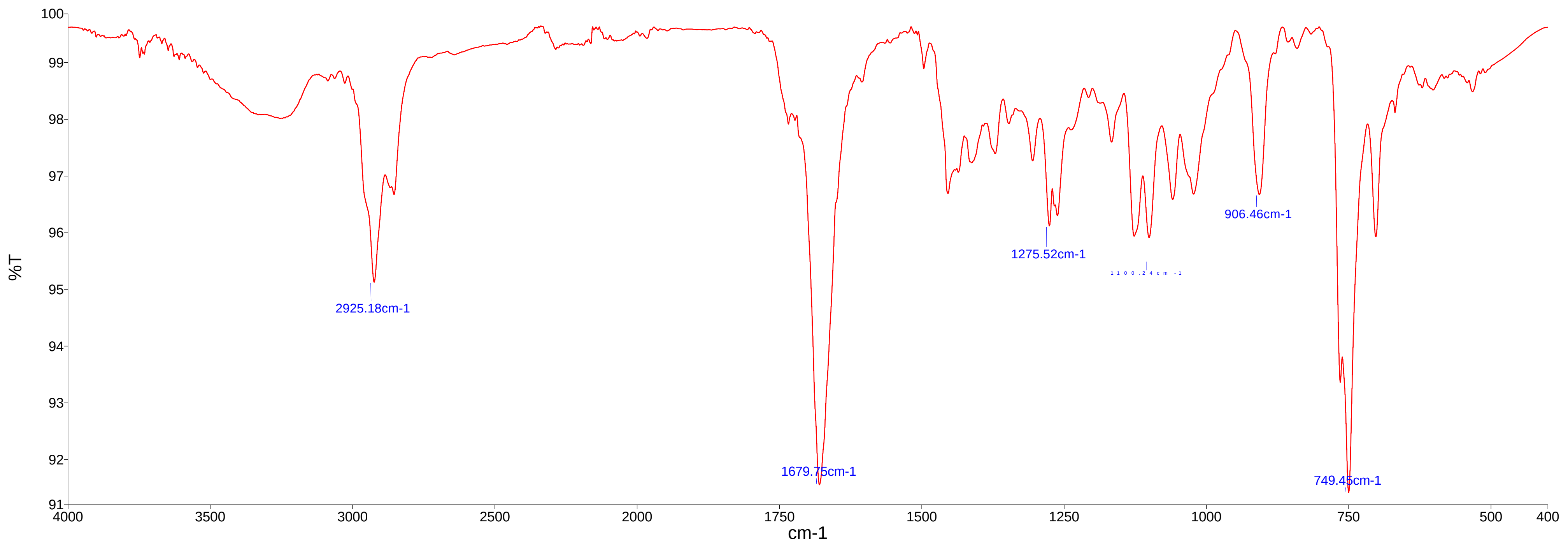

Spectrum S58. The IR spectrum of **3**

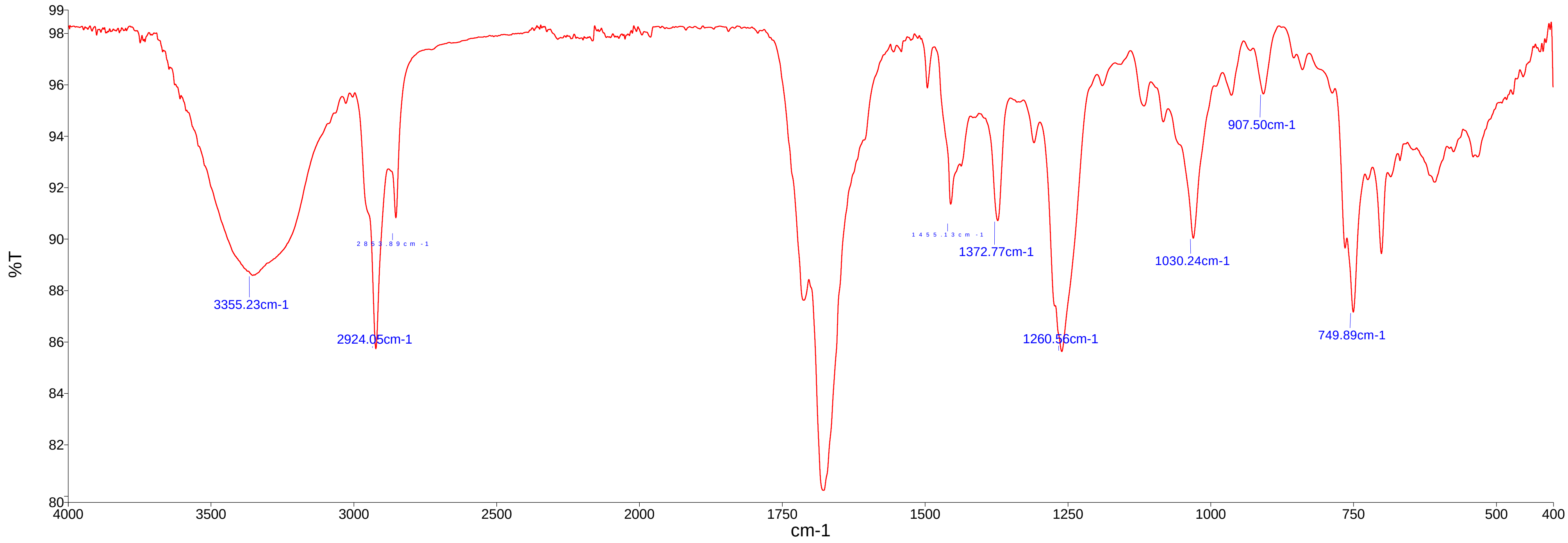

Spectrum S59. The IR spectrum of **4**

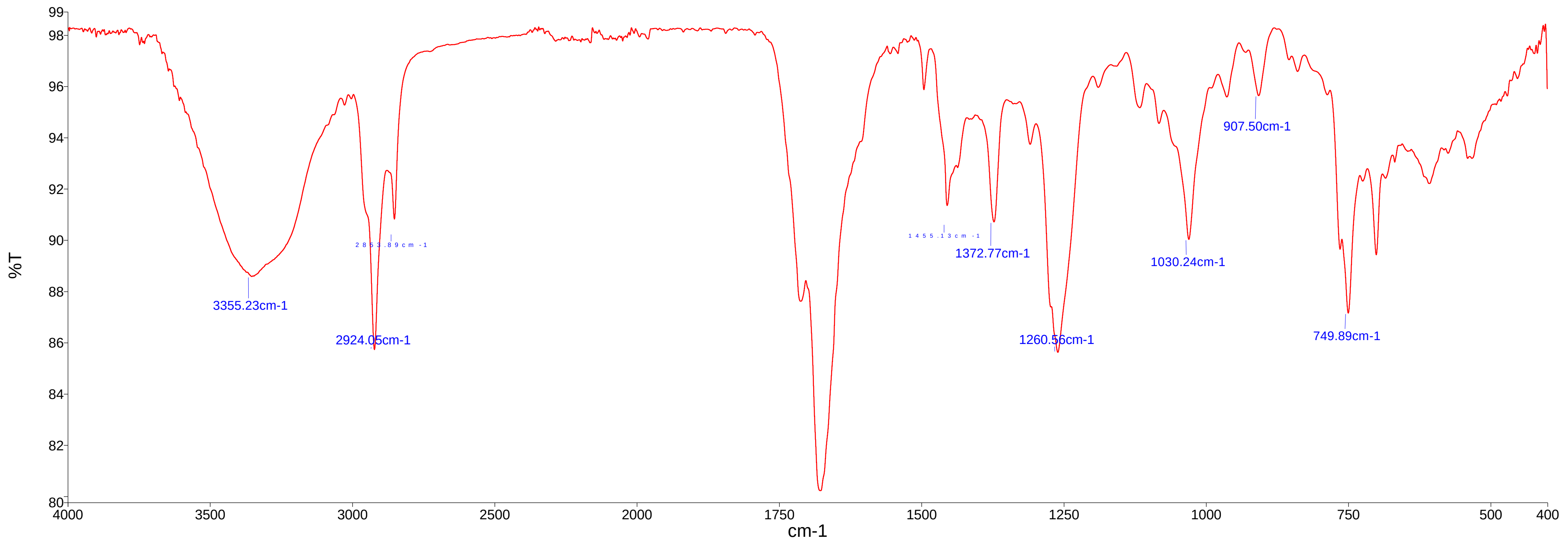

Spectrum S60. The IR spectrum of **5**

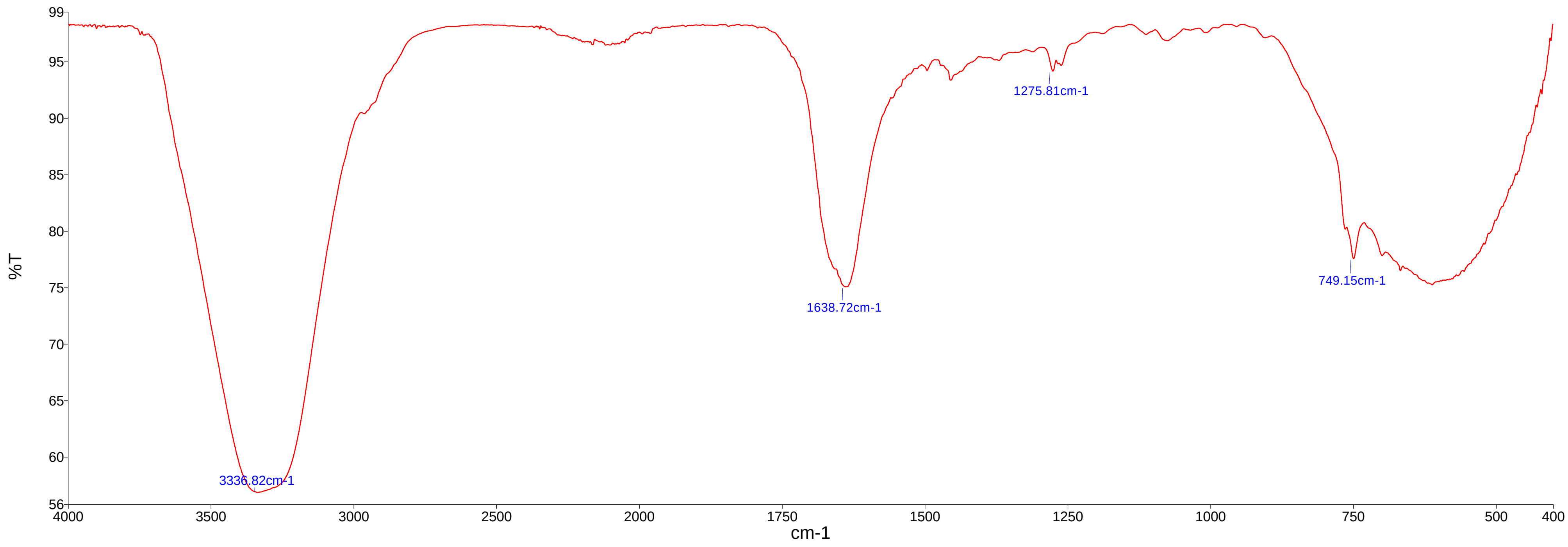

Spectrum S61. The IR spectrum of **6**

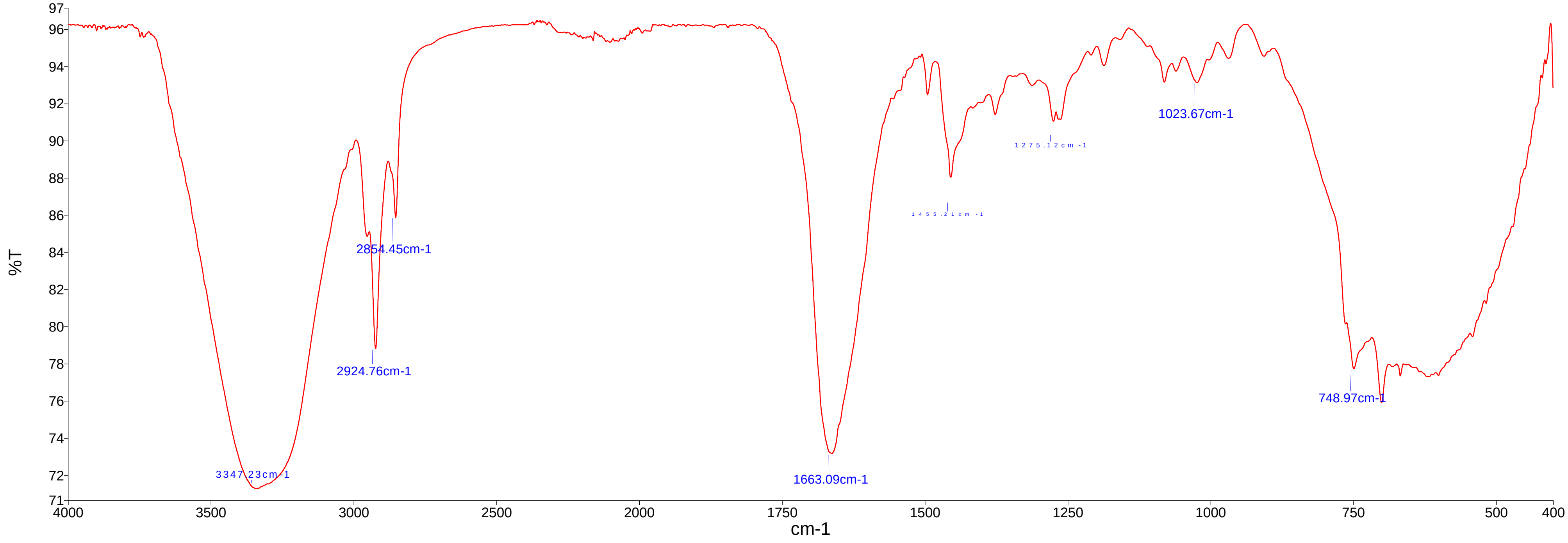

Spectrum S62. The IR spectrum of **7**

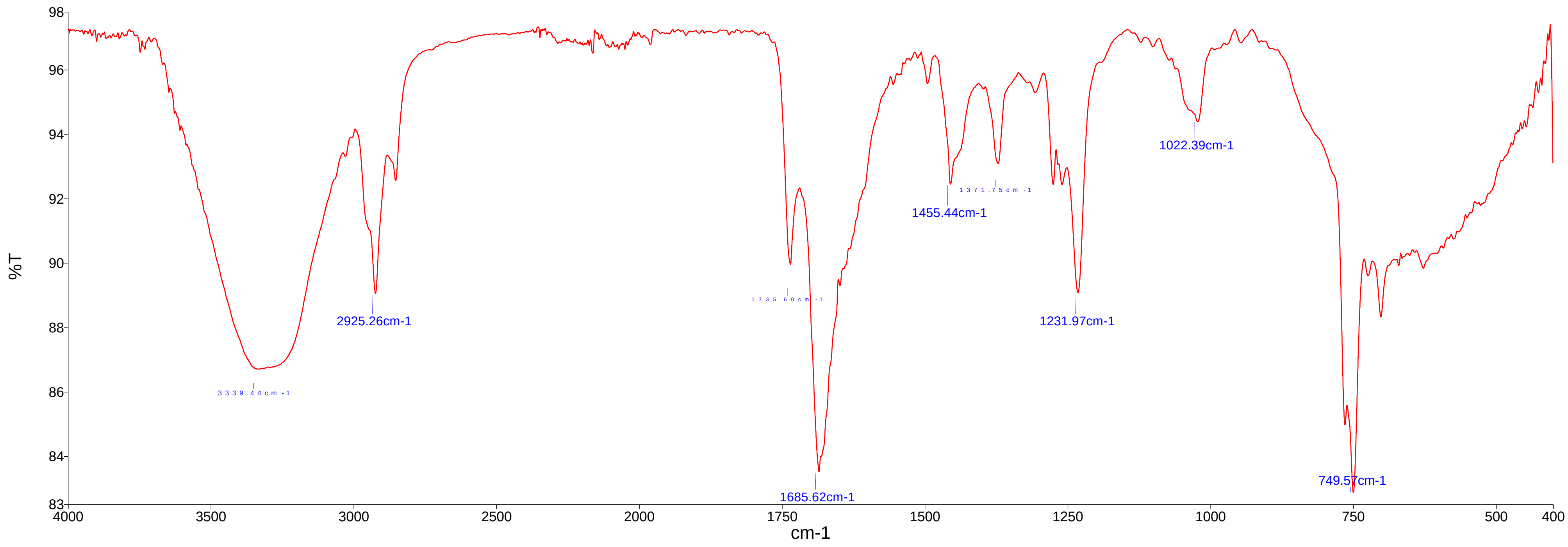

Supplement: Supplementary file 1 — Supplementary Material 1. Figures related to the article; ECD calculation for 1−3, and 7; 1D and 2D NMR (in CDCl3), HR-ESI–MS, and IR spectra of 1–7; 1H and 13C NMR (in CDCl3) spectra of 8–10 (PDF). [file 13659_2025_572_MOESM1_ESM.pdf]
